# Recycling of clastics in coastal areas inferred from quantitative analysis of reworked radiocarbon samples

**DOI:** 10.1038/s41598-021-04660-3

**Published:** 2022-01-13

**Authors:** Susumu Tanabe, Toshimichi Nakanishi, Rei Nakashima

**Affiliations:** 1 Geological Survey of Japan, AIST, Central 7, Higashi 1-1-1, Tsukuba, 305-8567 Japan; 2 Museum of Natural and Environmental History, Shizuoka, Oya 5762, Suruga-ku, Shizuoka, 422-8017 Japan

**Keywords:** Geology, Palaeontology, Sedimentology

## Abstract

Studies of the evolution of coastal lowlands since the Last Glacial Maximum (LGM) typically ignore radiocarbon data from sediment samples that have undergone reworking. However, these samples contain information on their sediment sources and the timing of their redeposition. We analyzed 738 radiocarbon dates obtained from shell and plant material in samples of post-LGM coastal sediment from north of Tokyo Bay, Japan. Of these samples, 245 (33%) were reworked. Furthermore, the percentage of reworked samples and their average age offsets increased with the depth of the water environment (terrestrial, 15% and 360 ± 250 years, respectively; intertidal, 26% and 470 ± 620 years; subtidal, 39% and 550 ± 630 years). Taking these radiocarbon samples as a proxy for clastic material, our results imply that channel erosion accounted for relatively little clastic removal in the terrestrial and intertidal environments over short timescales, whereas ~ 40% of clastics were removed by storm winnowing and transported in stepwise fashion to deeper water over longer timescales and ~ 60% in the subtidal environment were transported by floods directly from river mouths. These findings imply that radiocarbon ages from reworked samples can be used to quantify clastic recycling processes and their history in coastal areas.

During the Last Glacial Maximum (LGM) at 20.5 cal kyr BP (ka), the eustatic sea level fell to 130 m below its present level^1^. During this period of lowered sea level, incised valleys formed within the present coastal lowlands that were subsequently filled by riverine sediment as sea level rose during the last deglacial^2^. These post-LGM incised valley fills consist of late Pleistocene fluvial and Holocene marine sediments^3–5^.

Radiocarbon chronology is essential to reconstruct the evolution of post-LGM incised valley fills in coastal lowlands. Extensive radiocarbon dating performed in studies of coastal lowlands along the Rhine-Meuse^6,7^, Changjiang^8^, Po^9^, and Tone^10^ rivers has allowed the evolution of post-LGM incised valley fills to be reconstructed in detail. However, these studies have also found that a notable proportion of radiocarbon samples show indications of sediment reworking, namely by yielding ages older than those of underlying samples, and these samples have been ignored when reconstructing the evolution of post-LGM incised valley fills.

It is well known that bulk sediments yield considerably older ages than their depositional age^11,12^. However, few studies have systematically determined the percentage of reworked samples among all samples (reworked percentage) and the offsets between the ages of reworked samples (reworked ages) and depositional ages (age offsets) in coastal sediments by considering paleoenvironmental information from fossil shell and plant material in reworked samples^13–15^. In the sediment sequences of the Holocene strandplain system in Japan, for example, it has been shown that shells with anomalously shallow habitats yield anomalously old radiocarbon ages that differ from ages of stratigraphically adjacent samples by hundreds of years^13^. In addition, plants of terrigenous origin are sometimes found in marine sediments. These facts imply that if we regard radiocarbon-dated shells and plants as clastic materials, samples of reworked sediment can yield information on their supply source and depositional timing by comparison with the radiocarbon-dated samples that indicate true depositional ages.

Source-to-sink studies commonly rely on provenance analysis of sand^16^, chemical analysis of mud^17^, reconstruction of sediment accumulation rates and volumes by using radionuclides (^137^Cs, ^234^Th, and ^210^Pb)^18,19^, and combinations of these methods^20^ to clarify the sources, depocenters, and recycling histories of sediments. One recent study has traced the spread of sand grains from rivers to the coastal marine environment by using the optically stimulated luminescence dates of sand grains^21^. In deltas, episodes of clastic recycling are well characterized by modern observations^22^. However, these methods offer limited means of quantifying reworking rates or the timing of redeposition in a mixture of sand and mud formed by multiple hydraulic processes.

In this study, we compiled and analyzed 738 radiocarbon dates obtained on sediment samples from post-LGM incised valley fills in the plains north of Tokyo Bay during the last 20 years. About one-third (245) of these samples were reworked. By extracting the reworked percentage, age offset, depositional age, paleoelevation, and sand content of these reworked samples, we have achieved new quantitative insight into the processes of clastic recycling in this coastal area.

## Coastal lowlands north of Tokyo Bay

**Figure 1:**
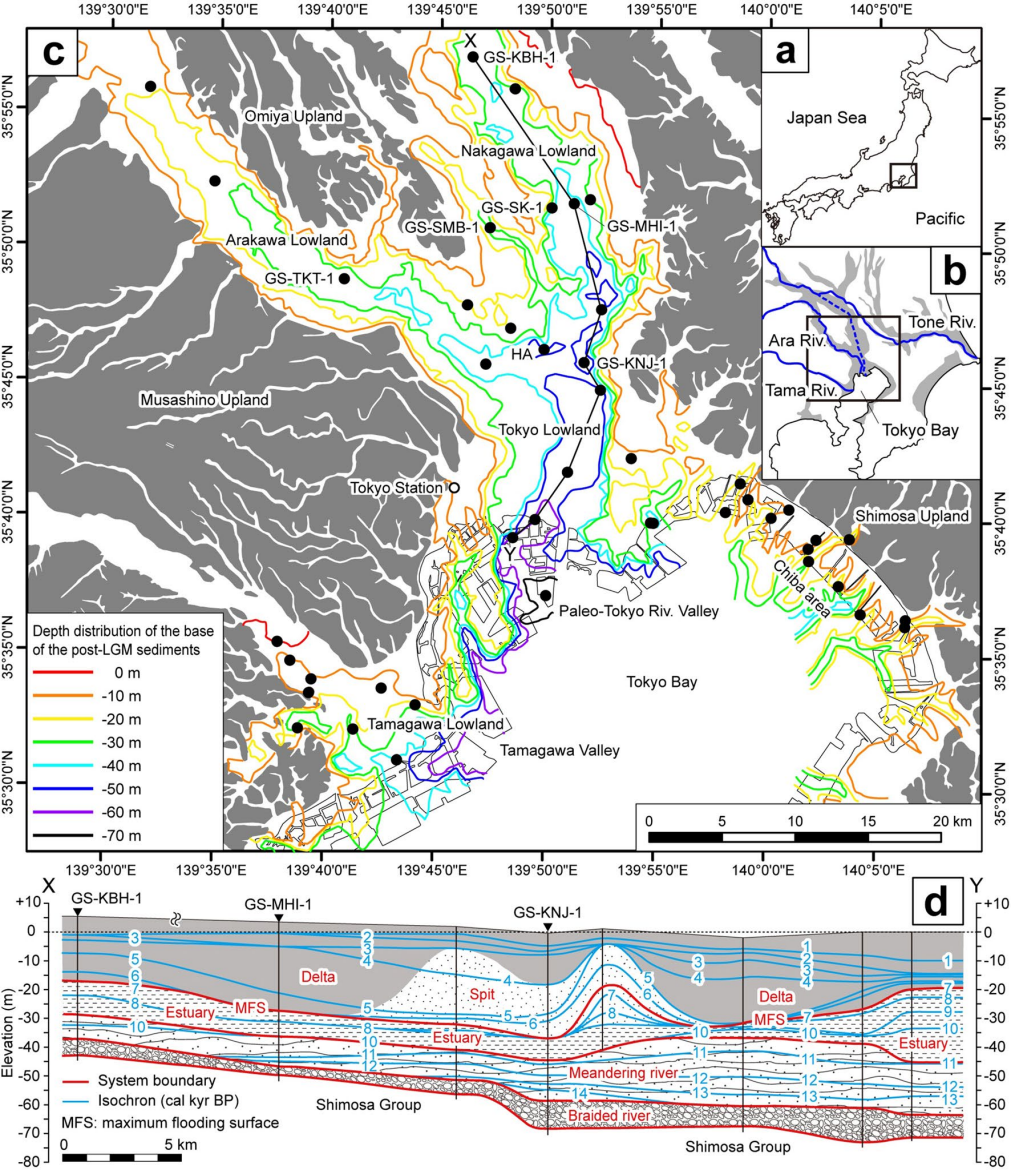
Location maps of the study area in (**a**) the Japanese Islands and (**b**) the Kanto region. Gray areas denote coastal lowlands. The dashed blue line indicates the historical course of the Tone River before its diversion. (**c**) Distribution of post-LGM incised valley fills in the study area^27^. Pleistocene uplands are shown in gray. Filled black circles denote coring sites. (**d**) Chronostratigraphic cross section X–Y (location in **c**) showing the post-LGM incised valley fills in the Tokyo and Nakagawa lowlands^10^.

Tokyo Bay, in the Kanto region of central Japan, extends over an area of 922 km^2^ (Fig. 1a, b). Tokyo Bay has a mean wave height and tidal range of 0.3 m and 1.8 m, respectively, and according to the scheme of Davis Jr. & Hayes^23^ can be classified as a low-energy tide-dominated coastal environment. However, the Tone River, which has the largest catchment in the Japanese Islands, historically flowed into Tokyo Bay until the seventeenth century, when it was diverted to the east to prevent flooding in the Tokyo urban area (Fig. 1b)^24^. The present Tone River has a drainage area of 16,840 km^2^, runoff of 8.7 km^3^/yr (276 m^3^/s), and sediment discharge of 3 Mt/yr (95 kg/s)^25^. As with other Japanese rivers, the runoff and sediment discharge of the Tone River are governed by precipitation during the rainy season and typhoons. During the category 2 typhoon in 1947, for instance, the runoff of the Tone River increased to 17,000 m^3^/s. The largest river flowing into Tokyo Bay today, in terms of runoff, is the Tama River, which has a catchment of 1,240 km^2^, runoff of 40 m^3^/s, and sediment discharge of 0.57 Mt/yr (18 kg/s) (Fig. 1b)^26^.

The seafloor sediment in Tokyo Bay consists mainly of mud from the coast to the center of the bay at –30 m (all depths are reported relative to mean sea level at the Tokyo Peil)^26^. In the abandoned river mouth of the pre-diversion Tone River, delta-front sand is distributed at depths shallower than –12 to –13 m^10^.

During the LGM, valleys were incised to –70 m in the Tokyo lowland and to –60 m in the Tamagawa lowland (Fig. 1c)^27^. Post-LGM fills in these incised valleys unconformably overlie the middle to late Pleistocene Shimosa Group; in ascending order, these fills consist of braided-river, meandering-river, estuary, spit, and delta systems. The braided-river system consists of gravel beds of braided-river sediments (facies BR); the meandering-river system consists of alternating sand and mud beds of meandering-river sediments (facies MR); the estuary system consists of upward-fining sand and mud beds of tidal-flat (facies TF) and estuary-front (facies EF) sediments; the spit system consists of sand beds of spit sediments (facies SP); and the delta system consists of upward-coarsening mud and sand beds of delta-front (facies DF), modern tidal-flat (facies MT), and modern fluvial (facies MF) sediments (Fig. 1d)^10,28^. The spit system is confined to the western tip of the Shimosa Upland (Fig. 1c). In the Tokyo, Nakagawa, Arakawa, and Tamagawa lowlands (Fig. 1c), an amalgamated braided river system was formed during the LGM lowstand^10,29^. The retrograding meandering-river and estuary systems developed during the transgressive phase from 14 to 7 ka, and the prograding delta system formed during the regressive phase after 8 ka (Figs. S1, S2)^10,28^. The maximum flooding surface (MFS), which separates the transgressive and regressive systems, is dated to 8 ka in the Arakawa and Tamagawa lowlands and 7 ka in the Tokyo and Nakagawa lowlands (Fig. 1d)^10,28,30^. The timing of formation of the MFS was determined by the balance in rates of sea-level rise and sediment discharge^31^. In the Arakawa and Tamagawa lowlands, the MFS formed earlier than the highest peak of sea level at 7 ka in Tokyo Bay because of abundant sediment discharge from rivers; however, in the Tokyo and Nakagawa lowlands, the MFS coincides with the highest peak of sea level owing to a lack of riverine sediment discharge (Figs. S1, S2)^10^.

## Results

**Figure 2:**
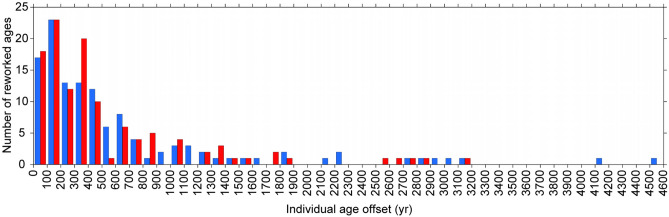
Frequency distribution of individual age offsets for samples of (blue) shell material and (red) plant material.

In this study, we compiled 757 radiocarbon dates from 45 cores (total length 2,193 m) obtained in the area north of Tokyo Bay (Fig. 1c, Table S1, S2). Of these dates, 19 were from the Shimosa Group and 738 were from the post-LGM incised valley fills. In this study, we regarded any ages younger than the age of the underlying horizon or the youngest age in the same horizon as depositional ages and other ages as reworked (Fig. S1). Among the 738 dates from the post-LGM incised valley fills, we regarded 493 (67%) as depositional ages and 245 (33%) as reworked ages. After excluding four dates from plant materials as outliers, with age offsets exceeding 7,000 years, we determined that the average age offset of the 241 reworked samples was 600 ± 740 years. Among these 241 samples, 197 (82%) had age offsets of less than 1,000 years (Fig. 2).

We compared the data from shell and plant materials as follows. Of the 241 reworked samples, 123 contained shells and 118 contained plant material, thus 36% of the 344 radiocarbon-dated shells and 30% of the 394 radiocarbon-dated plants were reworked. Shell dates had an average age offset of 670 ± 830 years, and 98 shells (80%) had age offsets less than 1,000 years. Plant dates had an average age offset of 530 ± 630 years, and 99 plants (84%) had age offsets less than 1,000 years. The age offsets of shells and plants thus had almost identical frequency distributions (Fig. 2). As described later, the age offsets of spit sediments were anomalously large. Excluding these age offsets resulted in an average age offset of 490 ± 570 years for shells, nearly the same as that of plants. Average age offsets for both shells and plants were available for facies EF and DF (Table S3). In facies EF, the average age offsets of shells and plants were 460 ± 320 and 680 ± 800 years, respectively. In facies DF, the average age offsets of shells and plants were 500 ± 680 and 600 ± 640 years, respectively; thus, on average, age offsets of plants were 100 to 220 years greater than those of shells. This difference has arisen because shells with younger age offsets occur more frequently than plants with age offsets in the same range (Fig. S3). On the other hand, the frequency distributions of shells and plants in facies EF and DF increase toward the younger age offsets (Fig. S3), as in the entire shell and plant dataset (Fig. 2). By taking account the difference between regional and global marine ^14^C ages (ΔR)^32^ of 60 to 120 years in Tokyo Bay^33^, the difference between age offsets of shells and plants become much smaller. This finding indicates that plant and shell materials behave approximately similarly when regarded as clastic materials.

To discuss the age offsets of shells more precisely, their burrowing depths must be considered. Of the 263 shells identified in this study, the species *Mya japonica* Jay has a maximum burrowing depth of 30 cm^34^. Other molluscs live in surficial habitats with burrowing depths of < 10 cm. The sediment accumulation rate of facies DF in core HA (Fig. 1c), which yielded *M. japonica*, is ~ 3 mm/yr^10^. This result means that an error range of < 100 years must be considered when assessing shell ages; this error magnitude is mostly negligible in this study.

**Figure 3:**
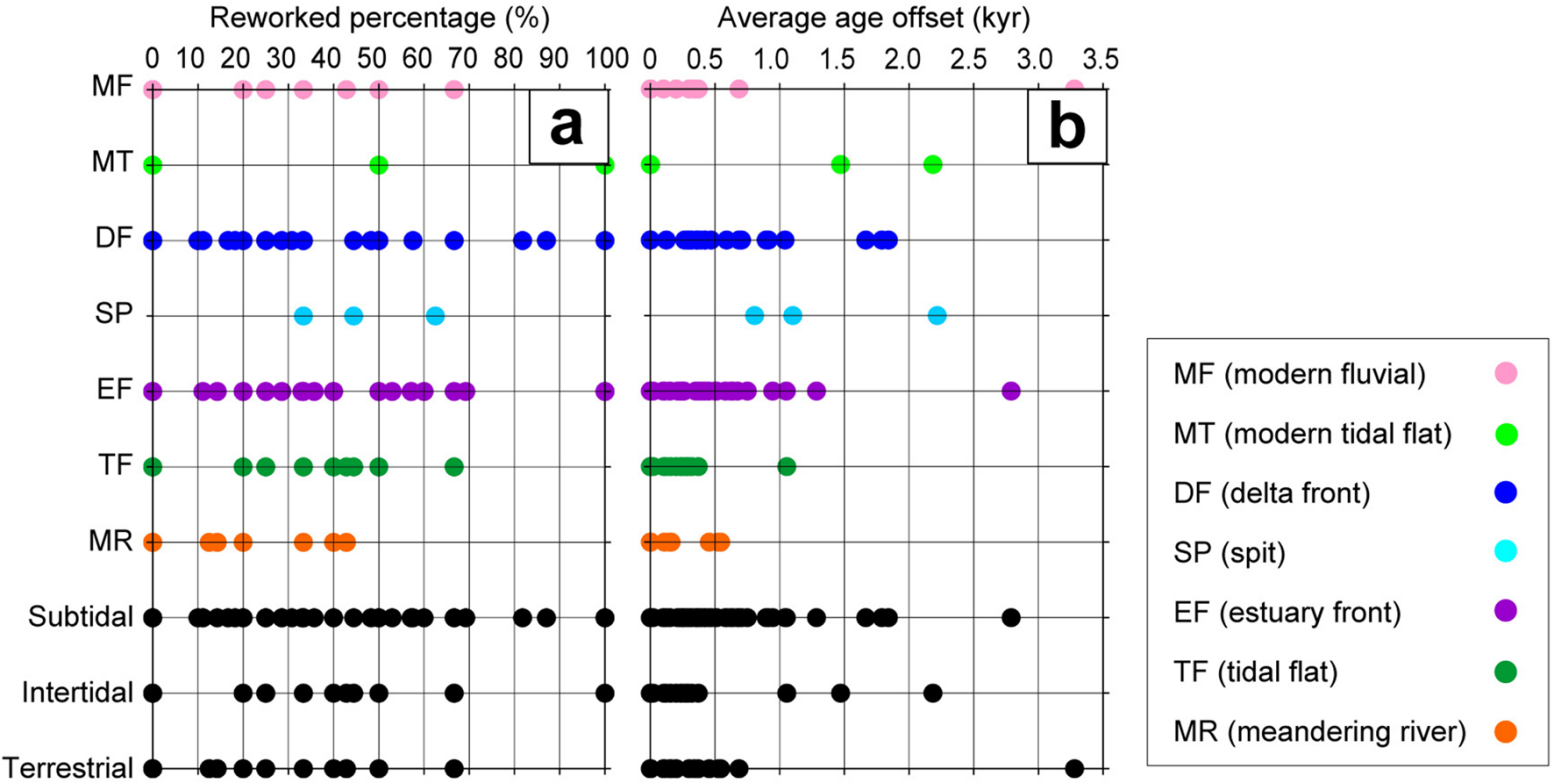
(**a**) Reworked percentages and (**b**) average age offsets in 45 cores classified by sedimentary facies and paleo-water depth.

The reworked percentages and average age offsets of sedimentary facies in the 45 cores were clearly related to water depth (Fig. 3, Table S4, S5). The reworked percentages and average age offsets increased from facies MR to DF and decreased from facies DF to MF (Fig. 3). Classified in terms of water depth, facies MR and MF are terrigenous, facies TF and MT are intertidal, and facies EF and DF are subtidal. The reworked percentages and average age offsets in these facies categories increased with water depth, being 15% and 360 ± 250 years, respectively, for terrigenous sediments, 26% and 470 ± 620 years for intertidal sediments, and 39% and 550 ± 630 years for subtidal sediments. The reworked percentage (50%) and average age offset (1,710 ± 1,340 years) of facies SP were especially large (Table S5). Facies MR, TF, and EF are transgressive sediments; facies SP comprises transgressive to regressive sediments; and facies DF, MT, and MF are regressive sediments; however, the reworked percentages and average age offsets of these sedimentary facies were not clearly related to the sea-level changes during the last deglacial (Figs. 3, S1).

**Figure 4:**
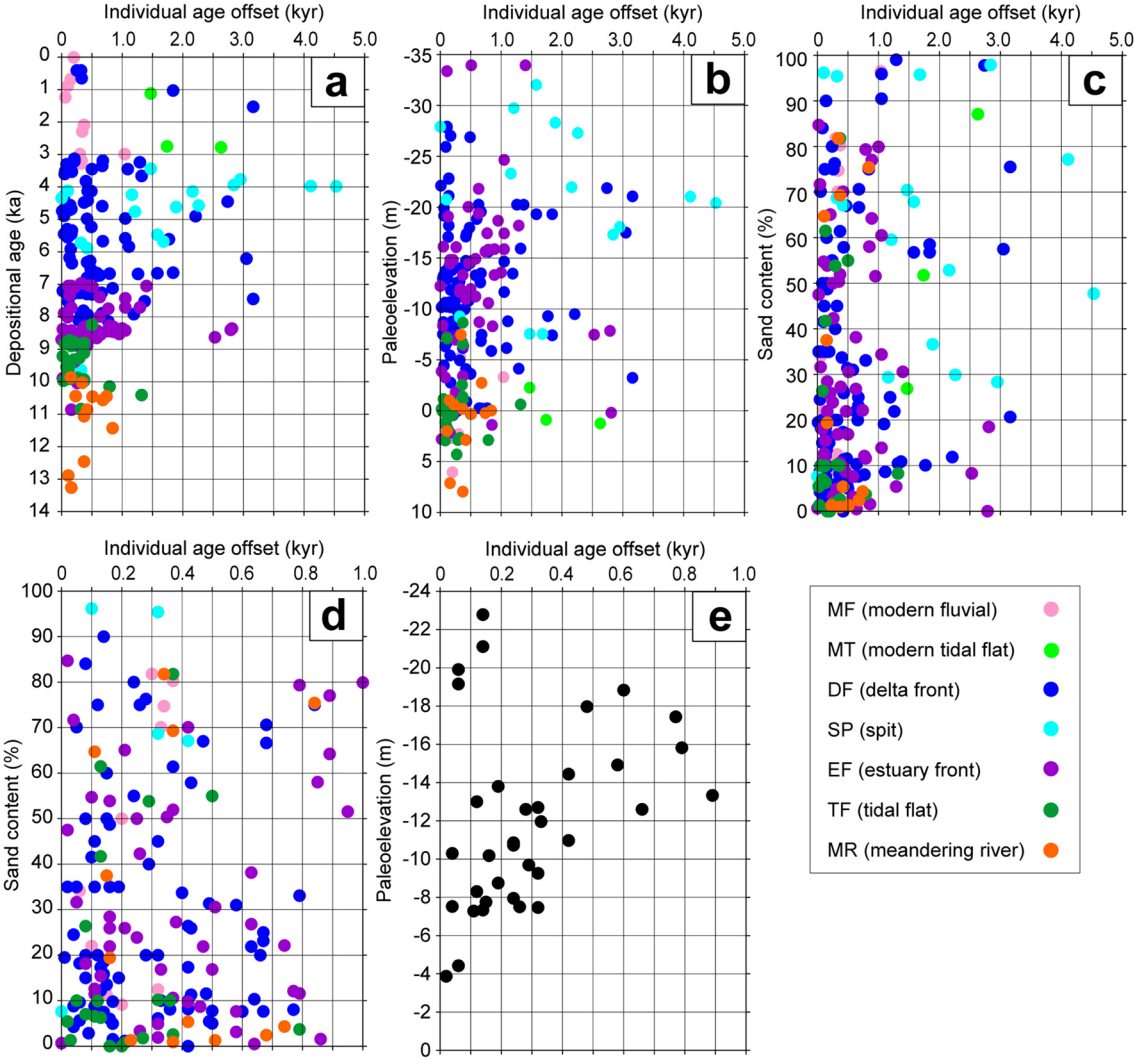
(**a**) Individual age offsets versus depositional age. (**b**) Individual age offsets versus paleoelevation. (**c**) Individual age offsets versus sand content. (**d**) Detail of **c** showing individual age offsets less than 1,000 years versus sand content. (**e**) Individual age offsets of *Potamocorbula* sp. specimens versus paleoelevation.

Figure 4 presents plots of the offset between the reworked and depositional ages of individual radiocarbon dated samples (the individual age offset) versus depositional age, paleoelevation, and sand content. The range of individual age offsets increased as depositional ages decreased from 14 to 4 ka and then decreased from 4 to 0 ka (Fig. 4a). Most of the samples with individual age offsets greater than 1,000 years were from facies SP and DF, and their depositional ages were from 7 to 4 ka. This age range matches the timing of the middle Holocene sea-level highstand in Tokyo Bay (Fig. S1). The range of individual age offsets increased for paleoelevations from + 10 to –20 m and decreased for paleoelevations from –20 to –35 m (Fig. 4b). The range of individual age offsets increased greatly from 0 to –20 m in facies EF, SP, and DF. The range of individual age offsets increased as the sand content decreased (Fig. 4c). This trend was especially strong for individual age offsets less than 1,000 years (Fig. 4d). These results suggest that the range of individual age offsets became larger during the middle Holocene sea-level highstand, as paleoelevations approached –20 m, and when sediments were composed of fine-grained mud.

A MFS is formed at a transition from a transgressive system to a regressive system, and is commonly characterized by relatively small sediment accumulation rates^31^. In the Arakawa lowland and Nakagawa lowland, the MFS was formed at 8 and 7 ka, respectively^10^. A plot of reworked percentages versus depositional ages suggests that the reworked percentage was greatest (68%) at 7.5 to 7.0 ka (Fig. S4). Of the 38 reworked radiocarbon samples deposited during 7.5‒7.0 ka, most were from cores GS-KBH-1, GS-MHI-1, GS-SMB-1, and GS-TKT-1 (Fig. 1c). In core GS-TKT-1 from the Arakawa lowland, 25 reworked radiocarbon samples were deposited after the formation of the MFS at 8 ka (Fig. S5). Only two reworked radiocarbon samples occurred at around the MFS in this core. In cores GS-KBH-1, GS-MHI-1, and GS-SMB-1 from the Nakagawa lowland, the depositional ages of eight reworked radiocarbon samples coincided with the MFS at 7 ka; however, only two to three reworked radiocarbon samples occurred at around the MFS in each core (Fig. S5). These results indicate that the high reworked percentage at 7.5 to 7.0 ka was induced by high-density radiocarbon dating in core GS-TKT-1, and was not related to the formation process and small sediment accumulation rate of the MFS. The reworked percentage was relatively high during 9‒3 ka (Fig. S4), which is consistent with the increase in paleo-water depth (Fig. 4a and b).

Average age offsets are predicted to be large in sedimentary facies with long depositional durations because of mixing of old clastic materials. To test this hypothesis, we calculated the depositional duration and average age offsets of sedimentary facies in 25 cores (Table S6, Fig. S6). No definite relationship between the depositional duration and the average age offsets could be observed (Fig. S6). The average age offsets increase from facies TF to facies EF and DF, suggesting a strong relationship with paleo-water depth.

## Discussion

Differences in reworked percentages and average age offsets in the terrigenous, intertidal, and subtidal environments can be interpreted by considering the depositional processes operating at each water depth.

Relatively small reworked percentages and average age offsets in the terrigenous and intertidal environments can be explained by erosion and redeposition of clastics trapped in floodplain and tidal-flat settings by lateral migration of fluvial and tidal channels (Fig. S2a, b). The reworked percentages and average age offsets in the intertidal environments are larger than those in the terrigenous environments because the lateral migration of tidal channel and truncation of old strata were relatively large in the intertidal environments (Fig. S2a, b). The much larger reworked percentages and average age offsets in facies SP can be explained by transport of old beach and shoreface deposits into deeper water environments. As sea level rose during the last deglacial sea-level rise, the beach and shoreface deposits along the Shimosa Upland were submerged and then were eroded by longshore currents during the middle Holocene sea-level highstand (Fig. S2c, d)^10^.

The reworked percentage, average age offset, and clastic transport in subtidal environments can be investigated by the use of juvenile shells of the mollusc *Potamocorbula* sp. as an indicator of clastic transport (Fig. S7). *Potamocorbula* sp. typically inhabits mud flats, and its thin juvenile shells occur mostly in the post-LGM incised valley fills in the study area^35^. Considered as a clastic constituent, these shells behave like fine-grained particles. The 45 cores yielded 37 reworked individuals of *Potamocorbula* sp. (Table S2). Among these specimens, the individual age offsets increased with increasing paleoelevations from –4 to –14 m, exceeded 400 years from –14 to –18 m paleoelevations, and were small again at paleoelevations smaller than –18 m (Fig. 4e). These shells originated in a tidal-flat environment, and delta-front sand gives way to prodelta mud at –12 to –13 m in the mouth of the pre-diversion Tone River^10^. Our interpretation of their age offsets and paleoelevations (Fig. 4e) is that clastics from the tidal flat were moved in stepwise fashion, mainly by hydraulic processes during storms, from the delta front to the prodelta. In the delta front, where the river provides a continuous supply of new clastics, coarse materials persist as a lag deposit after winnowing by storms whereas in the prodelta, mud winnowed by storms from the delta front settled from suspension. Larger storms, which have enough power to mix the delta-front sediments and generate suspensions capable of directly reaching the deeper part of the prodelta, are relatively rare. We infer, then, that relatively old and fine particles tended to be supplied to the deeper part of the prodelta. Delta-front sediments with mixed old and new clastics would be carried together into the deeper environments. In sum, the wide range of individual age offsets in fine-grained mud at paleoelevations of about –20 m can be explained by storm winnowing of fine-grained particles supplied from a river mouth, then stepwise transport by storms to deeper water environments.

Radiocarbon samples showing relatively small age offsets were from paleoelevations smaller than about –20 m (Fig. 4b, e). These samples, dated 7–4 ka, are all from facies SP in core GS-KNJ-1 and facies DF in cores GS-MHI-1 and GS-KBH-1, from the axis of the incised valley (Fig. 1c, Table S2). During the middle Holocene sea-level highstand, strong tidal currents occurred in the valley axis, and tidal troughs formed in the seafloor below the prodelta (Fig. S2c). Therefore, these samples appear to have been rapidly transported from the river mouth to the tidal troughs by tidal currents. Tsunamis have not been reported in Tokyo Bay during the historical epoch, because the bay is a semi-enclosed sea^36^. Therefore, it is unnecessary to consider tsunamis as a trigger of clastic transport in the subtidal environment.

Removal of clastics from the river mouth and delta front to the prodelta is confirmed by modern observations, and storms are considered to be the main cause^19,22^. Although deposition in prodeltas is mainly from settling of fine-grained particles from suspension, modern and ancient analogues have shown that flood-induced hyperpycnal flows or storm-induced gravity flows also influence deposition in prodeltas^37^. Bioturbation of sedimentary facies has made it difficult to reconstruct the hydraulic activity in the prodeltas of Tokyo Bay^10,38^. However, the results of this study indicate that settling from suspension is the predominant type of deposition in the prodeltas of Tokyo Bay, caused mainly by erosion and mixing of fine-grained particles by storms. We found that subtidal sediments had an average reworked percentage of ~ 40%, signifying that nearly 40% of clastics were successively redeposited by storms and nearly 60% of clastics were likely released from the river mouth by floods and directly transported to subtidal environments. The rate of flooding in Tokyo Bay may be high in comparison with other deltas because the sediment discharge of Japanese rivers is mainly controlled by floods during the rainy season or typhoons^25^. This is a particularly important finding, not only for source-to-sink studies, but also for quantitative analyses of strata formation in coastal areas.

## Methods

The 45 cores used in this study were obtained or analyzed by the Geological Survey of Japan (GSJ) during 2002–2020, and sedimentary facies and radiocarbon dates from the all cores have been reported in preliminary studies^10,28,39–41^ (Table S1). Most of the cores were obtained by percussion and rotary drilling methods, and the core recovery rates were almost 100%. The cores were split, photographed, and described in the GSJ laboratory. The lithology (grain size, color, texture, sedimentary structure, and character of contacts) and biofacies (composition and species of molluscan shells, burrows, and rootlets) were described from the split cores. Sand contents were measured every 20 cm by using a 63-µm sieve.

Radiocarbon dates from 757 samples of molluscan shell material, echinoderms, crab shells, gastropods, plants, wood fragments, and bulk sediment were measured by accelerator mass spectrometry at the National Institute for Environmental Studies, Japan^42^, the Japan Atomic Energy Agency^43^, the Institute of Accelerator Analysis Ltd., Japan, and Beta Analytic Inc., USA. Calibrated ^14^C ages were calculated by using CALIB 7.1 software^44^ and the IntCal13 and Marine13 datasets^45^. To calculate calibrated ^14^C ages for carbonate samples, ΔR was regarded as 0 yr because the ΔRs are different not only among the regions but also among the sedimentary environments^14,15^. Furthermore, ΔR values in coastal regions such as Tokyo Bay vary considerably^33^. The content of marine carbon was regarded as 100%.

In this study, we used the median probability of calibrated ^14^C ages (cal BP). Samples with younger ages than that of the underlying sample, or with the youngest age in the same horizon, were regarded as the depositional age and used to construct the sediment accumulation curve for each core (Fig. S1). Individual age offsets were determined as the interval between the reworked age and the sediment accumulation curve at the horizon of the sample. Paleoelevations were determined as the interval between the sediment accumulation curve and the sea-level curve at a given age (Fig. S1). Four plant samples (from –13.67 m in core GS-SK-1, –8.16 m in GS-KSO-1, –21.67 m in GS-TKT-1, and –24.50 m in GS-FB-2) were excluded when calculating the average age offsets because their individual age offsets were considered outliers (> 7,000 years; Table S2). Eleven shell samples (from –11.98 m in GS-NS-1, –10.07 m and –12.85 m in GS-CB-3, –22.78 m in GS-CB-4, –4.00 m in GS-CB-6, –14.29 m, –22.64 m, and –25.78 m in GS-CB-8, –15.74 m and –25.93 m in Hinode, and –32.29 m in Gyotoku) were not used to compare individual age offset and sand content because their sand contents were not measured (Table S2)^40^.

## Supplementary Information

Supplementary Information.
