# Supplementary material for: Recycling of clastics in coastal areas inferred from quantitative analysis of reworked radiocarbon samples
Source: Sci Rep. 2022 Jan 13;12:650. doi: 10.1038/s41598-021-04660-3 (PMC8758699; doi:10.1038/s41598-021-04660-3)
Supplement: Supplementary file 1 — Supplementary Information. [file 41598_2021_4660_MOESM1_ESM.pdf]

Supplementary information for

Recycling of clastics in coastal areas inferred from quantitative analysis of reworked radiocarbon samples

Susumu Tanabe[1], Toshimichi Nakanishi[2] & Rei Nakashima[1]

[1] Geological Survey of Japan, AIST, Central 7, Higashi 1-1-1, Tsukuba 305-8567, Japan
[2] Museum of Natural and Environmental History, Shizuoka, Oya 5762, Suruga-ku, Shizuoka 422-8017, Japan

This PDF file includes:

Figure captions and table titles
Figs. S1–7
Tables S1–6

Figure captions and table titles

Fig. S1. Sedimentary column and sediment accumulation curve of core GS-KNJ-1 (Fig. 1c). Sedimentary facies are as follows: SH, Shimosa Group; BR, braided-river sediments; MR, meandering-river sediments; TF, tidal-flat sediments; SP, spit sediments; DF, delta-front sediments; MF, modern fluvial sediments; AS, artificial soil. The sedimentary column is based on [10], and the sea-level curve in the area north of Tokyo Bay is after [27].

Fig. S2. Holocene paleogeography of the Tokyo and Nakagawa lowlands [10]. The Tone River shifted at 5 ka from the Arakawa Lowland to the Nakagawa Lowland (Fig. 1c).

Fig. S3. Frequency distributions of individual age offsets for samples of (blue) shell material and (red) plant material in facies DF and EF.

Fig. S4. Reworked percentage versus depositional age (500-yr intervals).

Fig. S5. Sediment accumulation curves of cores GS-KBH-1, GS-MHI-1, GS-SMB-1, and GS-TKT-1. The sea-level curve for the area north of Tokyo Bay is after [27]. MFS, maximum flooding surface.

Fig. S6. Depositional duration versus average age offset for each sedimentary facies.

Fig. S7. Juvenile valve of *Potamocorbula* sp. obtained from –21.88 m in core GS-SK-1 (Fig. 1c).

Table S1. Locations of sediment cores.

Table S2. Radiocarbon dates. Sedimentary facies are as follows: MR, meandering-river sediments; TF, tidal-flat sediments; EF, estuary-front sediments; SP, spit sediments; DF, delta-front sediments; MT, modern tidal-flat sediments; MF, modern fluvial sediments. Other abbreviations are as follows: DA, depositional age; RW, reworked age; Base, age

of the Shimosa Group; TERRA and NIES-TERRA, National Institute for Environmental Studies Japan; JNC, Japan Atomic Energy Agency; IAAA, Institute of Accelerator Analysis; Beta, Beta Analytic.

Table S3. Average age offsets of shells and plants in each sedimentary facies. The average age offset of facies MT was notably large because of a few reworked samples with large ages.

Table S4. Numbers of ages and reworked ages, reworked percentages, and average age offsets of sedimentary facies in 45 cores. Sedimentary facies are as follows: MR, meandering-river sediments; TF, tidal-flat sediments; EF, estuary-front sediments; SP, spit sediments; DF, delta-front sediments; MT, modern tidal-flat sediments; MF, modern fluvial sediments.

Table S5. Numbers of ages and reworked ages, average reworked percentages, and average individual age offsets of sedimentary facies and paleo-water depth categories. The average age offset of facies MT was notably large because of a few reworked samples with large ages.

Table S6. Depositional durations and average age offsets of sedimentary facies in 25 cores. Depositional durations were calculated for sedimentary facies for which at least two depositional ages calculated from reworked ages (Table S2) were available. The depositional durations were calculated by subtracting the youngest depositional age from the oldest depositional age. Sedimentary facies are as follows: MR, meandering-river sediments; TF, tidal-flat sediments; EF, estuary-front sediments; SP, spit sediments; DF, delta-front sediments; MT, modern tidal-flat sediments; MF, modern fluvial sediments.

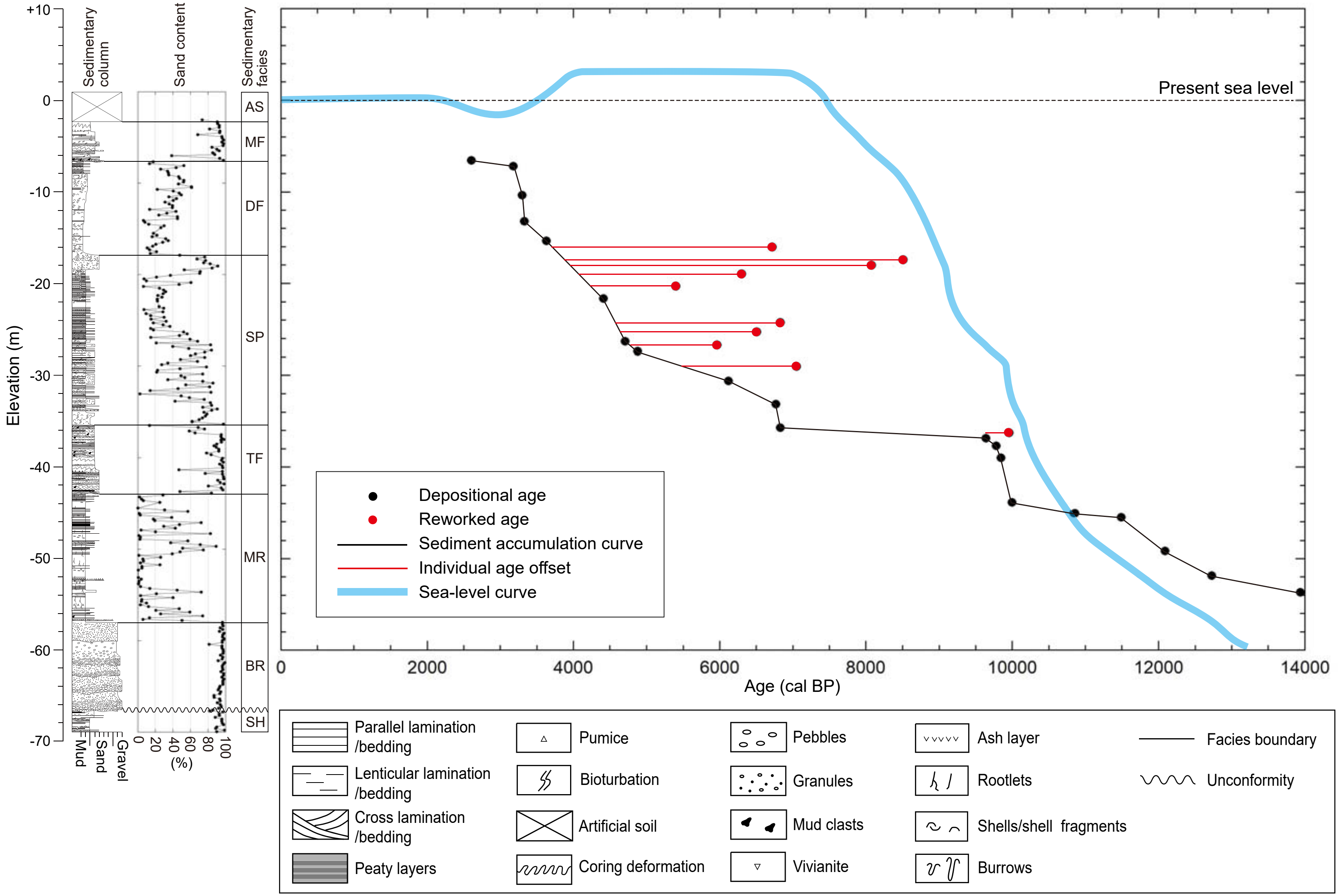

+10
0
-10
-20
-30
-40
-50
-60
-70
Elevation (m)
Sedimentary column
Sand content
Sedimentary facies
AS
MF
DF
SP
TF
MR
BR
SH
Mud
Sand
Gravel
0 20 40 60 80 100
(%)
Present sea level
0
2000
4000
6000
8000
10000
12000
14000
Age (cal BP)
Depositional age
Reworked age
Sediment accumulation curve
Individual age offset
Sea-level curve
Parallel lamination /bedding
Lenticular lamination /bedding
Cross lamination /bedding
Peaty layers
Pumice
Bioturbation
Artificial soil
Coring deformation
Pebbles
Granules
Mud clasts
Vivianite
Ash layer
Rootlets
Shells/shell fragments
Burrows
Facies boundary
Unconformity

Fig. S1

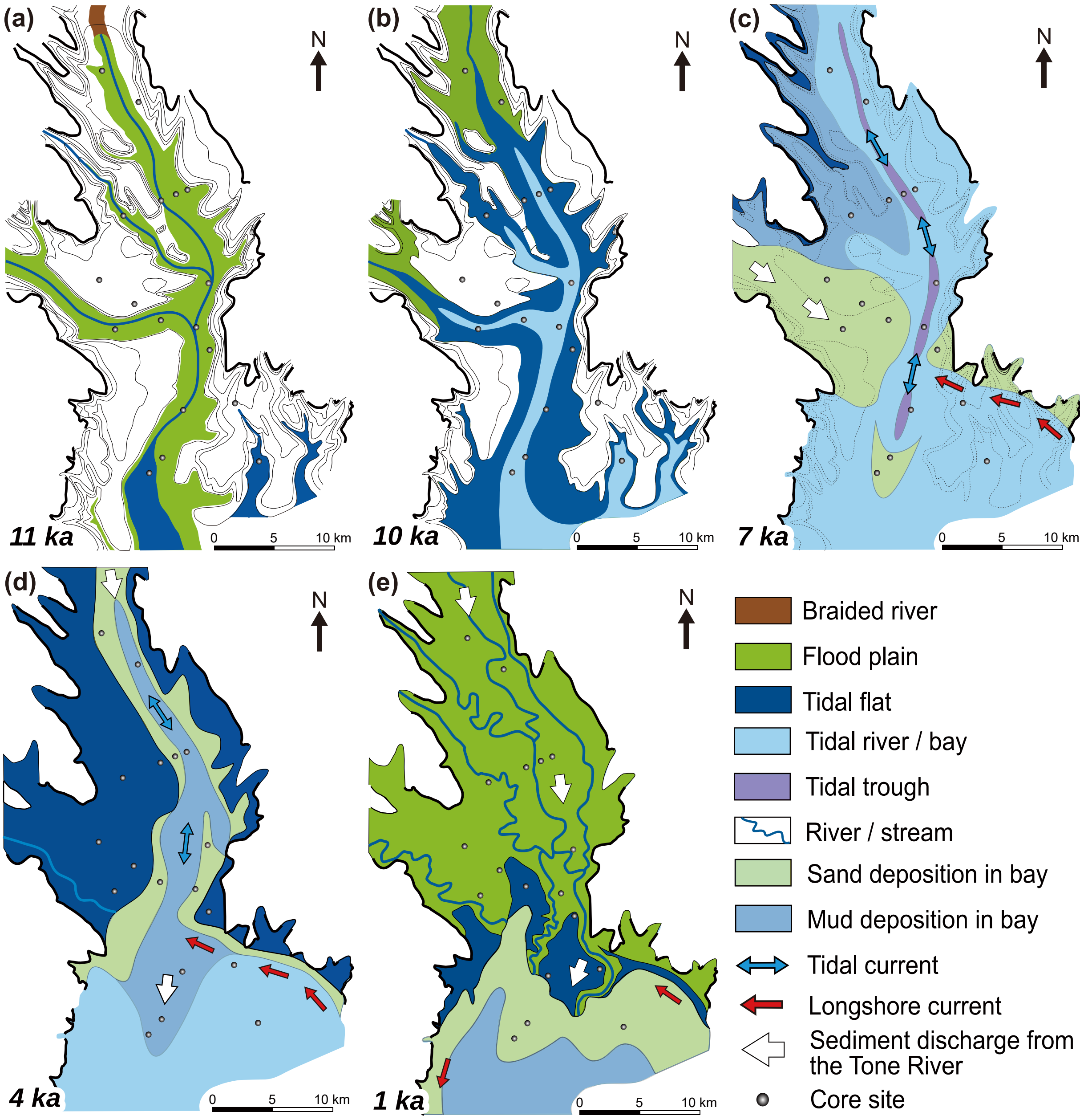

(a)
11 ka
(b)
10 ka
(c)
7 ka
(d)
4 ka
(e)
1 ka
N
0
5
10 km
Braided river
Flood plain
Tidal flat
Tidal river / bay
Tidal trough
River / stream
Sand deposition in bay
Mud deposition in bay
Tidal current
Longshore current
Sediment discharge from the Tone River
Core site

Fig. S2

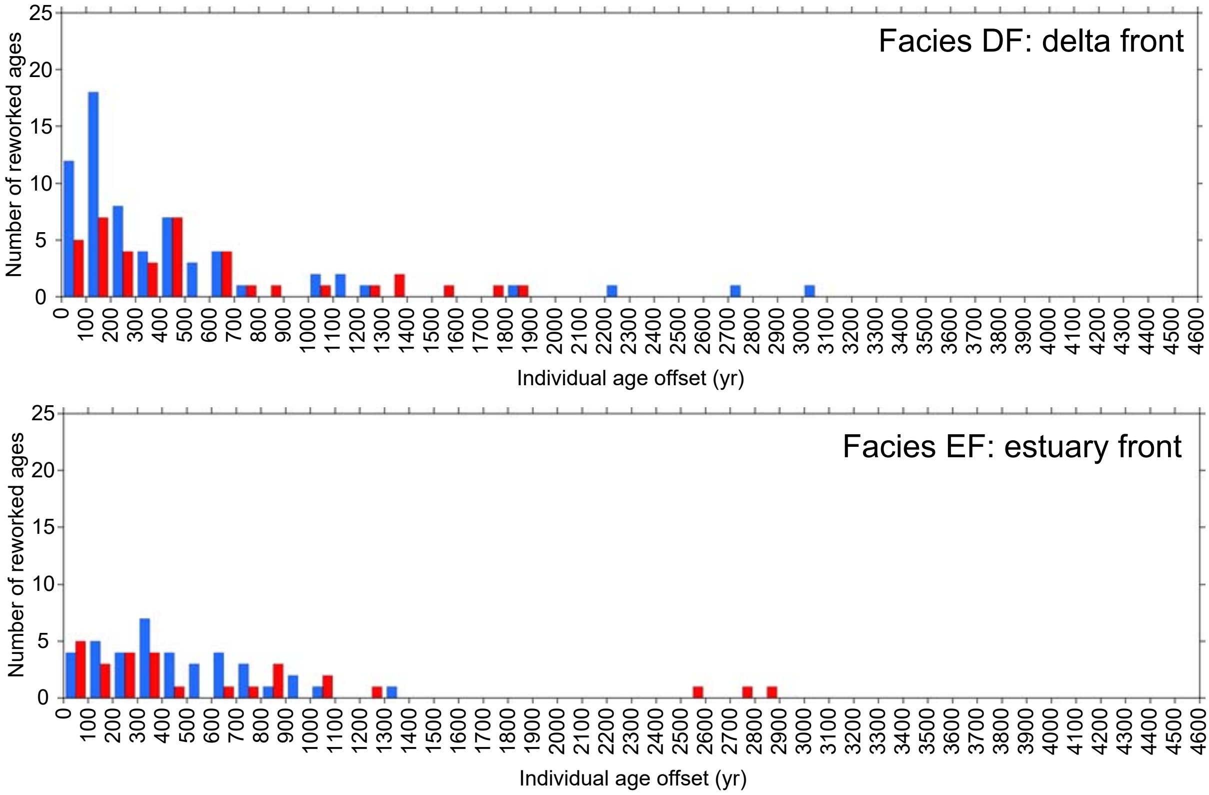
Facies DF: delta front
Number of reworked ages
Individual age offset (yr)
Facies EF: estuary front
Number of reworked ages
Individual age offset (yr)

Fig. S3

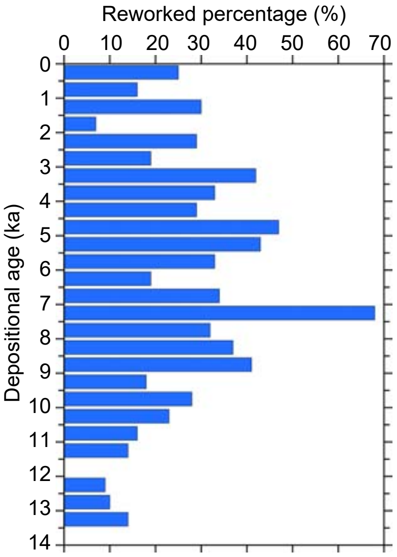

Reworked percentage (%)
0
10
20
30
40
50
60
70
Depositional age (ka)
0
1
2
3
4
5
6
7
8
9
10
11
12
13
14

Fig. S4

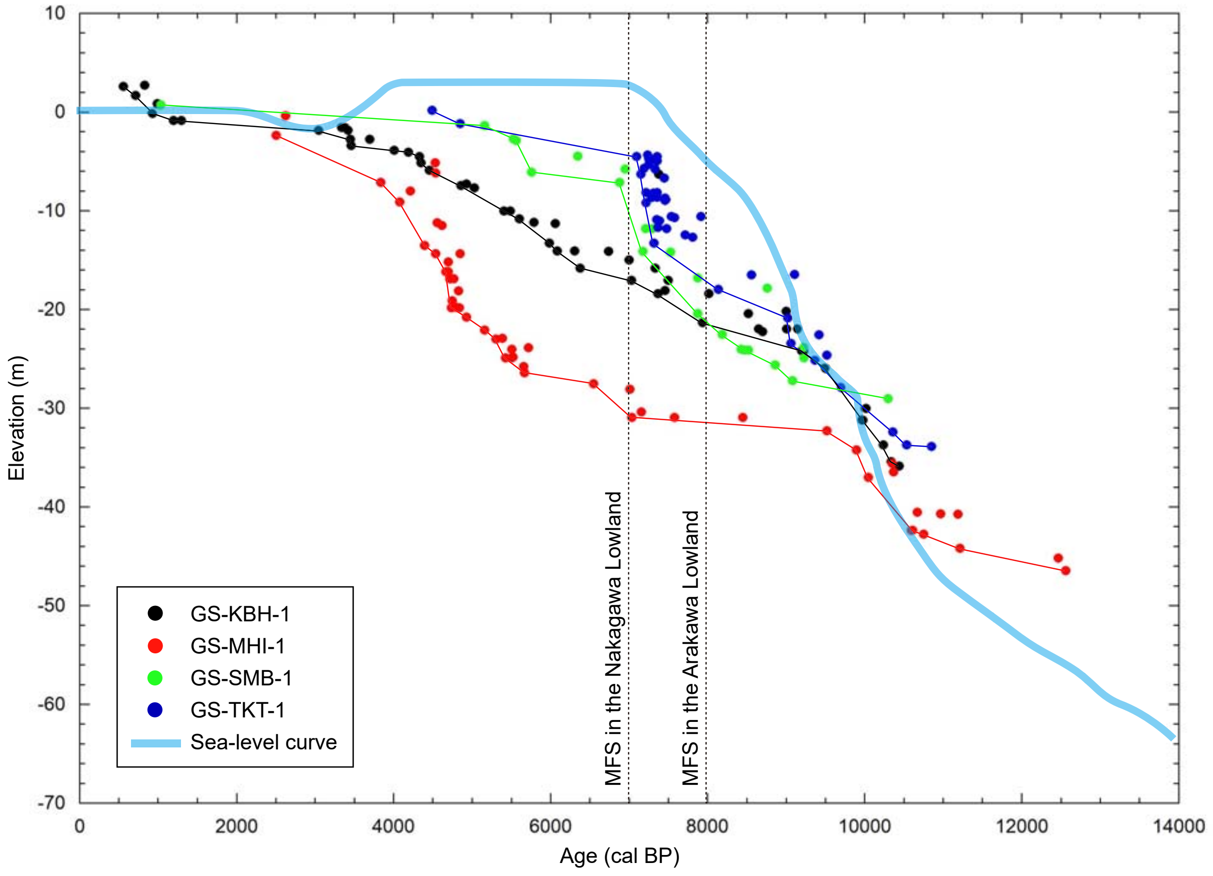

10
0
-10
-20
-30
-40
-50
-60
-70
Elevation (m)
0
2000
4000
6000
8000
10000
12000
14000
Age (cal BP)
MFS in the Nakagawa Lowland
MFS in the Arakawa Lowland
GS-KBH-1
GS-MHI-1
GS-SMB-1
GS-TKT-1
Sea-level curve

Fig. S5

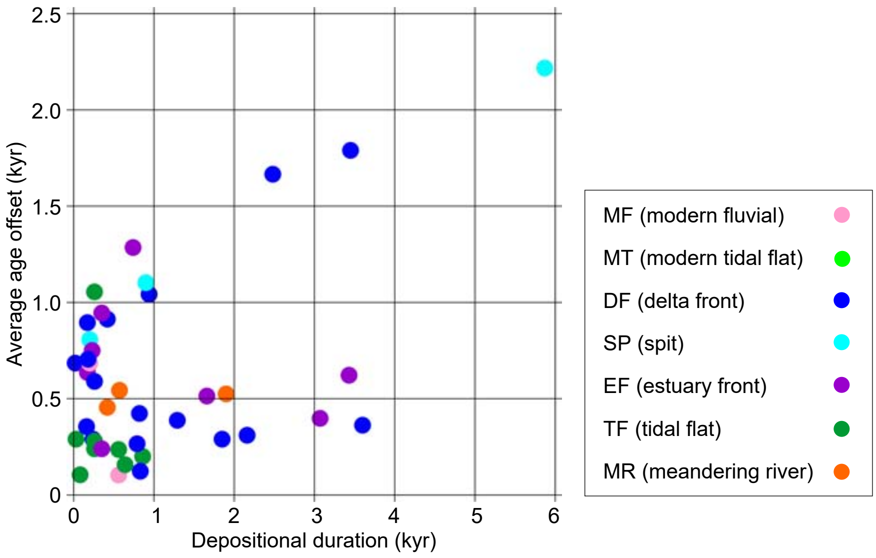
2.5
2.0
1.5
1.0
0.5
0
Average age offset (kyr)
0
1
2
3
4
5
6
Depositional duration (kyr)
MF (modern fluvial)
MT (modern tidal flat)
DF (delta front)
SP (spit)
EF (estuary front)
TF (tidal flat)
MR (meandering river)

Fig. S6

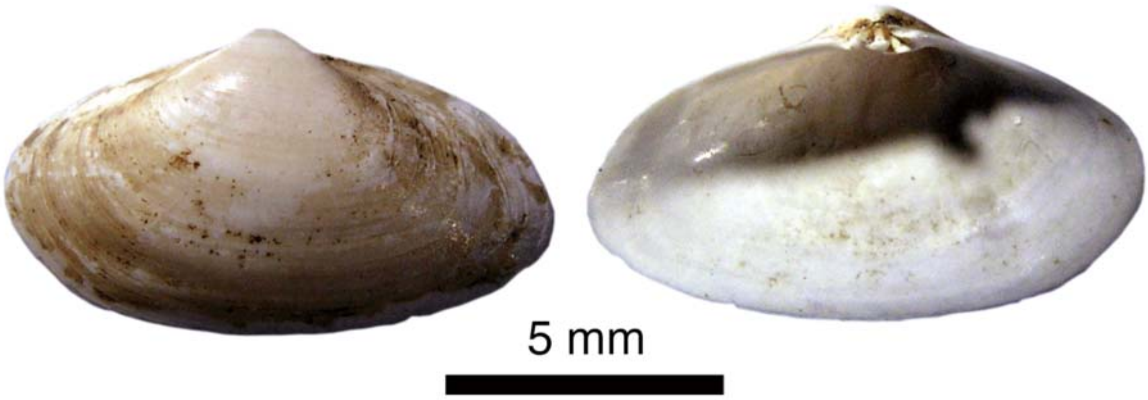

Fig. S7

Table S1

Core	Latitude (N)	Longitude (E)	Elevation (m)	Penetration depth (m)	Reference
GS-KBH-1	35°57′05.7″	139°46′25.6″	+5.36	49.90	Tanabe et al. (2015)
GS-KS-1	35°55′55.8″	139°48′20.1″	+5.34	57.00	Tanabe et al. (2015)
GS-MUS-1	35°51′51.3″	139°51′49.8″	+2.42	42.00	Tanabe et al. (2015)
GS-MHI-1	35°51′42.6″	139°51′05.6″	+3.41	55.00	Tanabe et al. (2015)
GS-SK-1	35°51′32.7″	139°50′06.9″	+3.73	60.00	Tanabe et al. (2015)
GS-SMB-1	35°50′45.6″	139°47′16.8″	+3.08	41.00	Tanabe et al. (2015)
TN	35°47′54.0″	139°46′17.4″	+2.88	40.00	Tanabe et al. (2015)
MZ	35°47′47.3″	139°52′24.3″	+1.90	60.00	Tanabe et al. (2015)
GS-AHH-1	35°47′04.0″	139°48′16.6″	+2.54	31.50	Tanabe et al. (2015)
HA	35°46′17.3″	139°49′48.7″	-0.03	70.00	Tanabe et al. (2015)
GS-KNJ-1	35°45′49.3″	139°51′38.8″	+0.43	70.00	Tanabe et al. (2015)
GS-AMG-1	35°45′43.2″	139°47′11.0″	+1.67	58.00	Tanabe et al. (2015)
GS-KTS-1	35°44′47.8″	139°52′23.2″	+1.19	42.00	Tanabe et al. (2015)
SZ	35°42′17.2″	139°53′50.1″	+0.61	13.00	Tanabe et al. (2015)
GS-KM-1	35°41′45.1″	139°50′57.4″	-1.99	67.23	Tanabe et al. (2015)
DK	35°39′59.1″	139°49′30.8″	+0.08	75.00	Tanabe et al. (2015)
GS-ISH-1	35°39′54.2″	139°54′52.8″	+2.69	60.00	Tanabe et al. (2015)
GS-KSM-1	35°39′18.6″	139°48′29.9″	+7.98	85.00	Tanabe et al. (2015)
GS-KWS-1	35°37′18.4″	139°50′0.1.1″	+8.55	84.92	Komatsubara et al. (2020)
GS-KSO-1	35°55′49.9″	139°31′46.0″	+9.77	40.00	Komatsubara et al. (2017)
GS-SSS-1	35°52′22.5″	139°34′45.0″	+4.63	45.50	Komatsubara et al. (2017)
GS-TKT-1	35°48′49.5″	139°40′40.7″	+3.12	51.00	Komatsubara et al. (2017)
GS-FB-2	35°40′19.4″	139°58′08.5″	+3.56	60.00	Kazaoka et al. (2018)
GS-FB-3	35°40′48.9″	139°59′09.7″	+3.48	110.00	Kazaoka et al. (2018)
GS-FB-4	35°40′07.7″	140°00′13.0″	+4.09	35.00	Kazaoka et al. (2018)
GS-NS-1	35°40′27.0″	140°01′01.6″	+2.90	30.00	Kazaoka et al. (2018)
GS-CB-2	35°38′59.8″	140°01′55.9″	+4.36	30.00	Kazaoka et al. (2018)
GS-CB-3	35°36′23.2″	140°06′21.4″	+5.38	40.00	Kazaoka et al. (2018)
GS-CB-4	35°38′32.7″	140°01′56.2″	+3.88	40.00	Kazaoka et al. (2018)
GS-CB-5	35°36′35.5″	140°04′17.1″	+4.30	110.00	Kazaoka et al. (2018)
GS-CB-6	35°36′08.3″	140°06′19.2″	+2.03	40.00	Kazaoka et al. (2018)
GS-CB-7	35°39′22.1″	140°03′47.0″	+4.39	30.00	Kazaoka et al. (2018)
GS-CB-8	35°37′37.3″	140°03′18.2″	+3.74	60.00	Kazaoka et al. (2018)
Hinode	35°41′23.4″	139°58′48.1″	+1.80	41.17	Kazaoka et al. (2018)
Gyotoku	35°39′55.1″	139°54′44.0″	+3.30	60.00	Kazaoka et al. (2018)
Hamada	35°39′20.1″	140°02′16.0″	+3.96	75.00	Kazaoka et al. (2018)
GS-KKW-1	35°31′04.0″	139°43′21.6″	+0.45	60.00	Tanabe et al. (2022)
GS-KSW-1	35°32′10.3″	139°41′18.9″	+2.58	36.00	Tanabe et al. (2022)
GS-KNH-1	35°33′31.1″	139°39′20.6″	+5.86	27.00	Tanabe et al. (2022)
GS-KNH-2	35°34′41.3″	139°38′26.2″	+9.43	24.00	Tanabe et al. (2022)
GS-KNH-3	35°35′21.3″	139°37′51.3″	+11.33	13.00	Tanabe et al. (2022)
GS-KNH-4	35°33′58.9″	139°39′28.5″	+6.8	19.00	Tanabe et al. (2022)
GS-YKH-1	35°32′08.7″	139°38′52.1″	+4.1	35.00	Tanabe et al. (2022)
GS-TOT-1	35°33′03.2″	139°44′10.2″	+1.88	10.00	Tanabe et al. (2022)
GS-TOT-2	35°33′41.0″	139°42′38.8″	+3.06	10.00	Tanabe et al. (2022)

Table S2

Core	Depth in core (m)	Elevation (m)	Material	Species	Conventional ^{14}C age (BP)	Calibrated ^{14}C age (2σ range) (cal BP)	Median probability (cal BP)	Sedimentary facies	Individual age offset	(yr)	Depositional age (cal BP)	Paleoelevation (m)	Sand content (%)	Lab code	Reference
GS-KBH-1	2.63	2.73	Plant		910 ± 60	710–930	830	MF	RW	150	680	3	11	NIES-TERAA-b011306a10	Tanabe et al. (2015)
GS-KBH-1	2.76	2.60	Plant		550 ± 30	520–640	560	MF	DA	———	———	———	———	NIES-TERAA-b081205a32	Tanabe et al. (2015)
GS-KBH-1	3.70	1.66	Plant		810 ± 40	670–790	720	MF	DA	———	———	———	———	NIES-TERAA-b081205a32	Tanabe et al. (2015)
GS-KBH-1	4.52	0.84	Plant		1080 ± 40	930–1060	990	MF	RW	100	890	1	22	NIES-TERAA-b081205a33	Tanabe et al. (2015)
GS-KBH-1	5.50	-0.14	Plant		1010 ± 40	800–1050	930	MF	DA	———	———	———	———	NIES-TERAA-b081205a35	Tanabe et al. (2015)
GS-KBH-1	6.24	-0.88	Plant		1260 ± 50	1070–1290	1200	MF	DA	———	———	———	———	NIES-TERAA-b081205a36	Tanabe et al. (2015)
GS-KBH-1	6.24	-0.88	Plant		1370 ± 40	1190–1350	1300	MF	RW	60	1240	-1	34	NIES-TERAA-b011306a13	Tanabe et al. (2015)
GS-KBH-1	6.93	-1.57	Plant		3130 ± 70	3160–3550	3340	DF	RW	210	3130	0	1	NIES-TERAA-b081205a37	Tanabe et al. (2015)
GS-KBH-1	6.93	-1.57	Plant		3160 ± 50	3240–3480	3380	DF	RW	210	3170	0	1	NIES-TERAA-b011306a14	Tanabe et al. (2015)
GS-KBH-1	7.21	-1.85	Plant		2910 ± 50	2890–3210	3050	DF	DA	———	———	———	———	NIES-TERAA-b081205a33	Tanabe et al. (2015)
GS-KBH-1	7.21	-1.85	Plant		3190 ± 40	3270–3550	3420	DF	RW	210	3210	0	1	NIES-TERAA-b011306a15	Tanabe et al. (2015)
GS-KBH-1	8.11	-2.75	Plant		3230 ± 40	3380–3560	3450	DF	DA	———	———	———	———	NIES-TERAA-b081205a38	Tanabe et al. (2015)
GS-KBH-1	8.11	-2.75	Plant		3440 ± 40	3600–3830	3700	DF	RW	170	3530	-3	2	NIES-TERAA-b011306a16	Tanabe et al. (2015)
GS-KBH-1	8.76	-3.40	Plant		3230 ± 100	3210–3690	3460	DF	DA	———	———	———	———	NIES-TERAA-b082605a03	Tanabe et al. (2015)
GS-KBH-1	9.23	-3.87	Shell	*Potamocorbula* sp.	4000 ± 40	3880–4140	4010	DF	DA	———	———	———	———	NIES-TERAA-b080505a16	Tanabe et al. (2015)
GS-KBH-1	9.45	-4.09	Plant		3800 ± 40	4010–4400	4190	DF	DA	———	———	———	———	NIES-TERAA-b011306a17	Tanabe et al. (2015)
GS-KBH-1	9.88	-4.52	Shell	*Potamocorbula* sp.	4230 ± 50	4170–4480	4330	DF	RW	40	4290	-8	4	NIES-TERAA-b080505a19	Tanabe et al. (2015)
GS-KBH-1	10.51	-5.15	Shell	*Potamocorbula* sp.	4250 ± 70	4140–4540	4350	DF	DA	———	———	———	———	NIES-TERAA-b080505a20	Tanabe et al. (2015)
GS-KBH-1	11.25	-5.89	Shell	*Potamocorbula* sp.	4330 ± 40	4350–4590	4460	DF	DA	———	———	———	———	NIES-TERAA-b082605a04	Tanabe et al. (2015)
GS-KBH-1	11.65	-6.29	Plant		6480 ± 50	7280–7480	7380	DF	RW	1770	5610	-9	10	NIES-TERAA-b011306a18	Tanabe et al. (2015)
GS-KBH-1	12.66	-7.30	Shell	*Potamocorbula* sp.	4700 ± 50	4810–5110	4930	DF	RW	40	4890	-10	25	NIES-TERAA-b080505a21	Tanabe et al. (2015)
GS-KBH-1	12.80	-7.44	Plant		4290 ± 40	4730–4970	4860	DF	DA	———	———	———	———	NIES-TERAA-b011306a19	Tanabe et al. (2015)
GS-KBH-1	13.05	-7.69	Shell	*Potamocorbula* sp.	4770 ± 40	4880–5210	5030	DF	DA	———	———	———	———	NIES-TERAA-b080505a22	Tanabe et al. (2015)
GS-KBH-1	15.37	-10.01	Shell	*Raetellops pulchellus* (Adams et Reeve)	5120 ± 40	5330–5580	5490	DF	RW	40	5450	-13	9	NIES-TERAA-b080505a24	Tanabe et al. (2015)
GS-KBH-1	15.37	-10.01	Shell	*Potamocorbula* sp.	5060 ± 40	5300–5530	5410	DF	DA	———	———	———	———	NIES-TERAA-b080505a25	Tanabe et al. (2015)
GS-KBH-1	16.17	-10.81	Shell	*Ringiculina doliaris* (Gould)	5240 ± 50	5480–5710	5600	DF	DA	———	———	———	———	NIES-TERAA-b080505a26	Tanabe et al. (2015)
GS-KBH-1	16.52	-11.16	Plant		5030 ± 50	5660–5900	5790	DF	DA	———	———	———	———	NIES-TERAA-b011306a20	Tanabe et al. (2015)
GS-KBH-1	16.63	-11.27	Plant		5270 ± 50	5930–6180	6060	DF	RW	150	5910	-14	13	NIES-TERAA-b082605a06	Tanabe et al. (2015)
GS-KBH-1	18.66	-13.30	Shell	*Ringiculina doliaris* (Gould)	5600 ± 40	5890–6120	5990	DF	DA	———	———	———	———	NIES-TERAA-b080505a27	Tanabe et al. (2015)
GS-KBH-1	19.44	-14.08	Shell	*Raetellops pulchellus* (Adams et Reeve)	5890 ± 40	6220–6400	6310	DF	RW	130	6180	-17	17	NIES-TERAA-b080505a29	Tanabe et al. (2015)
GS-KBH-1	19.44	-14.08	Shell	*Potamocorbula* sp.	5680 ± 50	5940–6210	6090	DF	DA	———	———	———	———	NIES-TERAA-b080505a30	Tanabe et al. (2015)
GS-KBH-1	19.47	-14.11	Plant		5920 ± 30	6670–6830	6740	DF	RW	420	6320	-17	17	NIES-TERAA-b081205a16	Tanabe et al. (2015)
GS-KBH-1	20.33	-14.97	Shell	*Potamocorbula* sp.	6490 ± 50	6860–7140	7000	DF	RW	480	6520	-18	12	NIES-TERAA-b080505a31	Tanabe et al. (2015)
GS-KBH-1	21.20	-15.84	Shell	*Ringiculina doliaris* (Gould)	6800 ± 40	7250–7410	7330	DF	RW	600	6730	-19	8	NIES-TERAA-b082605a04	Tanabe et al. (2015)
GS-KBH-1	21.20	-15.84	Shell	*Potamocorbula* sp.	6800 ± 40	7250–7410	7330	DF	RW	600	6730	-19	8	NIES-TERAA-b080505a32	Tanabe et al. (2015)
GS-KBH-1	21.20	-15.84	Plant		5600 ± 50	6300–6480	6380	DF	DA	———	———	———	———	NIES-TERAA-b081205a19	Tanabe et al. (2015)
GS-KBH-1	22.39	-17.03	Shell	*Theora fragilis* (Adams)	7000 ± 40	7420–7560	7500	EF	RW	460	7040	-20	9	NIES-TERAA-b080505a33	Tanabe et al. (2015)
GS-KBH-1	22.39	-17.03	Plant		6130 ± 40	6910–7160	7030	EF	DA	———	———	———	———	NIES-TERAA-b081205a20	Tanabe et al. (2015)
GS-KBH-1	23.45	-18.09	Plant		6540 ± 50	7330–7560	7460	EF	RW	130	7330	-19	15	NIES-TERAA-b081205a39	Tanabe et al. (2015)
GS-KBH-1	23.75	-18.39	Shell	*Ringiculina doliaris* (Gould)	7560 ± 50	7920–8140	8020	EF	RW	630	7390	-19	27	NIES-TERAA-b080505a35	Tanabe et al. (2015)
GS-KBH-1	23.75	-18.39	Plant		6460 ± 50	7270–7460	7370	EF	DA	———	———	———	———	NIES-TERAA-b081205a22	Tanabe et al. (2015)
GS-KBH-1	25.55	-20.19	Plant		8070 ± 50	8770–9130	9000	EF	RW	1290	7710	-18	5	NIES-TERAA-b011306a23	Tanabe et al. (2015)
GS-KBH-1	25.80	-20.44	Shell	*Potamocorbula* sp.	8070 ± 50	8400–8650	8520	EF	RW	770	7750	-17	12	NIES-TERAA-b080505a36	Tanabe et al. (2015)
GS-KBH-1	26.68	-21.32	Plant		7110 ± 50	7840–8020	7940	EF	DA	———	———	———	———	NIES-TERAA-b081205a24	Tanabe et al. (2015)
GS-KBH-1	27.32	-21.96	Shell	*Potamocorbula* sp.	8410 ± 60	8790–9210	9010	EF	RW	330	8680	-12	17	NIES-TERAA-b080505a38	Tanabe et al. (2015)
GS-KBH-1	27.32	-21.96	Shell	*Crassostrea* sp.	8520 ± 60	8990–9320	9150	EF	RW	500	8650	-12	17	NIES-TERAA-b080505a39	Tanabe et al. (2015)
GS-KBH-1	27.32	-21.96	Plant		7860 ± 40	8550–8930	8650	EF	DA	———	———	———	———	NIES-TERAA-b081205a25	Tanabe et al. (2015)
GS-KBH-1	27.61	-22.25	Plant		7880 ± 50	8560–8980	8700	EF	RW	0	8700	-12	1	NIES-TERAA-b081205a3	Tanabe et al. (2015)
GS-KBH-1	29.55	-24.19	Plant		8230 ± 50	9030–9400	9200	TF	DA	———	———	———	———	NIES-TERAA-b010706a31	Tanabe et al. (2015)
GS-KBH-1	29.55	-24.19	Plant		8250 ± 50	9030–9410	9230	TF	RW	20	9210	0	5	NIES-TERAA-b011306a24	Tanabe et al. (2015)
GS-KBH-1	31.35	-25.99	Plant		8500 ± 40	9460–9540	9510	TF	DA	———	———	———	———	Beta-209336	Tanabe et al. (2015)
GS-KBH-1	35.38	-30.02	Plant		8920 ± 90	9710–10230	10020	MR	RW	150	9870	-1	37	NIES-TERAA-b081205a9	Tanabe et al. (2015)
GS-KBH-1	36.59	-31.23	Plant		8860 ± 60	9710–10180	9980	MR	DA	———	———	———	———	NIES-TERAA-b011306a25	Tanabe et al. (2015)
GS-KBH-1	39.05	-33.69	Plant		9080 ± 60	10160–10480	10240	MR	DA	———	———	———	———	NIES-TERAA-b081205a14	Tanabe et al. (2015)
GS-KBH-1	40.77	-35.41	Plant		9180 ± 50	10240–10490	10340	MR	DA	———	———	———	———	NIES-TERAA-b081205a39	Tanabe et al. (2015)
GS-KBH-1	41.23	-35.87	Plant		9260 ± 60	10260–10580	10440	MR	DA	———	———	———	———	NIES-TERAA-b011306a27	Tanabe et al. (2015)
GS-KS-1	7.15	-1.81	Plant		3320 ± 60	3400–3690	3550	MF	DA	———	———	———	———	NIES-TERRA-b010505a13	Tanabe et al. (2015)
GS-KS-1	14.00	-8.66	Shell	*Cadella delta* (Yokoyama)	5620 ± 50	5910–6160	6020	DF	RW	1050	4970	-12	96	NIES-TERRA-b122504a20	Tanabe et al. (2015)
GS-KS-1	15.13	-9.79	Shell	*Potamocorbula* sp.	4880 ± 50	5020–5310	5190	DF	DA	———	———	———	———	NIES-TERRA-b122504a21	Tanabe et al. (2015)
GS-KS-1	17.00	-11.66	Shell	*Cadella delta* (Yokoyama)	6190 ± 50	6490–6750	6630	DF	RW	1050	5580	-15	90	NIES-TERRA-b122504a22	Tanabe et al. (2015)
GS-KS-1	19.84	-14.50	Shell	*Potamocorbula* sp.	8600 ± 50	9100–9400	9260	DF	RW	3050	6210	-18	57	NIES-TERRA-b122504a23	Tanabe et al. (2015)
GS-KS-1	21.65	-16.31	Shell	*Raetellops pulchellus* (Adams et Reeve)	8020 ± 70	8340–8630	8480	DF	RW	1840	6640	-19	57	NIES-TERRA-b122504a26	Tanabe et al. (2015)
GS-KS-1	21.65	-16.31	Plant		7400 ± 60	8050–8360	8240	DF	RW	1580	6660	-19	57	NIES-TERRA-b122004a21	Tanabe et al. (2015)
GS-KS-1	22.59	-17.25	Shell	*Veremolpa micra* (Pilsbry)	6200 ± 40	6520–6750	6640	DF	DA	———	———	———	———	NIES-TERRA-b122504a27	Tanabe et al. (2015)
GS-KS-1	22.59	-17.25	Shell	*Crassostrea* sp.	6830 ± 50	7250–7440	7350	DF	RW	630	6720	-20	22	NIES-TERRA-b122504a31	Tanabe et al. (2015)
GS-KS-1	22.59	-17.25	Echinoderm		7480 ± 60	7810–8090	7940	DF	RW	1260	6680	-20	22	NIES-TERRA-b122504a32	Tanabe et al. (2015)
GS-KS-1	25.58	-20.24	Plant		8030 ± 100	8600–9240	8890	DF	RW	1370	7520	-20	11	NIES-TERRA-b010505a16	Tanabe et al. (2015)
GS-KS-1	26.40	-21.06	Plant		9380 ± 70	10300–11060	10610	DF	RW	3160	7450	-21	21	NIES-TERRA-b122004a28	Tanabe et al. (2015)
GS-KS-1	30.76	-25.42	Plant		8450 ± 60	9310–9540	9470	EF	RW	1050	8420	-17	60	NIES-TERRA-b010505a19	Tanabe et al. (2015)
GS-KS-1	31.45	-26.11	Shell	*Potamocorbula* sp.	8030 ± 50	8380–8590	8480	EF	DA	———	———	———	———	NIES-TERRA-b122504a29	Tanabe et al. (2015)
GS-KS-1	35.74	-30.40	Plant		8820 ± 80	9610–10170	9880	MR	DA	———	———	———	———	NIES-TERRA-b010505a20	Tanabe et al. (2015)
GS-KS-1	39.87	-34.53	Plant		8860 ± 60	9710–10180	9980	MR	DA	———	———	———	———	NIES-TERRA-b010505a21	Tanabe et al. (2015)
GS-KS-1	43.54	-38.20	Plant		9650 ± 60	10780–11200	11000	MR	DA	———	———	———	———	NIES-TERRA-b010505a22	Tanabe et al. (2015)

GS-MUS-1	1.60	0.82	Plant		2470 ± 80	2360–2730	2550	MF	DA	———	———	———	———	TERRA-012109a03	Tanabe et al. (2015)
GS-MUS-1	3.35	-0.93	Plant		2700 ± 30	2760–2850	2800	MF	DA	———	———	———	———	TERRA-012109a04	Tanabe et al. (2015)
GS-MUS-1	5.45	-3.03	Plant		3360 ± 30	3490–3690	3600	MF	RW	320	3280	-2	12	TERRA-012109a05	Tanabe et al. (2015)
GS-MUS-1	6.85	-4.43	Shell	*Potamocorbula* sp.	3660 ± 30	3470–3670	3570	DF	RW	60	3510	-4	10	TERRA-011909a03	Tanabe et al. (2015)
GS-MUS-1	6.85	-4.43	Plant		3280 ± 30	3450–3580	3510	DF	DA	———	———	———	———	TERRA-012109a06	Tanabe et al. (2015)
GS-MUS-1	8.15	-5.73	Shell	*Potamocorbula* sp.	3680 ± 30	3490–3690	3590	DF	DA	———	———	———	———	TERRA-011909a04	Tanabe et al. (2015)
GS-MUS-1	14.65	-12.23	Shell	Shell fragments	3730 ± 40	3550–3800	3650	DF	RW	50	3600	-13	70	Beta-226998	Tanabe et al. (2015)
GS-MUS-1	16.70	-14.28	Shell	Shell fragments	3720 ± 40	3540–3790	3640	DF	DA	———	———	———	———	Beta-226999	Tanabe et al. (2015)
GS-MUS-1	17.35	-14.93	Plant		4410 ± 30	4870–5260	4980	DF	RW	1320	3660	-16	10	TERRA-012109a08	Tanabe et al. (2015)
GS-MUS-1	19.70	-17.28	Shell	Shell fragments	3890 ± 40	3720–3980	3860	DF	DA	———	———	———	———	Beta-227000	Tanabe et al. (2015)
GS-MUS-1	20.55	-18.13	Shell	*Potamocorbula* sp.	4060 ± 40	3960–4230	4090	DF	DA	———	———	———	———	TERRA-011909a05	Tanabe et al. (2015)
GS-MUS-1	20.60	-18.18	Plant		3930 ± 30	4250–4500	4370	DF	DA	———	———	———	———	TERRA-012109a09	Tanabe et al. (2015)
GS-MUS-1	21.29	-18.87	Shell	*Ringiculina doliaris* (Gould)	6660 ± 60	7020–7320	7190	DF	RW	2740	4450	-22	98	TERRA-011909a06	Tanabe et al. (2015)
GS-MUS-1	22.07	-19.65	Shell	*Ringiculina doliaris* (Gould)	4790 ± 70	4870–5270	5070	DF	DA	———	———	———	———	TERRA-012609a03	Tanabe et al. (2015)
GS-MUS-1	23.38	-20.96	Shell	*Ringiculina doliaris* (Gould)	5250 ± 30	5560–5690	5610	DF	DA	———	———	———	———	TERRA-011909a08	Tanabe et al. (2015)
GS-MUS-1	24.25	-21.83	Shell	Shell fragments	5710 ± 40	5990–6230	6120	DF	DA	———	———	———	———	Beta-227001	Tanabe et al. (2015)
GS-MUS-1	25.50	-23.08	Shell	*Ringiculina doliaris* (Gould)	6870 ± 180	6980–7700	7370	EF	DA	———	———	———	———	TERRA-011909a09	Tanabe et al. (2015)
GS-MUS-1	26.10	-23.68	Shell	Shell fragments	7390 ± 40	7760–7940	7860	EF	DA	———	———	———	———	Beta-227002	Tanabe et al. (2015)
GS-MUS-1	26.60	-24.18	Shell	*Crassostrea* sp.	7460 ± 40	7830–8000	7920	EF	DA	———	———	———	———	TERRA-011909a10	Tanabe et al. (2015)
GS-MUS-1	27.55	-25.13	Wormsnail		7620 ± 30	7990–8160	8080	EF	DA	———	———	———	———	TERRA-011909a11	Tanabe et al. (2015)
GS-MUS-1	28.70	-26.28	Shell	*Crassostrea* sp.	7840 ± 40	8200–8380	8310	EF	DA	———	———	———	———	TERRA-011909a12	Tanabe et al. (2015)
GS-MUS-1	30.40	-27.98	Shell	*Crassostrea* sp.	7980 ± 60	8330–8570	8440	EF	DA	———	———	———	———	TERRA-011909a15	Tanabe et al. (2015)
GS-MUS-1	31.05	-28.63	Shell	*Crassostrea* sp.	8230 ± 70	8570–8970	8760	EF	DA	———	———	———	———	TERRA-011909a16	Tanabe et al. (2015)
GS-MUS-1	34.22	-31.80	Plant		32840 ± 130	———	———	SH	Base	———	———	———	———	TERRA-012109a10	Tanabe et al. (2015)
GS-MUS-1	39.30	-36.88	Plant		29690 ± 120	———	———	SH	Base	———	———	———	———	TERRA-012109a11	Tanabe et al. (2015)
GS-MHI-1	3.77	-0.36	Plant		2550 ± 40	2490–2760	2630	MF	RW	340	2290	0	75	NIES-TERRA-b083104a03	Tanabe et al. (2015)
GS-MHI-1	5.78	-2.37	Plant		2440 ± 50	2360–2710	2510	MF	DA	———	———	———	———	NIES-TERRA-b122004a29	Tanabe et al. (2015)
GS-MHI-1	8.54	-5.13	Plant		4050 ± 40	4420–4800	4530	DF	RW	1290	3240	-4	99	NIES-TERRA-b083104a04	Tanabe et al. (2015)
GS-MHI-1	9.57	-6.16	Plant		4040 ± 90	4290–4830	4540	DF	RW	1090	3450	-6	19	NIES-TERRA-b083104a05	Tanabe et al. (2015)
GS-MHI-1	10.54	-7.13	Shell	Shell fragments	3870 ± 60	3650–4000	3840	DF	DA	———	———	———	———	NIES-TERRA-b090404a03	Tanabe et al. (2015)
GS-MHI-1	11.38	-7.97	Shell	Shell fragments	4150 ± 90	3960–4470	4220	DF	RW	400	3820	-10	34	NIES-TERRA-b083104a06	Tanabe et al. (2015)
GS-MHI-1	12.54	-9.13	Shell	Shell fragments	4050 ± 60	3890–4260	4080	DF	DA	———	———	———	———	NIES-TERRA-b083104a07	Tanabe et al. (2015)
GS-MHI-1	14.64	-11.23	Plant		4060 ± 50	4420–4810	4560	DF	RW	430	4130	-14	26	NIES-TERRA-b090404a04	Tanabe et al. (2015)
GS-MHI-1	14.90	-11.49	Plant		4100 ± 40	4450–4820	4620	DF	RW	490	4130	-14	31	NIES-TERRA-b083104a09	Tanabe et al. (2015)
GS-MHI-1	16.92	-13.51	Plant		3950 ± 50	4250–4520	4400	DF	DA	———	———	———	———	NIES-TERRA-b083104a10	Tanabe et al. (2015)
GS-MHI-1	17.78	-14.37	Plant		4050 ± 50	4420–4810	4540	DF	DA	———	———	———	———	NIES-TERRA-b083104a11	Tanabe et al. (2015)
GS-MHI-1	17.78	-14.37	Shell	Shell fragments	4630 ± 50	4710–5000	4850	DF	RW	430	4420	-17	11	NIES-TERRA-b082604a03	Tanabe et al. (2015)
GS-MHI-1	18.60	-15.19	Plant		4170 ± 60	4530–4840	4700	DF	RW	140	4560	-18	19	NIES-TERRA-b083104a12	Tanabe et al. (2015)
GS-MHI-1	19.57	-16.16	Plant		4140 ± 70	4450–4840	4670	DF	DA	———	———	———	———	NIES-TERRA-b083104a13	Tanabe et al. (2015)
GS-MHI-1	19.57	-16.16	Shell	*Potamocorbula* sp.	4500 ± 50	4540–4820	4700	DF	RW	60	4640	-19	18	NIES-TERRA-b082604a04	Tanabe et al. (2015)
GS-MHI-1	20.32	-16.91	Plant		4190 ± 60	4540–4850	4720	DF	DA	———	———	———	———	NIES-TERRA-b082604a35	Tanabe et al. (2015)
GS-MHI-1	20.32	-16.91	Shell	*Potamocorbula* sp.	4560 ± 50	4590–4880	4770	DF	RW	60	4710	-20	6	NIES-TERRA-b082604a05	Tanabe et al. (2015)
GS-MHI-1	21.52	-18.11	Shell	*Potamocorbula* sp.	4610 ± 50	4680–4970	4830	DF	RW	140	4690	-21	16	NIES-TERRA-b082604a07	Tanabe et al. (2015)
GS-MHI-1	22.52	-19.11	Plant		4240 ± 70	4570–4970	4750	DF	RW	10	4740	-22	19	NIES-TERRA-b083104a17	Tanabe et al. (2015)
GS-MHI-1	23.20	-19.79	Shell	*Potamocorbula* sp.	4580 ± 50	4620–4920	4800	DF	RW	140	4660	-23	7	NIES-TERRA-b082604a08	Tanabe et al. (2015)
GS-MHI-1	23.24	-19.83	Plant		4230 ± 70	4530–4960	4740	DF	DA	———	———	———	———	NIES-TERRA-b083104a18	Tanabe et al. (2015)
GS-MHI-1	23.24	-19.83	Shell	Shell fragments	4620 ± 60	4670–5020	4840	DF	RW	140	4700	-23	7	NIES-TERRA-b082604a27	Tanabe et al. (2015)
GS-MHI-1	24.20	-20.79	Shell	Shell fragments	4700 ± 50	4810–5110	4930	DF	DA	———	———	———	———	NIES-TERRA-b082604a09	Tanabe et al. (2015)
GS-MHI-1	25.50	-22.09	Shell	*Ringiculina doliaris* (Gould)	4860 ± 50	5000–5300	5160	DF	DA	———	———	———	———	NIES-TERRA-b082604a10	Tanabe et al. (2015)
GS-MHI-1	26.33	-22.92	Shell	*Macoma* cf. *tokyoensis* Makiyama	5040 ± 50	5280–5530	5390	DF	RW	90	5300	-26	3	NIES-TERRA-b082604a13	Tanabe et al. (2015)
GS-MHI-1	26.42	-23.01	Shell	*Ringiculina doliaris* (Gould)	4960 ± 50	5130–5450	5310	DF	DA	———	———	———	———	NIES-TERRA-b082604a14	Tanabe et al. (2015)
GS-MHI-1	27.29	-23.88	Plant		4980 ± 70	5600–5890	5720	DF	RW	490	5230	-27	5	NIES-TERRA-b083104a19	Tanabe et al. (2015)
GS-MHI-1	27.42	-24.01	Shell	Shell fragments	5150 ± 50	5350–5620	5510	DF	RW	170	5340	-27	5	NIES-TERRA-b082604a28	Tanabe et al. (2015)
GS-MHI-1	28.22	-24.81	Shell	*Ringiculina doliaris* (Gould)	5160 ± 70	5320–5650	5520	DF	RW	110	5410	-28	9	NIES-TERRA-b082604a29	Tanabe et al. (2015)
GS-MHI-1	28.25	-24.84	Shell	*Ringiculina doliaris* (Gould)	5160 ± 50	5410–5640	5520	DF	RW	110	5410	-28	9	NIES-TERRA-b082604a30	Tanabe et al. (2015)
GS-MHI-1	28.33	-24.92	Shell	*Macoma* cf. *tokyoensis* Makiyama	5140 ± 50	5330–5600	5510	DF	RW	110	5400	-28	9	NIES-TERRA-b082604a15	Tanabe et al. (2015)
GS-MHI-1	28.33	-24.92	Shell	*Ringiculina doliaris* (Gould)	5070 ± 50	5310–5550	5430	DF	DA	———	———	———	———	NIES-TERRA-b082604a18	Tanabe et al. (2015)
GS-MHI-1	29.21	-25.80	Shell	*Macoma* cf. *tokyoensis* Makiyama	5300 ± 50	5570–5800	5660	DF	DA	———	———	———	———	NIES-TERRA-b082604a19	Tanabe et al. (2015)
GS-MHI-1	29.82	-26.41	Shell	*Ringiculina doliaris* (Gould)	5310 ± 50	5570–5810	5670	DF	DA	———	———	———	———	NIES-TERRA-b082604a31	Tanabe et al. (2015)
GS-MHI-1	30.95	-27.54	Shell	*Ringiculina doliaris* (Gould)	6120 ± 50	6420–6670	6550	DF	DA	———	———	———	———	NIES-TERRA-b082604a20	Tanabe et al. (2015)
GS-MHI-1	31.49	-28.08	Shell	*Ringiculina doliaris* (Gould)	6500 ± 60	6850–7160	7010	EF	DA	———	———	———	———	NIES-TERRA-b082604a34	Tanabe et al. (2015)
GS-MHI-1	33.78	-30.37	Shell	Shell fragments	6630 ± 50	7010–7260	7160	EF	RW	110	7050	-33	13	NIES-TERRA-b082604a22	Tanabe et al. (2015)
GS-MHI-1	34.36	-30.95	Plant		6140 ± 70	6810–7240	7040	EF	DA	———	———	———	———	NIES-TERRA-b083104a20	Tanabe et al. (2015)
GS-MHI-1	34.35	-30.94	Shell	Shell fragments	7100 ± 50	7480–7670	7580	EF	RW	510	7070	-34	31	NIES-TERRA-b082604a17	Tanabe et al. (2015)
GS-MHI-1	34.35	-30.94	Shell	*Crassostrea* sp.	7990 ± 50	8350–8560	8450	EF	RW	1400	7050	-34	31	NIES-TERRA-b082604a23	Tanabe et al. (2015)
GS-MHI-1	35.67	-32.26	Plant		8530 ± 60	9430–9600	9520	TF	DA	———	———	———	———	NIES-TERRA-b083104a23	Tanabe et al. (2015)
GS-MHI-1	37.64	-34.23	Plant		8830 ± 60	9690–10170	9900	TF	DA	———	———	———	———	NIES-TERRA-b083104a24	Tanabe et al. (2015)
GS-MHI-1	38.97	-35.56	Plant		9180 ± 60	10230–10500	10350	TF	RW	370	9980	-7	82	NIES-TERRA-b083104a25	Tanabe et al. (2015)
GS-MHI-1	39.87	-36.46	Plant		9200 ± 60	10240–10510	10370	MR	RW	340	10030	-7	82	NIES-TERRA-b083104a26	Tanabe et al. (2015)
GS-MHI-1	40.40	-36.99	Plant		8950 ± 70	9790–10240	10050	MR	DA	———	———	———	———	NIES-TERRA-b082604a37	Tanabe et al. (2015)
GS-MHI-1	43.96	-40.55	Plant		9430 ± 60	10500–11070	10670	MR	RW	230	10440	-1	1	NIES-TERRA-b090404a05	Tanabe et al. (2015)
GS-MHI-1	44.08	-40.67	Plant		9640 ± 80	10750–11200	10970	MR	RW	510	10460	0	1	NIES-TERRA-b083104a31	Tanabe et al. (2015)
GS-MHI-1	44.17	-40.76	Plant		9750 ± 50	10900–11250	11190	MR	RW	740	10450	0	4	NIES-TERRA-b083104a32	Tanabe et al. (2015)
GS-MHI-1	45.77	-42.36	Plant		9380 ± 50	10440–10730	10610	MR	DA	———	———	———	———	NIES-TERRA-b090404a06	Tanabe et al. (2015)
GS-MHI-1	46.16	-42.75	Plant		9480 ± 60	10570–11080	10750	MR	DA	———	———	———	———	NIES-TERRA-b083104a34	Tanabe et al. (2015)
GS-MHI-1	47.60	-44.19	Plant		9800 ± 60	11100–11330	11220	MR	DA	———	———	———	———	NIES-TERRA-b083104a35	Tanabe et al. (2015)
GS-MHI-1	48.60	-45.19	Plant		10520 ± 70	12150–12660	12470	MR	DA	———	———	———	———	NIES-TERRA-b083104a36	Tanabe et al. (2015)
GS-MHI-1	49.88	-46.47	Plant		10580 ± 60	12410–12690	12560	MR	DA	———	———	———	———	NIES-TERRA-b083104a37	Tanabe et al. (2015)

GS-SK-1	3.25	0.48	Plant		1230 ± 40	1060–1270	1160	MF	DA	———	———	———	———	Beta-177911	Tanabe et al. (2015)
GS-SK-1	4.60	-0.87	Plant		2420 ± 40	2350–2700	2460	MF	RW	370	2090	-1	80	NIES-TERRA-b052406a18	Tanabe et al. (2015)
GS-SK-1	5.25	-1.52	Plant		2340 ± 40	2190–2650	2360	MF	DA	———	———	———	———	NIES-TERRA-b060806a13	Tanabe et al. (2015)
GS-SK-1	6.80	-3.07	Plant		4020 ± 40	4410–4780	4490	DF	DA	———	———	———	———	NIES-TERRA-b052406a21	Tanabe et al. (2015)
GS-SK-1	6.89	-3.16	Shell	*Crassostrea* sp.	4630 ± 40	4770–4980	4850	DF	RW	370	4480	-6	61	Beta-177912	Tanabe et al. (2015)
GS-SK-1	8.20	-4.47	Plant		4260 ± 40	4650–4960	4840	DF	RW	160	4680	-7	6	NIES-TERRA-b052406a22	Tanabe et al. (2015)
GS-SK-1	8.28	-4.55	Shell	Shell fragments	4500 ± 40	4570–4810	4700	DF	DA	———	———	———	———	Beta-183665	Tanabe et al. (2015)
GS-SK-1	10.22	-6.49	Shell	*Dosinella angulosa* (Philippi)	6590 ± 40	6990–7220	7110	DF	RW	2210	4900	-9	12	Beta-177913	Tanabe et al. (2015)
GS-SK-1	10.20	-6.47	Plant		4300 ± 40	4830–4970	4860	DF	DA	———	———	———	———	NIES-TERRA-b052406a23	Tanabe et al. (2015)
GS-SK-1	13.42	-9.69	Shell	*Clementia vatheleti* Mabille	6600 ± 40	7000–7230	7130	EF	DA	———	———	———	———	Beta-177914	Tanabe et al. (2015)
GS-SK-1	15.10	-11.37	Shell	*Cadella delta* (Yokoyama)	7470 ± 40	7830–8010	7930	EF	RW	40	7890	-8	72	Beta-183666	Tanabe et al. (2015)
GS-SK-1	15.10	-11.37	Shell	*Veremolpa micra* (Pilsbry)	7430 ± 50	7780–7990	7890	EF	DA	———	———	———	———	NIES-TERRA-b060206a35	Tanabe et al. (2015)
GS-SK-1	17.05	-13.32	Shell	*Crassostrea* sp.	7540 ± 40	7920–8110	7990	EF	DA	———	———	———	———	Beta-183667	Tanabe et al. (2015)
GS-SK-1	17.20	-13.47	Shell	*Crassostrea* sp.	7610 ± 40	7970–8160	8070	EF	DA	———	———	———	———	Beta-177915	Tanabe et al. (2015)
GS-SK-1	17.40	-13.67	Plant		15800 ± 70	18880–19250	19050	EF	RW	11000	———	———	———	NIES-TERRA-b052406a26	Tanabe et al. (2015)
GS-SK-1	18.65	-14.92	Shell	Shell fragments	7620 ± 40	7980–8170	8080	EF	DA	———	———	———	———	Beta-183668	Tanabe et al. (2015)
GS-SK-1	20.20	-16.47	Plant		9720 ± 50	10870–11240	11160	EF	RW	2530	8630	-7	8	NIES-TERRA-b052406a27	Tanabe et al. (2015)
GS-SK-1	20.55	-16.82	Shell	*Potamocorbula* sp.	8280 ± 40	8670–8970	8830	TF	DA	———	———	———	———	Beta-177916	Tanabe et al. (2015)
GS-SK-1	22.55	-18.82	Shell	*Potamocorbula* sp.	8480 ± 40	8990–9230	9090	TF	DA	———	———	———	———	Beta-177917	Tanabe et al. (2015)
GS-SK-1	23.80	-20.07	Shell	*Potamocorbula* sp.	8490 ± 40	9000–9240	9110	TF	DA	———	———	———	———	Beta-177918	Tanabe et al. (2015)
GS-SK-1	23.80	-20.07	Plant		8450 ± 50	9320–9540	9480	TF	RW	370	9110	-1	3	NIES-TERRA-b052406a28	Tanabe et al. (2015)
GS-SK-1	24.41	-20.68	Shell	Shell fragments	8520 ± 40	9020–9270	9150	TF	DA	———	———	———	———	Beta-177919	Tanabe et al. (2015)
GS-SK-1	30.52	-26.79	Plant		8730 ± 40	9560–9890	9690	TF	DA	———	———	———	———	Beta-177921	Tanabe et al. (2015)
GS-SK-1	32.70	-28.97	Plant		8870 ± 50	9770–10180	10000	TF	RW	30	9970	0	1	NIES-TERRA-b052406a29	Tanabe et al. (2015)
GS-SK-1	32.75	-29.02	Shell	*Corbicula japonica* Prime	9170 ± 40	9790–10130	9970	TF	DA	———	———	———	———	Beta-177922	Tanabe et al. (2015)
GS-SK-1	40.70	-36.97	Plant		9370 ± 70	10300–10770	10590	MR	DA	———	———	———	———	NIES-TERRA-b052406a32	Tanabe et al. (2015)
GS-SK-1	43.15	-39.42	Plant		9620 ± 40	10780–11170	10940	MR	DA	———	———	———	———	Beta-177925	Tanabe et al. (2015)
GS-SK-1	47.60	-43.87	Plant		9720 ± 60	10800–11250	11150	MR	DA	———	———	———	———	NIES-TERRA-b052406a33	Tanabe et al. (2015)
GS-SK-1	58.72	-54.99	Plant		> 48350	———	———	SH	Base	———	———	———	———	Beta-180986	Tanabe et al. (2015)
GS-SMB-1	2.34	0.74	Plant		1130 ± 40	960–1170	1040	MF	DA	———	———	———	———	NIES-TERRA-b042407a22	Tanabe et al. (2015)
GS-SMB-1	4.47	-1.39	Plant		4510 ± 50	4980–5310	5160	MF	DA	———	———	———	———	NIES-TERRA-b042407a03	Tanabe et al. (2015)
GS-SMB-1	5.78	-2.70	Plant		4820 ± 50	5330–5650	5530	DF	DA	———	———	———	———	NIES-TERRA-b042407a04	Tanabe et al. (2015)
GS-SMB-1	5.95	-2.87	Shell	*Rapana venosa* (Valenciennes)	5200 ± 40	5460–5640	5560	DF	DA	———	———	———	———	NIES-TERRA-b041207a18	Tanabe et al. (2015)
GS-SMB-1	7.55	-4.47	Shell	*Crassostrea* sp.	5940 ± 50	6260–6470	6350	DF	RW	680	5670	-7	67	NIES-TERRA-b041207a20	Tanabe et al. (2015)
GS-SMB-1	8.87	-5.79	Shell	*Potamocorbula* sp.	6460 ± 40	6840–7100	6950	DF	RW	1110	5840	-9	9	NIES-TERRA-b052407a03	Tanabe et al. (2015)
GS-SMB-1	9.19	-6.11	Plant		5010 ± 70	5610–5900	5760	DF	DA	———	———	———	———	NIES-TERRA-b042407a05	Tanabe et al. (2015)
GS-SMB-1	10.20	-7.12	Shell	*Potamocorbula* sp.	6400 ± 50	6730–7010	6880	DF	DA	———	———	———	———	NIES-TERRA-b041207a21	Tanabe et al. (2015)
GS-SMB-1	11.75	-8.67	Plant		6350 ± 50	7170–7420	7290	EF	RW	320	6970	-12	5	NIES-TERRA-b042407a06	Tanabe et al. (2015)
GS-SMB-1	14.90	-11.82	Plant		6280 ± 50	7020–7310	7210	EF	RW	160	7050	-15	26	NIES-TERRA-b042407a08	Tanabe et al. (2015)
GS-SMB-1	14.90	-11.82	Shell	*Raetellops pulchellus* (Adams et Reeve)	6770 ± 40	7220–7400	7300	EF	RW	210	7090	-15	26	NIES-TERRA-b041207a22	Tanabe et al. (2015)
GS-SMB-1	17.19	-14.11	Plant		6250 ± 60	6990–7290	7180	EF	DA	———	———	———	———	NIES-TERRA-b042407a09	Tanabe et al. (2015)
GS-SMB-1	17.29	-14.21	Shell	*Raeta pellicula* (Reeve)	7050 ± 60	7430–7640	7530	EF	DA	———	———	———	———	NIES-TERRA-b041207a23	Tanabe et al. (2015)
GS-SMB-1	19.90	-16.82	Shell	*Ringiculina doliaris* (Gould)	7410 ± 40	7780–7960	7880	EF	RW	160	7720	-15	28	NIES-TERRA-b052407a04	Tanabe et al. (2015)
GS-SMB-1	20.95	-17.87	Plant		7910 ± 70	8590–8990	8760	EF	RW	1050	7710	-16	34	NIES-TERRA-b052407a08	Tanabe et al. (2015)
GS-SMB-1	23.51	-20.43	Plant		7050 ± 60	7740–7980	7880	EF	DA	———	———	———	———	NIES-TERRA-b042407a10	Tanabe et al. (2015)
GS-SMB-1	25.60	-22.52	Shell	*Mitrella yabei* (Nomura)	7720 ± 50	8050–8310	8190	EF	DA	———	———	———	———	NIES-TERRA-b041207a24	Tanabe et al. (2015)
GS-SMB-1	26.90	-23.82	Shell	*Potamocorbula* sp.	8580 ± 40	9090–9370	9230	EF	RW	790	8440	-16	12	NIES-TERRA-b052407a05	Tanabe et al. (2015)
GS-SMB-1	27.10	-24.02	Shell	*Limaria hakodatensis* (Tokunaga)	7970 ± 50	8330–8540	8430	EF	DA	———	———	———	———	NIES-TERRA-b041207a29	Tanabe et al. (2015)
GS-SMB-1	27.20	-24.12	Plant		7750 ± 60	8420–8630	8520	EF	RW	50	8470	-16	32	NIES-TERRA-b042407a11	Tanabe et al. (2015)
GS-SMB-1	27.20	-24.12	Shell	*Crassostrea* sp.	8010 ± 50	8370–8580	8470	EF	DA	———	———	———	———	NIES-TERRA-b041207a27	Tanabe et al. (2015)
GS-SMB-1	28.00	-24.92	Shell	*Potamocorbula* sp.	8580 ± 50	9070–9390	9230	EF	RW	580	8650	-15	8	NIES-TERRA-b041207a28	Tanabe et al. (2015)
GS-SMB-1	28.70	-25.62	Plant		8000 ± 60	8650–9010	8860	EF	DA	———	———	———	———	NIES-TERRA-b052407a09	Tanabe et al. (2015)
GS-SMB-1	30.30	-27.22	Plant		8130 ± 50	8990–9260	9080	EF	DA	———	———	———	———	NIES-TERRA-b052407a10	Tanabe et al. (2015)
GS-SMB-1	32.10	-29.02	Plant		9120 ± 70	10180–10490	10300	EF	RW	260	10040	0	3	NIES-TERRA-b042407a12	Tanabe et al. (2015)
TN	2.63	0.25	Plant		1740 ± 40	1550–1770	1650	MT	DA	———	———	———	———	Beta-175708	Tanabe et al. (2015)
TN	8.66	-5.78	Shell	*Cryptomya busoensis* Yokoyama	5780 ± 50	6060–6300	6210	DF	DA	———	———	———	———	Beta-175710	Tanabe et al. (2015)
TN	10.70	-7.82	Shell	*Cryptomya busoensis* Yokoyama	6290 ± 50	6630–6880	6750	DF	RW	470	6280	-11	67	Beta-175711	Tanabe et al. (2015)
TN	13.10	-10.22	Shell	Shell fragments	5840 ± 50	6160–6380	6260	DF	DA	———	———	———	———	Beta-175712	Tanabe et al. (2015)
TN	16.52	-13.64	Shell	Shell fragments	6250 ± 50	6570–6840	6700	DF	DA	———	———	———	———	Beta-175713	Tanabe et al. (2015)
TN	20.40	-17.52	Shell	*Crassostrea* sp.	7970 ± 60	8320–8560	8430	EF	DA	———	———	———	———	Beta-175714	Tanabe et al. (2015)
TN	22.10	-19.22	Shell	*Mactra chinensis* Philippi	8400 ± 60	8780–9190	9000	EF	RW	580	8420	-11	3	Beta-175715	Tanabe et al. (2015)
TN	32.40	-29.52	Plant		7860 ± 60	8540–8980	8680	EF	DA	———	———	———	———	Beta-175717	Tanabe et al. (2015)
MZ	7.80	-5.95	Shell	*Mactra chinensis* Philippi	3420 ± 50	3150–3410	3290	MF	DA	———	———	———	———	Beta-175720	Tanabe et al. (2015)
MZ	9.40	-7.55	Shell	*Mactra chinensis* Philippi	4680 ± 50	4800–5040	4910	SP	RW	1470	3440	-8	70	Beta-175721	Tanabe et al. (2015)
MZ	12.25	-10.40	Shell	*Cryptomya busoensis* Yokoyama	3800 ± 50	3600–3880	3750	SP	DA	———	———	———	———	Beta-175722	Tanabe et al. (2015)
MZ	16.15	-14.30	Shell	Shell fragments	6320 ± 50	6650–6910	6780	SP	RW	2840	3940	-17	98	Beta-175723	Tanabe et al. (2015)
MZ	19.60	-17.75	Shell	Shell fragments	4150 ± 40	4090–4360	4220	SP	RW	100	4120	-21	96	Beta-175724	Tanabe et al. (2015)
MZ	21.50	-19.65	Shell	*Mactra chinensis* Philippi	4150 ± 40	4090–4360	4220	SP	DA	———	———	———	———	Beta-175725	Tanabe et al. (2015)
MZ	23.30	-21.45	Shell	Shell fragments	4210 ± 50	4140–4430	4300	SP	DA	———	———	———	———	Beta-175726	Tanabe et al. (2015)
MZ	26.75	-24.90	Plant		3910 ± 40	4180–4500	4340	SP	RW	0	4340	-28	8	Beta-175727	Tanabe et al. (2015)
MZ	29.20	-27.35	Plant		3900 ± 40	4160–4430	4340	SP	DA	———	———	———	———	Beta-175728	Tanabe et al. (2015)
MZ	32.95	-31.10	Shell	*Babylonia japonica* (Reeve)	5950 ± 50	6270–6480	6360	SP	DA	———	———	———	———	Beta-175729	Tanabe et al. (2015)
MZ	35.90	-34.05	Shell	*Potamocorbula* sp.	7470 ± 60	7790–8060	7930	EF	DA	———	———	———	———	Beta-175730	Tanabe et al. (2015)
MZ	38.70	-36.85	Plant		8410 ± 60	9300–9530	9440	TF	DA	———	———	———	———	Beta-175731	Tanabe et al. (2015)
MZ	46.00	-44.15	Plant		10050 ± 40	11340–11770	11560	MR	DA	———	———	———	———	Beta-189141	Tanabe et al. (2015)
MZ	49.70	-47.85	Plant		10470 ± 40	12140–12560	12430	MR	DA	———	———	———	———	Beta-189142	Tanabe et al. (2015)
MZ	52.10	-50.25	Plant		11150 ± 40	12900–13110	13040	MR	DA	———	———	———	———	Beta-189143	Tanabe et al. (2015)

GS-AHH-1	2.37	1.27	Shell	Shell fragments	4340 ± 40	4370–4600	4470	MT	DA	———	———	———	———	NIES-TERRA-b041207a03	Tanabe et al. (2015)
GS-AHH-1	4.82	-2.28	Shell	*Cryptomya busoensis* (Yokoyama)	5070 ± 90	5230–5620	5420	DF	DA	———	———	———	———	NIES-TERRA-b041207a04	Tanabe et al. (2015)
GS-AHH-1	4.97	-2.43	Shell	*Crassostrea* sp.	5250 ± 40	5530–5720	5610	DF	RW	160	5450	-5	49	NIES-TERRA-b041207a05	Tanabe et al. (2015)
GS-AHH-1	5.48	-2.94	Shell	Shell fragments	5230 ± 40	5480–5680	5590	DF	DA	———	———	———	———	NIES-TERRA-b041207a06	Tanabe et al. (2015)
GS-AHH-1	6.81	-4.27	Shell	*Veremolpa micra* (Pilsbry)	5250 ± 40	5530–5720	5610	DF	DA	———	———	———	———	NIES-TERRA-b041207a08	Tanabe et al. (2015)
GS-AHH-1	7.78	-5.24	Shell	*Veremolpa micra* (Pilsbry)	5340 ± 40	5600–5830	5700	DF	DA	———	———	———	———	NIES-TERRA-b041207a09	Tanabe et al. (2015)
GS-AHH-1	9.36	-6.82	Shell	*Dosinella angulosa* (Philippi)	6390 ± 40	6750–6980	6860	DF	DA	———	———	———	———	NIES-TERRA-b041207a10	Tanabe et al. (2015)
GS-AHH-1	12.55	-10.01	Crab		7260 ± 50	7610–7840	7720	DF	RW	420	7300	-11	8	NIES-TERRA-b041207a11	Tanabe et al. (2015)
GS-AHH-1	13.10	-10.56	Shell	*Dosinella angulosa* (Philippi)	6810 ± 40	7250–7410	7340	EF	DA	———	———	———	———	NIES-TERRA-b041207a12	Tanabe et al. (2015)
GS-AHH-1	13.45	-10.91	Shell	*Veremolpa micra* (Pilsbry)	7400 ± 50	7740–7960	7870	EF	DA	———	———	———	———	NIES-TERRA-b041207a15	Tanabe et al. (2015)
GS-AHH-1	14.23	-11.69	Shell	*Dosinella angulosa* (Philippi)	7870 ± 50	8200–8420	8340	EF	DA	———	———	———	———	NIES-TERRA-b041207a16	Tanabe et al. (2015)
GS-AHH-1	15.94	-13.40	Shell	*Crassostrea* sp.	8440 ± 50	8940–9230	9050	EF	RW	370	8680	-3	11	NIES-TERRA-b041207a17	Tanabe et al. (2015)
GS-AHH-1	16.20	-13.66	Plant		7870 ± 60	8550–8980	8690	TF	DA	———	———	———	———	NIES-TERRA-b042407a15	Tanabe et al. (2015)
GS-AHH-1	17.28	-14.74	Plant		8070 ± 60	8720–9240	8990	TF	RW	210	8780	-1	1	NIES-TERRA-b042407a16	Tanabe et al. (2015)
GS-AHH-1	17.91	-15.37	Plant		8210 ± 60	9010–9400	9180	TF	RW	370	8810	-1	1	NIES-TERRA-b042407a17	Tanabe et al. (2015)
GS-AHH-1	18.49	-15.95	Plant		8040 ± 60	8650–9090	8900	TF	DA	———	———	———	———	NIES-TERRA-b042407a18	Tanabe et al. (2015)
GS-AHH-1	20.37	-17.83	Plant		8170 ± 70	8990–9400	9130	TF	DA	———	———	———	———	NIES-TERRA-b042407a20	Tanabe et al. (2015)
GS-AHH-1	22.57	-20.03	Plant		37800 ± 300	———	———	SH	Base	———	———	———	———	NIES-TERRA-b042407a21	Tanabe et al. (2015)
HA	3.20	-3.23	Shell	*Mya japonica* Jay	4490 ± 40	4560–4810	4690	DF	RW	3160	1530	-3	76	Beta-176647	Tanabe et al. (2015)
HA	5.75	-5.78	Plant		2470 ± 60	2360–2720	2560	DF	DA	———	———	———	———	Beta-176648	Tanabe et al. (2015)
HA	8.55	-8.58	Shell	*Dosinella angulosa* (Philippi)	4700 ± 40	4830–5040	4920	DF	DA	———	———	———	———	Beta-176649	Tanabe et al. (2015)
HA	11.70	-11.73	Shell	*Dosinella angulosa* (Philippi)	5050 ± 40	5300–5520	5400	DF	RW	420	4980	-15	26	Beta-176650	Tanabe et al. (2015)
HA	13.80	-13.83	Plant		4440 ± 40	4880–5280	5050	DF	DA	———	———	———	———	Beta-176651	Tanabe et al. (2015)
HA	15.80	-15.83	Shell	*Dosinella angulosa* (Philippi)	5710 ± 40	5990–6230	6120	DF	DA	———	———	———	———	Beta-176652	Tanabe et al. (2015)
HA	23.60	-23.63	Shell	*Mactra* cf. *chinensis* Philippi	8470 ± 40	8980–9220	9080	EF	DA	———	———	———	———	Beta-176654	Tanabe et al. (2015)
HA	25.49	-25.52	Plant		8410 ± 40	9310–9520	9450	TF	DA	———	———	———	———	Beta-176655	Tanabe et al. (2015)
HA	28.50	-28.53	Plant		8630 ± 40	9530–9680	9580	TF	DA	———	———	———	———	Beta-176656	Tanabe et al. (2015)
HA	34.65	-34.68	Shell	*Corbicula* cf. *japonica* Prime	9280 ± 40	9950–10210	10130	TF	DA	———	———	———	———	Beta-176657	Tanabe et al. (2015)
HA	37.05	-37.08	Plant		9100 ± 40	10200–10380	10250	TF	DA	———	———	———	———	Beta-176658	Tanabe et al. (2015)
HA	42.90	-42.93	Plant		9580 ± 40	10740–11120	10930	MR	DA	———	———	———	———	Beta-176659	Tanabe et al. (2015)
HA	48.15	-48.18	Plant		10390 ± 40	12060–12420	12260	MR	DA	———	———	———	———	Beta-176661	Tanabe et al. (2015)
HA	51.30	-51.33	Plant		11330 ± 40	13090–13270	13180	MR	DA	———	———	———	———	Beta-176662	Tanabe et al. (2015)
GS-KNJ-1	7.02	-6.59	Plant		2530 ± 40	2490–2750	2610	MF	DA	———	———	———	———	Beta-189144	Tanabe et al. (2015)
GS-KNJ-1	7.65	-7.22	Shell	*Potamocorbula* sp.	3330 ± 40	3060–3320	3180	DF	DA	———	———	———	———	Beta-189145	Tanabe et al. (2015)
GS-KNJ-1	10.80	-10.37	Shell	*Potamocorbula* sp.	3430 ± 40	3180–3400	3300	DF	DA	———	———	———	———	Beta-189120	Tanabe et al. (2015)
GS-KNJ-1	13.65	-13.22	Echinoderm		3450 ± 40	3210–3430	3330	DF	DA	———	———	———	———	Beta-189121	Tanabe et al. (2015)
GS-KNJ-1	15.77	-15.34	Shell	*Potamocorbula* sp.	3710 ± 70	3450–3820	3630	DF	DA	———	———	———	———	NIES-TERRA-b122504a33	Tanabe et al. (2015)
GS-KNJ-1	16.48	-16.05	Shell	Gastropoda gen. et sp. indet.	6270 ± 50	6600–6870	6720	SP	RW	2950	3770	-18	28	Beta-189122	Tanabe et al. (2015)
GS-KNJ-1	17.84	-17.41	Shell	Shell fragments	8060 ± 50	8390–8630	8510	SP	RW	4530	3980	-20	48	Beta-189123	Tanabe et al. (2015)
GS-KNJ-1	18.46	-18.03	Shell	Gastropoda gen. et sp. indet.	7620 ± 40	7980–8170	8080	SP	RW	4110	3970	-21	77	Beta-189124	Tanabe et al. (2015)
GS-KNJ-1	19.40	-18.97	Shell	Bivalvia gen. et sp. indet.	5880 ± 40	6210–6390	6300	SP	RW	2160	4140	-22	53	Beta-189125	Tanabe et al. (2015)
GS-KNJ-1	20.73	-20.30	Shell	*Veremolpa micra* (Pilsbry)	5050 ± 40	5300–5520	5400	SP	RW	1160	4240	-23	29	Beta-189126	Tanabe et al. (2015)
GS-KNJ-1	22.10	-21.67	Shell	*Solen* sp.	4290 ± 100	4120–4760	4410	SP	DA	———	———	———	———	NIES-TERRA-b122504a34	Tanabe et al. (2015)
GS-KNJ-1	24.72	-24.29	Shell	*Solen* sp.	6360 ± 40	6720–6940	6830	SP	RW	2260	4570	-27	30	Beta-189127	Tanabe et al. (2015)
GS-KNJ-1	25.73	-25.30	Shell	*Veremolpa micra* (Pilsbry)	6080 ± 50	6390–6630	6510	SP	RW	1890	4620	-28	37	NIES-TERRA-b122504a36	Tanabe et al. (2015)
GS-KNJ-1	26.78	-26.35	Shell	Shell fragments	4510 ± 40	4580–4820	4710	SP	DA	———	———	———	———	NIES-TERRA-b122504a37	Tanabe et al. (2015)
GS-KNJ-1	27.16	-26.73	Shell	Shell fragments	5580 ± 40	5880–6100	5970	SP	RW	1210	4760	-30	60	NIES-TERRA-b122504a38	Tanabe et al. (2015)
GS-KNJ-1	27.82	-27.39	Shell	Shell fragments	4660 ± 40	4800–5000	4880	SP	DA	———	———	———	———	Beta-189128	Tanabe et al. (2015)
GS-KNJ-1	29.45	-29.02	Shell	Shell fragments	6550 ± 120	6760–7310	7050	SP	RW	1580	5470	-32	68	NIES-TERRA-b122504a39	Tanabe et al. (2015)
GS-KNJ-1	31.05	-30.62	Shell	Shell fragments	5710 ± 40	5990–6230	6120	SP	DA	———	———	———	———	Beta-189129	Tanabe et al. (2015)
GS-KNJ-1	33.56	-33.13	Shell	*Potamocorbula* sp.	6310 ± 50	6640–6900	6770	SP	DA	———	———	———	———	Beta-189130	Tanabe et al. (2015)
GS-KNJ-1	36.15	-35.72	Shell	*Panopea japonica* A. Adams	6360 ± 50	6690–6960	6830	SP	DA	———	———	———	———	Beta-189131	Tanabe et al. (2015)
GS-KNJ-1	36.69	-36.26	Shell	*Potamocorbula* sp.	9180 ± 80	9690–10180	9960	SP	RW	320	9640	-9	69	NIES-TERRA-b013105a04	Tanabe et al. (2015)
GS-KNJ-1	37.30	-36.87	Shell	*Potamocorbula* sp.	8980 ± 50	9520–9820	9640	TF	DA	———	———	———	———	NIES-TERRA-b013105a05	Tanabe et al. (2015)
GS-KNJ-1	38.12	-37.69	Shell	*Potamocorbula* sp.	9070 ± 60	9550–10020	9780	TF	DA	———	———	———	———	NIES-TERRA-b013105a06	Tanabe et al. (2015)
GS-KNJ-1	39.40	-38.97	Shell	*Potamocorbula* sp.	9110 ± 40	9680–10070	9850	TF	DA	———	———	———	———	Beta-189132	Tanabe et al. (2015)
GS-KNJ-1	44.30	-43.87	Plant		8930 ± 180	9550–10480	10000	MR	DA	———	———	———	———	NIES-TERRA-b011005a08	Tanabe et al. (2015)
GS-KNJ-1	45.50	-45.07	Plant		9520 ± 70	10590–11110	10860	MR	DA	———	———	———	———	NIES-TERRA-b010505a28	Tanabe et al. (2015)
GS-KNJ-1	45.90	-45.47	Plant		10010 ± 40	11290–11710	11490	MR	DA	———	———	———	———	Beta-189134	Tanabe et al. (2015)
GS-KNJ-1	49.62	-49.19	Plant		10300 ± 50	11840–12390	12090	MR	DA	———	———	———	———	Beta-189135	Tanabe et al. (2015)
GS-KNJ-1	52.30	-51.87	Plant		10850 ± 40	12690–12790	12730	MR	DA	———	———	———	———	Beta-189136	Tanabe et al. (2015)
GS-KNJ-1	54.10	-53.67	Bulk sediments		12090 ± 50	13780–14100	13940	MR	DA	———	———	———	———	Beta-189137	Tanabe et al. (2015)
GS-KNJ-1	69.45	-69.02	Shell	*Mactra chinensis* Philippi	>45520	———	———	SH	Base	———	———	———	———	Beta-189139	Tanabe et al. (2015)
GS-AMG-1	2.16	0.51	Plant		2230 ± 40	2150–2340	2230	MT	DA	———	———	———	———	NIES-TERRA-b081205a03	Tanabe et al. (2015)
GS-AMG-1	2.42	-0.75	Plant		4680 ± 50	5310–5580	5410	MT	RW	2630	2780	1	87	NIES-TERRA-b081205a04	Tanabe et al. (2015)
GS-AMG-1	2.77	-1.10	Plant		4020 ± 40	4410–4780	4490	MT	RW	1740	2750	1	52	NIES-TERRA-b081205a05	Tanabe et al. (2015)
GS-AMG-1	2.98	-0.31	Plant		2630 ± 40	2720–2840	2760	MT	DA	———	———	———	———	NIES-TERRA-b081205a06	Tanabe et al. (2015)
GS-AMG-1	3.83	-2.16	Plant		4650 ± 40	5310–5570	5400	DF	DA	———	———	———	———	NIES-TERRA-b081205a09	Tanabe et al. (2015)
GS-AMG-1	4.90	-3.23	Plant		4690 ± 50	5320–5580	5420	DF	DA	———	———	———	———	NIES-TERRA-b081205a10	Tanabe et al. (2015)
GS-AMG-1	7.88	-6.21	Plant		4900 ± 80	5470–5890	5650	DF	DA	———	———	———	———	NIES-TERRA-b081205a11	Tanabe et al. (2015)
GS-AMG-1	9.35	-7.68	Shell	*Dosinella angulosa* (Philippi)	5890 ± 70	6170–6460	6310	DF	DA	———	———	———	———	NIES-TERRA-b080505a03	Tanabe et al. (2015)
GS-AMG-1	10.63	-8.96	Shell	*Dosinella angulosa* (Philippi)	6140 ± 40	6450–6670	6570	DF	DA	———	———	———	———	NIES-TERRA-b080505a04	Tanabe et al. (2015)
GS-AMG-1	11.97	-10.30	Shell	*Dosinella angulosa* (Philippi)	6700 ± 40	7150–7320	7230	DF	RW	580	6650	-13	31	NIES-TERRA-b080505a05	Tanabe et al. (2015)
GS-AMG-1	14.30	-12.63	Shell	*Dosinella angulosa* (Philippi)	6960 ± 70	7330–7580	7460	DF	RW	790	6670	-16	33	NIES-TERRA-b080505a06	Tanabe et al. (2015)
GS-AMG-1	15.46	-13.79	Shell	Shell fragments	6220 ± 70	6480–6840	6670	DF	DA	———	———	———	———	NIES-TERRA-b080505a08	Tanabe et al. (2015)
GS-AMG-1	16.75	-15.08	Shell	Shell fragments	7550 ± 50	7920–8140	8010	EF	DA	———	———	———	———	NIES-TERRA-b080505a09	Tanabe et al. (2015)
GS-AMG-1	17.50	-15.83	Shell	*Dosinella angulosa* (Philippi)	7760 ± 50	8120–8350	8230	EF	DA	———	———	———	———	NIES-TERRA-b080505a10	Tanabe et al. (2015)

GS-AMG-1	19.25	-17.58	Shell	Shell fragments	8450 ± 50	8950–9230	9060	EF	RW	790	8270	-11	79	NIES-TERRA-b080505a11	Tanabe et al. (2015)
GS-AMG-1	20.17	-18.50	Shell	Shell fragments	8200 ± 50	8550–8910	8700	EF	RW	420	8280	-12	70	NIES-TERRA-b080505a13	Tanabe et al. (2015)
GS-AMG-1	22.00	-20.33	Plant		7870 ± 50	8550–8980	8680	EF	RW	370	8310	-13	52	NIES-TERRA-b082205a29	Tanabe et al. (2015)
GS-AMG-1	23.00	-21.33	Shell	*Potamocorbula* sp.	8620 ± 130	8970–9520	9260	EF	RW	890	8370	-13	64	NIES-TERRA-b080505a14	Tanabe et al. (2015)
GS-AMG-1	23.24	-21.57	Shell	Shell fragments	8690 ± 50	9240–9470	9360	EF	RW	1000	8360	-14	80	NIES-TERRA-b080505a15	Tanabe et al. (2015)
GS-AMG-1	24.11	-22.44	Plant		7800 ± 50	8440–8710	8580	EF	RW	160	8420	-14	22	NIES-TERRA-b081205a13	Tanabe et al. (2015)
GS-AMG-1	24.17	-22.50	Plant		7960 ± 40	8650–8990	8840	EF	RW	470	8370	-15	22	Beta-209334	Tanabe et al. (2015)
GS-AMG-1	25.31	-23.64	Plant		8210 ± 60	9010–9400	9180	EF	RW	740	8440	-16	22	Beta-209335	Tanabe et al. (2015)
GS-AMG-1	25.44	-23.77	Plant		7580 ± 50	8220–8520	8390	EF	DA	———	———	———	———	NIES-TERRA-b081205a14	Tanabe et al. (2015)
GS-AMG-1	25.55	-23.88	Plant		8310 ± 50	9140–9460	9330	EF	RW	890	8440	-16	77	NIES-TERRA-b081205a15	Tanabe et al. (2015)
GS-AMG-1	26.92	-25.25	Plant		7710 ± 60	8400–8590	8500	EF	DA	———	———	———	———	NIES-TERRA-b081205a16	Tanabe et al. (2015)
GS-AMG-1	27.94	-26.27	Plant		8450 ± 60	9310–9540	9470	TF	DA	———	———	———	———	NIES-TERRA-b081205a19	Tanabe et al. (2015)
GS-AMG-1	29.75	-28.08	Plant		8460 ± 50	9330–9540	9480	TF	DA	———	———	———	———	NIES-TERRA-b082205a30	Tanabe et al. (2015)
GS-AMG-1	34.75	-33.08	Plant		8680 ± 60	9540–9890	9640	TF	DA	———	———	———	———	NIES-TERRA-b081205a20	Tanabe et al. (2015)
GS-AMG-1	36.78	-35.11	Plant		8770 ± 50	9560–10120	9780	TF	RW	110	9670	-7	7	NIES-TERRA-b081205a21	Tanabe et al. (2015)
GS-AMG-1	36.97	-35.30	Plant		8700 ± 50	9540–9890	9650	TF	DA	———	———	———	———	NIES-TERRA-b081205a22	Tanabe et al. (2015)
GS-AMG-1	39.32	-37.65	Plant		9130 ± 60	10200–10490	10300	TF	RW	370	9930	-9	2	NIES-TERRA-b081205a24	Tanabe et al. (2015)
GS-AMG-1	41.15	-39.48	Plant		8940 ± 60	9830–10230	10050	MR	DA	———	———	———	———	NIES-TERRA-b081205a25	Tanabe et al. (2015)
GS-AMG-1	43.38	-41.71	Plant		9230 ± 60	10250–10550	10400	MR	DA	———	———	———	———	NIES-TERRA-b081205a26	Tanabe et al. (2015)
GS-AMG-1	45.40	-43.73	Plant		9830 ± 60	11160–11390	11240	MR	RW	680	10560	-3	2	NIES-TERRA-b081205a27	Tanabe et al. (2015)
GS-AMG-1	47.45	-45.78	Plant		9350 ± 60	10300–10730	10570	MR	DA	———	———	———	———	NIES-TERRA-b081205a29	Tanabe et al. (2015)
GS-AMG-1	49.71	-48.04	Plant		10970 ± 60	12720–12990	12830	MR	RW	370	12460	8	69	NIES-TERRA-b081205a30	Tanabe et al. (2015)
GS-AMG-1	50.07	-48.40	Plant		10490 ± 90	12090–12650	12420	MR	DA	———	———	———	———	NIES-TERRA-b081205a31	Tanabe et al. (2015)
GS-KTS-1	3.45	-2.26	Plant		2500 ± 40	2430–2740	2590	MT	RW	1470	1120	-2	27	Beta-189819	Tanabe et al. (2015)
GS-KTS-1	4.40	-3.21	Plant		1500 ± 50	1310–1520	1390	TF	DA	———	———	———	———	NIES-TERRA-b122004a31	Tanabe et al. (2015)
GS-KTS-1	5.72	-4.53	Shell	*Umbonium* sp.	6840 ± 40	7270–7430	7360	SP	RW	1680	5680	-8	96	Beta-189820	Tanabe et al. (2015)
GS-KTS-1	7.33	-6.14	Shell	*Mactra chinensis* Philippi	5640 ± 40	5930–6160	6040	SP	RW	320	5720	-9	95	NIES-TERRA-b013105a07	Tanabe et al. (2015)
GS-KTS-1	8.27	-7.08	Shell	*Mactra chinensis* Philippi	5430 ± 50	5670–5910	5800	SP	DA	———	———	———	———	NIES-TERRA-b013105a08	Tanabe et al. (2015)
GS-KTS-1	9.71	-8.52	Shell	*Mactra chinensis* Philippi	5880 ± 40	6210–6390	6300	SP	RW	420	5880	-12	67	Beta-189821	Tanabe et al. (2015)
GS-KTS-1	13.10	-11.91	Shell	*Solen* sp.	5630 ± 40	5920–6160	6030	SP	DA	———	———	———	———	NIES-TERRA-b013105a10	Tanabe et al. (2015)
GS-KTS-1	15.74	-14.55	Shell	Shell fragments	5690 ± 40	5980–6210	6100	SP	DA	———	———	———	———	Beta-189822	Tanabe et al. (2015)
GS-KTS-1	17.15	-15.96	Shell	*Dosinella angulosa* (Philippi)	5930 ± 50	6250–6460	6340	SP	DA	———	———	———	———	NIES-TERRA-b013105a11	Tanabe et al. (2015)
GS-KTS-1	19.24	-18.05	Shell	*Lucinoma annulatum* (Reeve)	6170 ± 40	6490–6710	6610	SP	DA	———	———	———	———	Beta-189823	Tanabe et al. (2015)
GS-KTS-1	21.55	-20.36	Shell	*Moerella* sp.	6360 ± 40	6720–6940	6830	SP	DA	———	———	———	———	Beta-189824	Tanabe et al. (2015)
GS-KTS-1	23.77	-22.58	Shell	*Cadella delta* (Yokoyama)	6590 ± 50	6980–7240	7110	EF	DA	———	———	———	———	NIES-TERRA-b013105a12	Tanabe et al. (2015)
GS-KTS-1	27.80	-26.61	Shell	*Macoma* sp.	7060 ± 50	7440–7640	7540	EF	DA	———	———	———	———	NIES-TERRA-b013105a13	Tanabe et al. (2015)
GS-KTS-1	30.23	-29.04	Shell	*Raetellops pulchellus* (Adams et Reeve)	7860 ± 40	8210–8400	8330	EF	DA	———	———	———	———	Beta-189825	Tanabe et al. (2015)
GS-KTS-1	31.24	-30.05	Shell	Shell fragments	8030 ± 60	8360–8620	8490	EF	DA	———	———	———	———	NIES-TERRA-b013105a14	Tanabe et al. (2015)
GS-KTS-1	33.01	-31.82	Shell	*Potamocorbula* sp.	8650 ± 40	9200–9430	9330	EF	DA	———	———	———	———	Beta-189826	Tanabe et al. (2015)
GS-KTS-1	34.47	-33.28	Shell	*Potamocorbula* sp.	8970 ± 40	9520–9760	9620	EF	RW	110	9510	-7	11	Beta-189827	Tanabe et al. (2015)
GS-KTS-1	35.83	-34.64	Shell	*Potamocorbula* sp.	8900 ± 40	9470–9660	9540	EF	DA	———	———	———	———	Beta-189828	Tanabe et al. (2015)
GS-KTS-1	36.30	-35.11	Bulk sediments		9610 ± 40	10770–11160	10940	TF	RW	790	10150	3	4	Beta-189815	Tanabe et al. (2015)
GS-KTS-1	41.80	-40.61	Bulk sediments		10110 ± 40	11410–11990	11730	TF	RW	1320	10410	-1	8	Beta-189816	Tanabe et al. (2015)
SZ	2.72	-2.11	Plant		970 ± 70	730–1050	870	DF	DA	———	———	———	———	Beta-189146	Tanabe et al. (2015)
SZ	6.82	-6.21	Shell	*Dosinella angulosa* (Philippi)	2910 ± 40	2540–2770	2700	DF	DA	———	———	———	———	Beta-189147	Tanabe et al. (2015)
SZ	10.35	-9.74	Shell	*Dosinella angulosa* (Philippi)	3850 ± 40	3680–3930	3810	DF	DA	———	———	———	———	Beta-189148	Tanabe et al. (2015)
GS-KM-1	7.05	-9.04	Shell	*Raetellops pulchellus* (Adams et Reeve)	2730 ± 40	2320–2610	2440	DF	DA	———	———	———	———	Beta-177895	Tanabe et al. (2015)
GS-KM-1	8.74	-10.73	Shell	*Dosinella angulosa* (Philippi)	3390 ± 40	3140–3360	3260	DF	DA	———	———	———	———	Beta-177896	Tanabe et al. (2015)
GS-KM-1	11.13	-13.12	Shell	*Cryptomya busoensis* Yokoyama	3860 ± 40	3690–3940	3820	DF	DA	———	———	———	———	Beta-177897	Tanabe et al. (2015)
GS-KM-1	13.67	-15.66	Shell	*Dosinella angulosa* (Philippi)	4010 ± 40	3890–4140	4020	DF	DA	———	———	———	———	Beta-177898	Tanabe et al. (2015)
GS-KM-1	18.44	-20.43	Shell	*Dosinella angulosa* (Philippi)	4120 ± 40	4050–4340	4180	DF	DA	———	———	———	———	Beta-177899	Tanabe et al. (2015)
GS-KM-1	21.12	-23.11	Echinoderm		4220 ± 40	4180–4430	4320	DF	DA	———	———	———	———	Beta-177900	Tanabe et al. (2015)
GS-KM-1	25.05	-27.04	Echinoderm		4280 ± 40	4260–4510	4400	DF	DA	———	———	———	———	Beta-177901	Tanabe et al. (2015)
GS-KM-1	28.66	-30.65	Shell	Shell fragments	4710 ± 40	4830–5060	4940	DF	DA	———	———	———	———	Beta-183669	Tanabe et al. (2015)
GS-KM-1	29.10	-31.09	Shell	Shell fragments	5660 ± 40	5940–6180	6060	DF	DA	———	———	———	———	Beta-183670	Tanabe et al. (2015)
GS-KM-1	29.66	-31.65	Shell	Shell fragments	7180 ± 50	7550–7760	7640	DF	DA	———	———	———	———	Beta-177902	Tanabe et al. (2015)
GS-KM-1	30.88	-32.87	Shell	*Potamocorbula* sp.	9140 ± 40	9730–10100	9910	EF	RW	20	9890	-4	85	Beta-183671	Tanabe et al. (2015)
GS-KM-1	31.23	-33.22	Shell	*Corbicula japonica* Prime	9130 ± 50	9690–10100	9890	TF	DA	———	———	———	———	Beta-177903	Tanabe et al. (2015)
GS-KM-1	31.85	-33.84	Shell	*Potamocorbula* sp.	9330 ± 60	9940–10300	10170	TF	DA	———	———	———	———	Beta-183672	Tanabe et al. (2015)
GS-KM-1	34.87	-36.86	Plant		9080 ± 40	10180–10370	10230	MR	DA	———	———	———	———	Beta-183673	Tanabe et al. (2015)
GS-KM-1	36.74	-38.73	Plant		9490 ± 50	10590–11070	10760	MR	DA	———	———	———	———	Beta-177905	Tanabe et al. (2015)
GS-KM-1	39.05	-41.04	Plant		9600 ± 50	10760–11160	10940	MR	DA	———	———	———	———	Beta-177906	Tanabe et al. (2015)
GS-KM-1	42.64	-44.63	Plant		9730 ± 50	10880–11240	11170	MR	DA	———	———	———	———	Beta-177907	Tanabe et al. (2015)
GS-KM-1	45.46	-47.45	Plant		9980 ± 50	11250–11700	11440	MR	DA	———	———	———	———	Beta-177908	Tanabe et al. (2015)
GS-KM-1	50.06	-52.05	Plant		10640 ± 50	12540–12710	12620	MR	DA	———	———	———	———	Beta-177909	Tanabe et al. (2015)
GS-KM-1	54.01	-56.00	Plant		11120 ± 50	12830–13090	13000	MR	RW	110	12890	2	65	Beta-177910	Tanabe et al. (2015)
GS-KM-1	55.43	-57.42	Plant	*Corbicula japonica* Prime	11140 ± 105	12750–13190	12990	MR	DA	———	———	———	———	JNC-5569	Tanabe et al. (2015)
DK	5.03	-4.95	Shell	*Corbicula japonica* Prime	1190 ± 40	650–830	730	DF	RW	320	410	-5	6	Beta-171036	Tanabe et al. (2015)
DK	7.50	-7.42	Shell	*Corbicula japonica* Prime	1090 ± 40	560–720	650	DF	DA	———	———	———	———	Beta-171037	Tanabe et al. (2015)
DK	9.70	-9.62	Shell	Shell fragments	2260 ± 40	1760–1980	1870	DF	DA	———	———	———	———	Beta-171038	Tanabe et al. (2015)
DK	14.58	-14.50	Shell	*Dosinella angulosa* (Philippi)	2610 ± 40	2150–2390	2290	DF	DA	———	———	———	———	Beta-171039	Tanabe et al. (2015)
DK	15.34	-15.26	Shell	*Dosinella angulosa* (Philippi)	3290 ± 40	2980–3250	3120	DF	DA	———	———	———	———	Beta-171040	Tanabe et al. (2015)
DK	19.80	-19.72	Shell	*Dosinella angulosa* (Philippi)	4590 ± 40	4680–4930	4810	DF	DA	———	———	———	———	Beta-171041	Tanabe et al. (2015)
DK	23.15	-23.07	Shell	*Dosinella angulosa* (Philippi)	5830 ± 40	6170–6340	6250	DF	DA	———	———	———	———	Beta-171042	Tanabe et al. (2015)
DK	26.60	-26.52	Shell	*Mactra chinensis* Philippi	6290 ± 40	6640–6860	6750	DF	DA	———	———	———	———	Beta-171043	Tanabe et al. (2015)
DK	27.30	-27.22	Shell	*Dosinella angulosa* (Philippi)	6700 ± 40	7150–7320	7230	DF	DA	———	———	———	———	Beta-171044	Tanabe et al. (2015)
DK	29.30	-29.22	Shell	Shell fragments	7900 ± 40	8280–8450	8360	EF	DA	———	———	———	———	Beta-176663	Tanabe et al. (2015)

DK	33.15	-33.07	Shell	Shell fragments	8520 ± 40	9020–9270	9150	EF	RW	260	8890	-16	42	Beta-176664	Tanabe et al. (2015)
DK	35.25	-35.17	Shell	*Mactra chinensis* Philippi	8480 ± 40	8990–9230	9090	EF	DA	———	———	———	———	Beta-171046	Tanabe et al. (2015)
DK	36.10	-36.02	Shell	Shell fragments	9280 ± 40	9950–10210	10130	TF	DA	———	———	———	———	Beta-171047	Tanabe et al. (2015)
DK	41.50	-41.42	Plant		9410 ± 40	10520–10740	10640	MR	DA	———	———	———	———	Beta-171048	Tanabe et al. (2015)
DK	44.20	-44.12	Plant		9890 ± 40	11220–11390	11280	MR	RW	420	10860	3	5	Beta-171049	Tanabe et al. (2015)
DK	47.25	-47.17	Plant		9980 ± 40	11260–11620	11430	MR	RW	370	11060	0	1	Beta-171050	Tanabe et al. (2015)
DK	49.38	-49.30	Plant		9810 ± 50	11170–11320	11230	MR	DA	———	———	———	———	Beta-171051	Tanabe et al. (2015)
DK	50.09	-50.01	Plant		10400 ± 50	12060–12520	12270	MR	RW	840	11430	0	75	Beta-171052	Tanabe et al. (2015)
DK	53.55	-53.47	Plant		10280 ± 50	11820–12380	12050	MR	DA	———	———	———	———	Beta-171053	Tanabe et al. (2015)
DK	57.20	-57.12	Plant		10770 ± 50	12630–12750	12700	MR	DA	———	———	———	———	Beta-171054	Tanabe et al. (2015)
GS-ISH-1	3.50	-0.81	Plant		230 ± 40	0–430	210	MT	DA	———	———	———	———	TERRA-012109a12	Tanabe et al. (2015)
GS-ISH-1	3.64	-0.95	Shell	*Cerithidea djadjariensis* (Martin)	610 ± 190	0–520	260	MT	DA	———	———	———	———	TERRA-011909a17	Tanabe et al. (2015)
GS-ISH-1	6.54	-3.85	Shell	Shell fragments	780 ± 30	330–490	430	DF	DA	———	———	———	———	TERRA-011909a18	Tanabe et al. (2015)
GS-ISH-1	10.10	-7.41	Plant		2770 ± 40	2780–2960	2870	DF	RW	1840	1030	-7	58	TERRA-012109a15	Tanabe et al. (2015)
GS-ISH-1	15.51	-12.82	Shell	*Dosinella angulosa* (Philippi)	2150 ± 30	1630–1830	1740	DF	DA	———	———	———	———	TERRA-011909a20	Tanabe et al. (2015)
GS-ISH-1	18.80	-16.11	Shell	*Dosinella angulosa* (Philippi)	2880 ± 30	2530–2740	2670	DF	DA	———	———	———	———	TERRA-011909a21	Tanabe et al. (2015)
GS-ISH-1	22.61	-19.92	Shell	*Dosinella angulosa* (Philippi)	3480 ± 40	3240–3460	3360	DF	DA	———	———	———	———	TERRA-011909a22	Tanabe et al. (2015)
GS-ISH-1	25.20	-22.51	Shell	*Dosinella angulosa* (Philippi)	5790 ± 30	6140–6280	6220	DF	DA	———	———	———	———	TERRA-011909a23	Tanabe et al. (2015)
GS-ISH-1	27.34	-24.65	Shell	Shell fragments	8020 ± 60	8360–8600	8480	EF	RW	1050	7430	-25	14	TERRA-011909a24	Tanabe et al. (2015)
GS-ISH-1	28.50	-25.81	Shell	*Mitrella yabei* (Nomura)	8150 ± 40	8510–8780	8620	EF	RW	630	7990	-22	38	TERRA-011909a27	Tanabe et al. (2015)
GS-ISH-1	29.15	-26.46	Plant		7840 ± 30	8210–8380	8310	EF	DA	———	———	———	———	TERRA-012109a16	Tanabe et al. (2015)
GS-ISH-1	29.36	-26.67	Shell	Shell fragments	8810 ± 30	9420–9520	9480	EF	RW	950	8530	-19	52	TERRA-011909a28	Tanabe et al. (2015)
GS-ISH-1	29.40	-26.71	Echinoderm		8510 ± 80	8960–9370	9140	EF	DA	———	———	———	———	TERRA-012609a06	Tanabe et al. (2015)
GS-ISH-1	30.30	-27.61	Shell	Shell fragments	8640 ± 40	9180–9430	9320	EF	DA	———	———	———	———	TERRA-011909a29	Tanabe et al. (2015)
GS-ISH-1	34.90	-32.21	Shell	Shell fragments	8720 ± 40	9290–9480	9400	EF	DA	———	———	———	———	TERRA-011909a30	Tanabe et al. (2015)
GS-ISH-1	37.94	-35.25	Shell	*Batillaria multiformis* (Lischke)	9160 ± 30	9800–10110	9950	EF	DA	———	———	———	———	TERRA-011909a32	Tanabe et al. (2015)
GS-ISH-1	39.16	-36.47	Shell	*Potamocorbula* sp.	9470 ± 80	10170–10520	10330	EF	RW	320	10010	-7	2	TERRA-012609a07	Tanabe et al. (2015)
GS-ISH-1	40.95	-38.26	Shell	*Potamocorbula* sp.	9320 ± 50	9960–10260	10160	EF	DA	———	———	———	———	TERRA-011909a33	Tanabe et al. (2015)
GS-ISH-1	45.23	-42.54	Plant		9100 ± 50	10190–10400	10250	EF	DA	———	———	———	———	Beta-289597	Tanabe et al. (2015)
GS-ISH-1	46.61	-43.92	Shell	*Potamocorbula* sp.	9840 ± 40	10640–10960	10770	EF	DA	———	———	———	———	TERRA-011909a35	Tanabe et al. (2015)
GS-ISH-1	47.51	-44.82	Shell	*Corbicula* sp.	10020 ± 40	10850–11150	11020	EF	RW	160	10860	2	54	TERRA-011909a36	Tanabe et al. (2015)
GS-ISH-1	48.55	-45.86	Shell	Shell fragments	9950 ± 60	10730–11110	10920	EF	DA	———	———	———	———	TERRA-011909a39	Tanabe et al. (2015)
GS-ISH-1	50.75	-48.06	Plant		9980 ± 50	11250–11700	11440	MR	DA	———	———	———	———	Beta-289599	Tanabe et al. (2015)
GS-ISH-1	52.52	-49.83	Plant		10480 ± 50	12140–12570	12440	MR	DA	———	———	———	———	Beta-289600	Tanabe et al. (2015)
GS-ISH-1	53.87	-51.18	Plant		10610 ± 50	12430–12700	12600	MR	DA	———	———	———	———	Beta-289601	Tanabe et al. (2015)
GS-ISH-1	54.13	-51.44	Plant		11000 ± 50	12730–13000	12860	MR	DA	———	———	———	———	Beta-289602	Tanabe et al. (2015)
GS-ISH-1	55.57	-52.88	Plant		11590 ± 50	13300–13550	13420	MR	RW	160	13260	7	19	Beta-289603	Tanabe et al. (2015)
GS-ISH-1	55.63	-52.94	Plant		11220 ± 40	13020–13160	13090	MR	DA	———	———	———	———	TERRA-012109a22	Tanabe et al. (2015)
GS-ISH-1	56.05	-53.36	Plant		12210 ± 60	13860–14330	14110	MR	DA	———	———	———	———	Beta-289604	Tanabe et al. (2015)
GS-ISH-1	58.58	-55.89	Plant		> 43500	———	———	SH	Base	———	———	———	———	Beta-289605	Tanabe et al. (2015)
GS-ISH-1	59.89	-57.20	Plant		> 43500	———	———	SH	Base	———	———	———	———	Beta-289606	Tanabe et al. (2015)
GS-KSM-1	15.95	-7.97	Shell	*Macoma contabulata* (Deshayes)	930 ± 30	490–610	530	DF	DA	———	———	———	———	IAAA-82888	Tanabe et al. (2015)
GS-KSM-1	20.40	-12.42	Shell	*Veremolpa micra* (Pilsbry)	1870 ± 30	1330–1510	1420	DF	DA	———	———	———	———	IAAA-82889	Tanabe et al. (2015)
GS-KSM-1	22.20	-14.22	Shell	*Dosinella angulosa* (Philippi)	2080 ± 30	1550–1750	1650	DF	DA	———	———	———	———	IAAA-82890	Tanabe et al. (2015)
GS-KSM-1	24.85	-16.87	Shell	*Dosinella angulosa* (Philippi)	4480 ± 40	4540–4800	4680	DF	DA	———	———	———	———	IAAA-82891	Tanabe et al. (2015)
GS-KSM-1	26.70	-18.72	Shell	*Dosinella angulosa* (Philippi)	5990 ± 40	6290–6500	6400	DF	DA	———	———	———	———	IAAA-82892	Tanabe et al. (2015)
GS-KSM-1	27.58	-19.60	Shell	*Dosinella angulosa* (Philippi)	6150 ± 40	6470–6690	6580	DF	DA	———	———	———	———	IAAA-82893	Tanabe et al. (2015)
GS-KSM-1	28.71	-20.73	Shell	Shell fragments	6410 ± 40	6770–7000	6890	DF	DA	———	———	———	———	IAAA-82894	Tanabe et al. (2015)
GS-KSM-1	30.50	-22.52	Shell	*Barnea* sp.	7450 ± 40	7820–8000	7910	EF	DA	———	———	———	———	IAAA-82895	Tanabe et al. (2015)
GS-KSM-1	32.32	-24.34	Shell	*Raetellops pulchellus* (Adams et Reeve)	7890 ± 40	8260–8440	8360	EF	DA	———	———	———	———	IAAA-82896	Tanabe et al. (2015)
GS-KSM-1	34.45	-26.47	Shell	Shell fragments	8090 ± 40	8420–8660	8550	EF	DA	———	———	———	———	IAAA-82897	Tanabe et al. (2015)
GS-KSM-1	35.95	-27.97	Shell	*Potamocorbula* sp.	8640 ± 50	9150–9430	9310	EF	RW	420	8890	-11	10	IAAA-82898	Tanabe et al. (2015)
GS-KSM-1	37.30	-29.32	Shell	*Potamocorbula* sp.	8590 ± 40	9110–9380	9250	EF	DA	———	———	———	———	IAAA-82899	Tanabe et al. (2015)
GS-KSM-1	39.25	-31.27	Shell	*Potamocorbula* sp.	8860 ± 40	9430–9600	9510	EF	DA	———	———	———	———	IAAA-82900	Tanabe et al. (2015)
GS-KSM-1	40.30	-32.32	Shell	*Potamocorbula* sp.	8960 ± 50	9500–9780	9620	EF	DA	———	———	———	———	IAAA-82901	Tanabe et al. (2015)
GS-KSM-1	42.80	-34.82	Shell	*Potamocorbula* sp.	9260 ± 50	9910–10200	10090	EF	DA	———	———	———	———	IAAA-82902	Tanabe et al. (2015)
GS-KSM-1	43.55	-35.57	Shell	*Potamocorbula* sp.	9340 ± 50	10020–10300	10180	EF	DA	———	———	———	———	IAAA-82903	Tanabe et al. (2015)
GS-KSM-1	46.50	-38.52	Plant		9340 ± 50	10410–10700	10560	TF	DA	———	———	———	———	IAAA-82904	Tanabe et al. (2015)
GS-KSM-1	52.30	-44.32	Plant		9720 ± 50	10870–11240	11160	TF	RW	320	10840	3	10	IAAA-82905	Tanabe et al. (2015)
GS-KSM-1	53.50	-45.52	Plant		9560 ± 40	10730–11090	10930	MR	DA	———	———	———	———	IAAA-82906	Tanabe et al. (2015)
GS-KSM-1	56.65	-48.67	Plant		10000 ± 50	11270–11710	11480	MR	DA	———	———	———	———	IAAA-82907	Tanabe et al. (2015)
GS-KSM-1	59.85	-51.87	Plant		10100 ± 50	11400–11970	11700	MR	DA	———	———	———	———	IAAA-82908	Tanabe et al. (2015)
GS-KSM-1	62.80	-54.82	Plant		10380 ± 50	12030–12510	12250	MR	DA	———	———	———	———	IAAA-82909	Tanabe et al. (2015)
GS-KSM-1	65.15	-57.17	Plant		11050 ± 50	12780–13060	12920	MR	DA	———	———	———	———	IAAA-82910	Tanabe et al. (2015)
GS-KSM-1	68.25	-60.27	Plant		11520 ± 50	13270–13460	13360	MR	DA	———	———	———	———	IAAA-82911	Tanabe et al. (2015)
GS-KWS-1	15.26	-6.71	Plant		210 ±20	0–300	170	DF	DA	———	———	———	———	IAAA-190773	Komatsubara et al. (2020)
GS-KWS-1	18.62	-10.07	Shell	*Meretrix lusiria* (Röding)	1100 ±20	620–700	660	DF	RW	250	410	-10	50	IAAA-190774	Komatsubara et al. (2020)
GS-KWS-1	22.69	-14.14	Plant		1080 ±20	940–1050	980	DF	RW	330	650	-14	10	IAAA-190775	Komatsubara et al. (2020)
GS-KWS-1	24.73	-16.18	Plant		870 ±20	730–900	770	DF	DA	———	———	———	———	IAAA-190776	Komatsubara et al. (2020)
GS-KWS-1	28.62	-20.07	Shell	*Dosinella angulosa* (Philippi)	1720 ±20	1230–1330	1280	DF	DA	———	———	———	———	IAAA-190777	Komatsubara et al. (2020)
GS-KWS-1	30.80	-22.25	Shell	*Dosinella angulosa* (Philippi)	2360 ±20	1900–2070	1980	DF	DA	———	———	———	———	IAAA-190778	Komatsubara et al. (2020)
GS-KWS-1	33.05	-24.50	Shell	*Dosinella angulosa* (Philippi)	2780 ±20	2400–2660	2520	DF	DA	———	———	———	———	IAAA-190779	Komatsubara et al. (2020)
GS-KWS-1	35.96	-27.41	Shell	*Dosinella angulosa* (Philippi)	3160 ±20	2860–3040	2940	DF	DA	———	———	———	———	IAAA-190780	Komatsubara et al. (2020)
GS-KWS-1	38.78	-30.23	Shell	*Macoma* sp.	3420 ±30	3200–3380	3290	DF	DA	———	———	———	———	IAAA-190781	Komatsubara et al. (2020)
GS-KWS-1	42.49	-33.94	Shell	*Barnea* sp.	3700 ±30	3520–3710	3620	DF	DA	———	———	———	———	IAAA-190782	Komatsubara et al. (2020)
GS-KWS-1	46.87	-38.32	Shell	*Ringiculina doliaris* (Gould)	4050 ±30	3960–4200	4080	DF	DA	———	———	———	———	IAAA-190783	Komatsubara et al. (2020)
GS-KWS-1	48.04	-39.49	Shell	*Potamocorbula* sp.	9210 ±30	9900–10140	10030	EF	DA	———	———	———	———	IAAA-190784	Komatsubara et al. (2020)

GS-KWS-1	54.27	-45.72	Plant		9650 ±40	10790–11190	11070	MR	DA	———	———	———	———	IAAA-190785	Komatsubara et al. (2020)
GS-KWS-1	62.06	-53.51	Plant		10310 ±40	11960–12380	12110	MR	DA	———	———	———	———	IAAA-190786	Komatsubara et al. (2020)
GS-KWS-1	65.24	-56.69	Plant		10470 ±40	12140–12560	12430	MR	DA	———	———	———	———	IAAA-190787	Komatsubara et al. (2020)
GS-KWS-1	71.17	-62.62	Plant		11550 ±40	13300–13470	13390	MR	DA	———	———	———	———	IAAA-190788	Komatsubara et al. (2020)
GS-KWS-1	75.33	-66.78	Plant		11630 ±40	13380–13570	13460	MR	DA	———	———	———	———	IAAA-190789	Komatsubara et al. (2020)
GS-KWS-1	77.13	-68.58	Plant		12130 ±40	13830–14140	14010	MR	DA	———	———	———	———	IAAA-190790	Komatsubara et al. (2020)
GS-KWS-1	78.56	-70.01	Plant		12450 ±40	14250–14930	14590	MR	DA	———	———	———	———	IAAA-190791	Komatsubara et al. (2020)
GS-KSO-1	3.72	6.05	Plant		230 ± 40	0–430	210	MF	RW	200	10	6	50	Beta-290517	Komatsubara et al. (2017)
GS-KSO-1	4.23	5.54	Plant		150 ± 40	0–280	150	MF	DA	———	———	———	———	Beta-290518	Komatsubara et al. (2017)
GS-KSO-1	8.11	1.66	Plant		3940 ± 30	4260–4510	4390	MF	DA	———	———	———	———	Beta-291589	Komatsubara et al. (2017)
GS-KSO-1	8.72	1.05	Plant		5140 ± 40	5750–5990	5900	DF	DA	———	———	———	———	Beta-290520	Komatsubara et al. (2017)
GS-KSO-1	8.95	0.82	Plant		5220 ± 40	5910–6180	5970	DF	DA	———	———	———	———	Beta-292226	Komatsubara et al. (2017)
GS-KSO-1	9.84	-0.07	Wood		5890 ± 40	6580–6830	6710	DF	DA	———	———	———	———	Beta-290521	Komatsubara et al. (2017)
GS-KSO-1	10.87	-1.10	Plant		6290 ± 40	7160–7320	7220	DF	RW	420	6800	-4	0	Beta-290522	Komatsubara et al. (2017)
GS-KSO-1	11.64	-1.87	Plant		5930 ± 40	6660–6880	6750	DF	DA	———	———	———	———	Beta-290523	Komatsubara et al. (2017)
GS-KSO-1	12.67	-2.90	Plant		6930 ± 40	7680–7850	7760	TF	DA	———	———	———	———	Beta-290524	Komatsubara et al. (2017)
GS-KSO-1	13.15	-3.38	Plant		7060 ± 40	7800–7970	7890	TF	DA	———	———	———	———	Beta-290525	Komatsubara et al. (2017)
GS-KSO-1	15.80	-6.03	Wood		7090 ± 40	7840–8000	7920	DF	DA	———	———	———	———	Beta-290526	Komatsubara et al. (2017)
GS-KSO-1	16.96	-7.19	Plant		7140 ± 50	7850–8040	7970	DF	DA	———	———	———	———	Beta-292227	Komatsubara et al. (2017)
GS-KSO-1	17.27	-7.50	Plant		7200 ± 50	7940–8160	8010	DF	DA	———	———	———	———	Beta-290527	Komatsubara et al. (2017)
GS-KSO-1	17.93	-8.16	Plant		13550 ± 70	16080–16590	16320	DF	RW	8200	———	———	———	Beta-292228	Komatsubara et al. (2017)
GS-KSO-1	18.19	-8.42	Plant		7350 ± 40	8030–8310	8160	EF	DA	———	———	———	———	Beta-292229	Komatsubara et al. (2017)
GS-KSO-1	19.21	-9.44	Plant		7460 ± 50	8190–8370	8280	EF	DA	———	———	———	———	Beta-290528	Komatsubara et al. (2017)
GS-KSO-1	21.64	-11.87	Plant		7940 ± 50	8630–8990	8800	TF	DA	———	———	———	———	Beta-290529	Komatsubara et al. (2017)
GS-KSO-1	24.92	-15.15	Plant		8000 ± 50	8650–9010	8870	TF	RW	50	8820	-1	10	Beta-290530	Komatsubara et al. (2017)
GS-KSO-1	26.50	-16.73	Plant		8110 ± 50	8790–9260	9060	TF	RW	160	8900	0	0	Beta-290531	Komatsubara et al. (2017)
GS-KSO-1	27.35	-17.58	Wood		7990 ± 50	8650–9010	8860	MR	DA	———	———	———	———	Beta-292230	Komatsubara et al. (2017)
GS-KSO-1	27.72	-17.95	Plant		8260 ± 50	9040–9420	9250	MR	DA	———	———	———	———	Beta-292678	Komatsubara et al. (2017)
GS-KSO-1	30.88	-21.11	Plant		8420 ± 50	9310–9530	9450	MR	DA	———	———	———	———	Beta-290533	Komatsubara et al. (2017)
GS-SSS-1	1.87	2.76	Plant		1990 ± 40	1830–2040	1940	MF	DA	———	———	———	———	TERRA-031710a11	Komatsubara et al. (2017)
GS-SSS-1	3.55	1.08	Plant		5620 ± 60	6290–6530	6400	MF	DA	———	———	———	———	TERRA-031710a12	Komatsubara et al. (2017)
GS-SSS-1	5.20	-0.57	Plant		5760 ± 30	6480–6650	6560	MF	DA	———	———	———	———	TERRA-020110a12	Komatsubara et al. (2017)
GS-SSS-1	6.85	-2.22	Plant		6390 ± 50	7180–7430	7330	DF	DA	———	———	———	———	TERRA-020110a16	Komatsubara et al. (2017)
GS-SSS-1	7.50	-2.87	Plant		7130 ± 30	7880–8010	7960	DF	RW	840	7120	-6	75	TERRA-031710a14	Komatsubara et al. (2017)
GS-SSS-1	9.06	-4.43	Plant		6480 ± 40	7310–7470	7380	DF	DA	———	———	———	———	TERRA-031710a13	Komatsubara et al. (2017)
GS-SSS-1	10.52	-5.89	Plant		6620 ± 60	7430–7590	7510	DF	DA	———	———	———	———	TERRA-040510b02	Komatsubara et al. (2017)
GS-SSS-1	11.33	-6.70	Plant		6810 ± 30	7590–7680	7650	DF	RW	110	7540	-7	45	TERRA-031710a15	Komatsubara et al. (2017)
GS-SSS-1	12.42	-7.79	Plant		6700 ± 80	7440–7680	7570	DF	DA	———	———	———	———	TERRA-040510b01	Komatsubara et al. (2017)
GS-SSS-1	15.46	-10.83	Plant		7050 ± 130	7660–8160	7870	DF	DA	———	———	———	———	TERRA-040510b23	Komatsubara et al. (2017)
GS-SSS-1	17.52	-12.89	Plant		7430 ± 40	8180–8340	8260	DF	RW	320	7940	-10	45	TERRA-020110a13	Komatsubara et al. (2017)
GS-SSS-1	19.41	-14.78	Plant		7080 ± 40	7830–7980	7910	DF	DA	———	———	———	———	TERRA-040510b09	Komatsubara et al. (2017)
GS-SSS-1	20.47	-15.84	Plant		9750 ± 50	10900–11250	11190	EF	RW	2790	8400	-8	0	TERRA-040510b22	Komatsubara et al. (2017)
GS-SSS-1	21.55	-16.92	Plant		7760 ± 40	8440–8600	8540	EF	DA	———	———	———	———	TERRA-020110a14	Komatsubara et al. (2017)
GS-SSS-1	22.88	-18.25	Plant		7880 ± 50	8560–8980	8700	EF	DA	———	———	———	———	TERRA-040610b02	Komatsubara et al. (2017)
GS-SSS-1	24.30	-19.67	Plant		8090 ± 40	8790–9130	9020	EF	DA	———	———	———	———	TERRA-020110a17	Komatsubara et al. (2017)
GS-SSS-1	25.65	-21.02	Plant		8140 ± 60	8820–9290	9090	TF	DA	———	———	———	———	TERRA-040510b10	Komatsubara et al. (2017)
GS-SSS-1	27.61	-22.98	Plant		8240 ± 70	9030–9410	9220	TF	DA	———	———	———	———	TERRA-040510b24	Komatsubara et al. (2017)
GS-SSS-1	45.30	-40.67	Plant		42970 ± 380	———	———	SH	Base	———	———	———	———	TERRA-020110a15	Komatsubara et al. (2017)
GS-TKT-1	2.92	0.18	Plant		4020 ± 40	4410–4780	4490	MF	DA	———	———	———	———	Beta-251336	Komatsubara et al. (2017)
GS-TKT-1	4.30	-1.20	Plant		4270 ± 40	4650–4960	4850	MF	DA	———	———	———	———	TERRA-111809a12	Komatsubara et al. (2017)
GS-TKT-1	7.43	-4.33	Shell	*Potamocorbula* sp.	6710 ± 40	7150–7330	7240	DF	RW	140	7100	-7	90	TERRA-111809a26	Komatsubara et al. (2017)
GS-TKT-1	7.60	-4.50	Plant		6210 ± 30	7010–7240	7100	DF	DA	———	———	———	———	TERRA-111809a11	Komatsubara et al. (2017)
GS-TKT-1	7.60	-4.50	Shell	*Potamocorbula* sp.	6840 ± 30	7290–7420	7360	DF	RW	260	7100	-8	75	TERRA-111809a22	Komatsubara et al. (2017)
GS-TKT-1	7.85	-4.75	Shell	*Potamocorbula* sp.	6720 ± 50	7150–7370	7250	DF	RW	150	7100	-8	60	TERRA-111809a21	Komatsubara et al. (2017)
GS-TKT-1	8.05	-4.95	Shell	*Potamocorbula* sp.	6840 ± 30	7290–7420	7360	DF	RW	240	7120	-8	80	TERRA-111809a30	Komatsubara et al. (2017)
GS-TKT-1	8.40	-5.30	Shell	*Potamocorbula* sp.	6720 ± 30	7160–7320	7250	DF	RW	120	7130	-8	75	TERRA-111809a29	Komatsubara et al. (2017)
GS-TKT-1	8.80	-5.70	Plant		6250 ± 40	7020–7260	7190	DF	RW	50	7140	-9	35	Beta-251337	Komatsubara et al. (2017)
GS-TKT-1	8.85	-5.75	Shell	*Potamocorbula* sp.	6800 ± 60	7200–7430	7330	DF	RW	190	7140	-9	35	TERRA-113009a02	Komatsubara et al. (2017)
GS-TKT-1	9.39	-6.29	Plant		6230 ± 50	7000–7260	7150	DF	DA	———	———	———	———	TERRA-111809a10	Komatsubara et al. (2017)
GS-TKT-1	9.79	-6.69	Shell	*Potamocorbula* sp.	6940 ± 30	7390–7520	7450	DF	RW	290	7160	-10	40	TERRA-111809a24	Komatsubara et al. (2017)
GS-TKT-1	11.28	-8.18	Shell	*Potamocorbula* sp.	6840 ± 40	7270–7430	7360	DF	RW	160	7200	-10	35	TERRA-112009a12	Komatsubara et al. (2017)
GS-TKT-1	11.28	-8.18	Shell	*Retusa (Decolifer) insignis* (Pilsbry)	6690 ± 40	7140–7310	7220	DF	RW	20	7200	-10	35	TERRA-111809a19	Komatsubara et al. (2017)
GS-TKT-1	11.32	-8.22	Plant		6370 ± 40	7180–7420	7310	DF	RW	110	7200	-10	35	Beta-251792	Komatsubara et al. (2017)
GS-TKT-1	11.71	-8.61	Shell	Shell fragments	6760 ± 40	7200–7390	7290	DF	RW	80	7210	-11	50	TERRA-111809a23	Komatsubara et al. (2017)
GS-TKT-1	11.75	-8.65	Shell	*Assiminea japonica* Martens	6840 ± 30	7290–7420	7360	DF	RW	150	7210	-11	50	TERRA-120209a15	Komatsubara et al. (2017)
GS-TKT-1	11.82	-8.72	Shell	*Potamocorbula* sp.	6950 ± 40	7390–7550	7460	DF	RW	240	7220	-11	55	TERRA-111809a28	Komatsubara et al. (2017)
GS-TKT-1	11.96	-8.86	Shell	*Potamocorbula* sp.	6970 ± 30	7410–7540	7470	DF	RW	240	7230	-11	55	TERRA-111809a25	Komatsubara et al. (2017)
GS-TKT-1	12.03	-8.93	Shell	*Mactra chinensis* Philippi	6950 ± 40	7390–7550	7460	DF	RW	240	7220	-11	55	TERRA-111809a20	Komatsubara et al. (2017)
GS-TKT-1	12.28	-9.18	Shell	Shell fragments	6690 ± 30	7150–7290	7220	DF	DA	———	———	———	———	TERRA-111809a18	Komatsubara et al. (2017)
GS-TKT-1	13.70	-10.60	Shell	*Potamocorbula* sp.	7460 ± 40	7830–8000	7920	DF	RW	660	7260	-13	20	TERRA-111809a09	Komatsubara et al. (2017)
GS-TKT-1	13.70	-10.60	Shell	*Potamocorbula* sp.	7060 ± 30	7470–7610	7540	DF	RW	280	7260	-13	20	TERRA-111809a17	Komatsubara et al. (2017)
GS-TKT-1	13.80	-10.70	Shell	*Potamocorbula* sp.	7100 ± 30	7510–7650	7580	DF	RW	320	7260	-13	20	TERRA-111809a16	Komatsubara et al. (2017)
GS-TKT-1	14.01	-10.91	Wood		6420 ± 50	7270–7430	7350	DF	RW	80	7270	-13	20	Beta-251339	Komatsubara et al. (2017)
GS-TKT-1	14.10	-11.00	Shell	*Potamocorbula* sp.	6880 ± 40	7300–7470	7390	DF	RW	120	7270	-13	20	TERRA-113009a05	Komatsubara et al. (2017)
GS-TKT-1	14.80	-11.70	Shell	Shell fragments	6850 ± 40	7270–7440	7370	DF	RW	80	7290	-14	15	TERRA-113009a03	Komatsubara et al. (2017)
GS-TKT-1	14.90	-11.80	Shell	*Potamocorbula* sp.	6980 ± 40	7410–7560	7480	DF	RW	190	7290	-14	15	TERRA-112709a01	Komatsubara et al. (2017)
GS-TKT-1	15.54	-12.44	Shell	*Potamocorbula* sp.	7260 ± 50	7610–7840	7720	DF	RW	420	7300	-14	10	TERRA-113009a04	Komatsubara et al. (2017)

GS-TKT-1	15.78	-12.68	Shell	Shell fragments	7340 ± 30	7710–7900	7810	DF	RW	500	7310	-15	5	TERRA-111809a27	Komatsubara et al. (2017)
GS-TKT-1	16.40	-13.30	Plant		6390 ± 30	7270–7420	7320	DF	DA	———	———	———	———	TERRA-111809a08	Komatsubara et al. (2017)
GS-TKT-1	19.57	-16.47	Shell	*Crassostrea gigas* (Thunberg)	8490 ± 120	8770–9420	9110	DF	RW	1190	7920	-13	25	TERRA-113009a01	Komatsubara et al. (2017)
GS-TKT-1	19.60	-16.50	Plant		7780 ± 50	8430–8640	8560	DF	RW	670	7890	-14	25	Beta-251340	Komatsubara et al. (2017)
GS-TKT-1	21.05	-17.95	Plant		7340 ± 40	8020–8300	8140	EF	DA	———	———	———	———	TERRA-111809a07	Komatsubara et al. (2017)
GS-TKT-1	23.95	-20.85	Plant		8090 ± 70	8730–9260	9020	TF	DA	———	———	———	———	TERRA-112009a19	Komatsubara et al. (2017)
GS-TKT-1	24.77	-21.67	Plant		13730 ± 60	16330–16860	16580	TF	RW	7780	———	———	———	Beta-251341	Komatsubara et al. (2017)
GS-TKT-1	25.65	-22.55	Plant		8390 ± 50	9290–9520	9420	TF	RW	360	9060	-3	10	Beta-251342	Komatsubara et al. (2017)
GS-TKT-1	26.56	-23.46	Plant		8120 ± 40	8990–9240	9060	TF	DA	———	———	———	———	TERRA-112709a01	Komatsubara et al. (2017)
GS-TKT-1	27.71	-24.61	Plant		8530 ± 40	9480–9550	9520	TF	RW	200	9320	0	0	TERRA-111809a15	Komatsubara et al. (2017)
GS-TKT-1	28.24	-25.14	Plant		8350 ± 50	9150–9490	9370	TF	DA	———	———	———	———	Beta-251343	Komatsubara et al. (2017)
GS-TKT-1	31.02	-27.92	Plant		8730 ± 60	9540–10110	9710	TF	DA	———	———	———	———	Beta-252173	Komatsubara et al. (2017)
GS-TKT-1	35.50	-32.40	Plant		9200 ± 50	10250–10500	10360	MR	DA	———	———	———	———	TERRA-111609a29	Komatsubara et al. (2017)
GS-TKT-1	36.80	-33.70	Plant		9330 ± 40	10420–10660	10540	MR	DA	———	———	———	———	TERRA-111809a14	Komatsubara et al. (2017)
GS-TKT-1	37.00	-33.90	Plant		9520 ± 40	10680–11080	10850	MR	DA	———	———	———	———	TERRA-111809a13	Komatsubara et al. (2017)
GS-FB-2	6.47	-2.91	Shell	*Mactra chinensis* Philippi	1320 ± 20	790–920	870	MF	DA	———	———	———	———	IAAA-141118	Kazaoka et al. (2018)
GS-FB-2	9.23	-5.67	Plant		120 ± 20	10–270	110	MF	DA	———	———	———	———	IAAA-133058	Kazaoka et al. (2018)
GS-FB-2	10.84	-7.28	Plant		1890 ± 20	1740–1890	1850	MF	DA	———	———	———	———	IAAA-133059	Kazaoka et al. (2018)
GS-FB-2	13.66	-10.10	Plant		1910 ± 20	1820–1900	1860	DF	DA	———	———	———	———	IAAA-133060	Kazaoka et al. (2018)
GS-FB-2	17.86	-14.30	Shell	*Dosinella angulosa* (Philippi)	3260 ± 20	2990–3170	3090	DF	DA	———	———	———	———	IAAA-140336	Kazaoka et al. (2018)
GS-FB-2	19.80	-16.24	Shell	*Dosinella angulosa* (Philippi)	5850 ± 30	6190–6340	6270	DF	DA	———	———	———	———	IAAA-140337	Kazaoka et al. (2018)
GS-FB-2	21.37	-17.81	Shell	*Solen* sp.	8410 ± 30	8930–9110	9010	TF	DA	———	———	———	———	IAAA-140338	Kazaoka et al. (2018)
GS-FB-2	28.06	-24.50	Plant		42250 ± 770	———	———	EF	RW	———	———	———	———	IAAA-133061	Kazaoka et al. (2018)
GS-FB-2	30.44	-26.88	Plant		8330 ± 30	9270–9450	9360	EF	DA	———	———	———	———	IAAA-133062	Kazaoka et al. (2018)
GS-FB-2	30.65	-27.09	Shell	*Tegillarca granosa* (Linnaeus)	8840 ± 30	9440–9540	9490	EF	DA	———	———	———	———	IAAA-140339	Kazaoka et al. (2018)
GS-FB-2	35.55	-31.99	Plant		8810 ± 30	9690–10120	9840	EF	DA	———	———	———	———	IAAA-133063	Kazaoka et al. (2018)
GS-FB-2	36.64	-33.08	Plant		9190 ± 30	10250–10430	10340	MR	DA	———	———	———	———	IAAA-133064	Kazaoka et al. (2018)
GS-FB-3	6.83	-3.35	Plant		1420 ± 20	1300–1350	1320	MF	DA	———	———	———	———	IAAA-141119	Kazaoka et al. (2018)
GS-FB-3	10.65	-7.17	Plant		750 ± 20	670–720	680	MF	DA	———	———	———	———	IAAA-133065	Kazaoka et al. (2018)
GS-FB-3	10.92	-7.44	Plant		1070 ± 20	930–1050	970	MF	DA	———	———	———	———	IAAA-133066	Kazaoka et al. (2018)
GS-FB-4	7.20	-3.11	Shell	*Meretrix lusiria* (Röding)	1260 ± 20	730–880	810	DF	DA	———	———	———	———	IAAA-150481	Kazaoka et al. (2018)
GS-FB-4	12.45	-8.36	Shell	*Raetellops pulchellus* (Adams et Reeve)	2880 ± 30	2530–2740	2670	DF	DA	———	———	———	———	IAAA-150482	Kazaoka et al. (2018)
GS-FB-4	14.10	-10.01	Shell	*Veremolpa micra* (Pilsbry)	4120 ± 30	4070–4290	4180	DF	DA	———	———	———	———	IAAA-153563	Kazaoka et al. (2018)
GS-FB-4	15.24	-11.15	Shell	*Meretrix lusiria* (Röding)	8030 ± 30	8400–8560	8480	EF	DA	———	———	———	———	IAAA-150483	Kazaoka et al. (2018)
GS-FB-4	17.13	-13.04	Shell	*Mactra chinensis* Philippi	8260 ± 30	8650–8950	8800	TF	DA	———	———	———	———	IAAA-150484	Kazaoka et al. (2018)
GS-FB-4	23.33	-19.24	Wood		7980 ± 30	8720–9000	8870	EF	DA	———	———	———	———	IAAA-150485	Kazaoka et al. (2018)
GS-NS-1	6.74	-3.84	Shell	*Batillaria cumingii* (Crosse)	1420 ± 20	910–1030	960	DF	DA	———	———	———	———	IAAA-150486	Kazaoka et al. (2018)
GS-NS-1	8.08	-5.18	Shell	*Dentalium (Paradentalium) octangulatum* Donovan	2880 ± 30	2530–2740	2670	DF	DA	———	———	———	———	IAAA-153564	Kazaoka et al. (2018)
GS-NS-1	9.70	-6.80	Shell	*Meretrix lusiria* (Röding)	7790 ± 30	8180–8330	8260	TF	DA	———	———	———	———	IAAA-150487	Kazaoka et al. (2018)
GS-NS-1	14.88	-11.98	Shell	*Dosinella angulosa* (Philippi)	8240 ± 30	8630–8920	8760	EF	RW	150	———	———	———	IAAA-150488	Kazaoka et al. (2018)
GS-NS-1	16.96	-14.06	Wood		7880 ± 30	8590–8850	8670	EF	DA	———	———	———	———	IAAA-150489	Kazaoka et al. (2018)
GS-CB-2	8.68	-4.32	Shell	*Scapharca kagoshimensis* (Tokunaga)	460 ± 20	0–130	60	DF	DA	———	———	———	———	IAAA-142446	Kazaoka et al. (2018)
GS-CB-2	9.17	-4.81	Shell	*Nitidotellina hokkaidoensis* (Habe)	820 ± 20	420–500	460	DF	DA	———	———	———	———	IAAA-142447	Kazaoka et al. (2018)
GS-CB-2	10.82	-6.46	Shell	*Veremolpa micra* (Pilsbry)	2150 ± 30	1630–1830	1740	DF	DA	———	———	———	———	IAAA-142448	Kazaoka et al. (2018)
GS-CB-2	11.70	-7.34	Shell	Shell fragments	2300 ± 30	1820–1990	1910	DF	DA	———	———	———	———	IAAA-142449	Kazaoka et al. (2018)
GS-CB-2	12.57	-8.21	Shell	*Moerella* sp.	8040 ± 30	8410–8570	8490	TF	DA	———	———	———	———	IAAA-142450	Kazaoka et al. (2018)
GS-CB-2	13.48	-9.12	Shell	*Mactra veneriformis* Deshayes in Reeve	8140 ± 30	8520–8730	8610	TF	DA	———	———	———	———	IAAA-142451	Kazaoka et al. (2018)
GS-CB-2	17.30	-12.94	Shell	*Pecten albicans* (Schröter)	47730 ± 580	———	———	SH	Base	———	———	———	———	IAAA-142452	Kazaoka et al. (2018)
GS-CB-3	6.21	-0.83	Shell	*Mactra chinensis* Philippi	7070 ± 30	7480–7620	7550	TF	DA	———	———	———	———	IAAA-153565	Kazaoka et al. (2018)
GS-CB-3	8.30	-2.92	Shell	*Mactra chinensis* Philippi	7390 ± 30	7780–7930	7860	TF	DA	———	———	———	———	IAAA-150476	Kazaoka et al. (2018)
GS-CB-3	13.23	-7.85	Shell	*Borniopsis tsurumaru* Habe	7770 ± 30	8160–8320	8240	EF	DA	———	———	———	———	IAAA-142455	Kazaoka et al. (2018)
GS-CB-3	14.54	-9.16	Shell	*Reticunassa festiva* (Powy)	7890 ± 30	8290–8420	8360	EF	DA	———	———	———	———	IAAA-142456	Kazaoka et al. (2018)
GS-CB-3	15.45	-10.07	Crab		8110 ± 30	8470–8680	8570	EF	RW	100	———	———	———	IAAA-142457	Kazaoka et al. (2018)
GS-CB-3	16.30	-10.92	Shell	Shell fragments	8090 ± 30	8440–8630	8550	EF	DA	———	———	———	———	IAAA-142458	Kazaoka et al. (2018)
GS-CB-3	18.23	-12.85	Shell	*Tegillarca granosa* (Linnaeus)	8520 ± 30	9030–9260	9150	EF	RW	350	———	———	———	IAAA-142459	Kazaoka et al. (2018)
GS-CB-3	20.89	-15.51	Shell	Shell fragments	46500 ± 500	———	———	SH	Base	———	———	———	———	IAAA-142460	Kazaoka et al. (2018)
GS-CB-3	21.20	-15.82	Shell	Shell fragments	>53900	———	———	SH	Base	———	———	———	———	IAAA-142461	Kazaoka et al. (2018)
GS-CB-3	21.38	-16.00	Bulk sediments		32600 ± 160	———	———	SH	Base	———	———	———	———	IAAA-142462	Kazaoka et al. (2018)
GS-CB-4	12.70	-8.82	Shell	*Raeta pellicula* (Reeve)	1320 ± 20	790–920	870	DF	DA	———	———	———	———	IAAA-150477	Kazaoka et al. (2018)
GS-CB-4	18.26	-14.38	Shell	*Mactra chinensis* Philippi	7960 ± 30	8350–8500	8410	EF	DA	———	———	———	———	IAAA-153566	Kazaoka et al. (2018)
GS-CB-4	20.26	-16.38	Shell	*Mactra chinensis* Philippi	8300 ± 30	8730–8980	8870	EF	DA	———	———	———	———	IAAA-150478	Kazaoka et al. (2018)
GS-CB-4	26.66	-22.78	Shell	*Moerella* sp.	8780 ± 30	9390–9510	9450	EF	RW	250	———	———	———	IAAA-150479	Kazaoka et al. (2018)
GS-CB-4	37.22	-33.34	Wood		16000 ± 50	19120–19520	19320	MR	DA	———	———	———	———	IAAA-150480	Kazaoka et al. (2018)
GS-CB-5	13.16	-8.86	Shell	*Scapharca kagoshimensis* (Tokunaga)	410 ± 20	———	———	DF	DA	———	———	———	———	IAAA-153567	Kazaoka et al. (2018)
GS-CB-5	18.40	-14.10	Shell	*Lucinoma annulatum* (Reeve)	5500 ± 30	5770–5970	5890	DF	DA	———	———	———	———	IAAA-153568	Kazaoka et al. (2018)
GS-CB-6	6.03	-4.00	Shell	*Meretrix lusiria* (Röding)	7230 ± 30	7970–8160	8040	EF	RW	100	———	———	———	IAAA-171100	Kazaoka et al. (2018)
GS-CB-6	12.90	-10.87	Shell	*Mactra chinensis* Philippi	7500 ± 30	7880–8030	7960	EF	DA	———	———	———	———	IAAA-153569	Kazaoka et al. (2018)
GS-CB-6	17.15	-15.12	Shell	*Veremolpa micra* (Pilsbry)	8080 ± 30	8440–8620	8540	EF	DA	———	———	———	———	IAAA-153570	Kazaoka et al. (2018)
GS-CB-6	21.04	-19.01	Shell	*Cerithidea djadjariensis* (Martin)	8550 ± 30	9070–9300	9190	TF	DA	———	———	———	———	IAAA-153571	Kazaoka et al. (2018)
GS-CB-6	26.20	-24.17	Wood		10540 ± 40	12410–12630	12510	MR	DA	———	———	———	———	IAAA-153572	Kazaoka et al. (2018)
GS-CB-7	18.50	-14.11	Shell	Shell fragments	26370 ± 100	———	———	SH	Base	———	———	———	———	IAAA-153573	Kazaoka et al. (2018)
GS-CB-7	22.50	-18.11	Shell	Shell fragments	43010 ± 390	———	———	SH	Base	———	———	———	———	IAAA-153574	Kazaoka et al. (2018)
GS-CB-7	26.01	-21.62	Shell	*Ruditapes philippinarum* (Adams & Reeve)	46250 ± 480	———	———	SH	Base	———	———	———	———	IAAA-153575	Kazaoka et al. (2018)
GS-CB-8	11.33	-7.77	Shell	Shell fragments	610 ± 20	550–650	600	DF	DA	———	———	———	———	IAAA-162700	Kazaoka et al. (2018)
GS-CB-8	13.78	-10.22	Shell	Shell fragments	1960 ± 20	1870–1980	1910	DF	DA	———	———	———	———	IAAA-162701	Kazaoka et al. (2018)
GS-CB-8	16.71	-13.15	Shell	Shell fragments	3620 ± 20	3870–3980	3930	DF	DA	———	———	———	———	IAAA-162702	Kazaoka et al. (2018)
GS-CB-8	17.12	-13.56	Shell	Shell fragments	5290 ± 30	5950–6180	6080	DF	DA	———	———	———	———	IAAA-162703	Kazaoka et al. (2018)

GS-CB-8	17.85	-14.29	Shell	Shell fragments	8240 ± 30	9090–9400	9210	EF	RW	350	———	———	———	IAAA-162704	Kazaoka et al. (2018)
GS-CB-8	26.20	-22.64	Shell	Shell fragments	8730 ± 30	9560–9890	9680	EF	RW	350	———	———	———	IAAA-162705	Kazaoka et al. (2018)
GS-CB-8	29.34	-25.78	Shell	Shell fragments	8990 ± 30	9950–10230	10190	EF	RW	650	———	———	———	IAAA-162706	Kazaoka et al. (2018)
GS-CB-8	36.95	-33.39	Wood		9190 ± 30	9880–10130	10000	TF	DA	———	———	———	———	IAAA-162707	Kazaoka et al. (2018)
GS-CB-8	37.62	-34.06	Bulk sediments		9840 ± 40	10640–10960	10770	MR	DA	———	———	———	———	IAAA-162708	Kazaoka et al. (2018)
GS-CB-8	37.89	-34.33	Bulk sediments		10050 ± 40	10890–11180	11060	MR	DA	———	———	———	———	IAAA-162709	Kazaoka et al. (2018)
Hinode	4.10	-2.49	Shell	Shell fragments	680 ± 30	560–680	650	DF	DA	———	———	———	———	IAAA-153480	Kazaoka et al. (2018)
Hinode	9.65	-8.04	Shell	Shell fragments	7560 ± 30	8340–8410	8380	TF	DA	———	———	———	———	IAAA-153481	Kazaoka et al. (2018)
Hinode	17.35	-15.74	Shell	Shell fragments	8320 ± 30	9260–9440	9350	EF	RW	350	———	———	———	IAAA-153482	Kazaoka et al. (2018)
Hinode	27.54	-25.93	Shell	Shell fragments	8980 ± 30	9940–10230	10190	EF	RW	650	———	———	———	IAAA-153483	Kazaoka et al. (2018)
Hinode	35.21	-33.60	Plant		10320 ± 40	11190–11570	11310	MR	DA	———	———	———	———	IAAA-153484	Kazaoka et al. (2018)
Gyotoku	5.66	-2.59	Shell	Shell fragments	680 ± 20	570–670	660	DF	DA	———	———	———	———	IAAA-153485	Kazaoka et al. (2018)
Gyotoku	15.76	-12.69	Shell	Shell fragments	2660 ± 30	2740–2840	2770	DF	DA	———	———	———	———	IAAA-153486	Kazaoka et al. (2018)
Gyotoku	18.83	-15.76	Shell	Shell fragments	3190 ± 30	3360–3460	3410	DF	DA	———	———	———	———	IAAA-153487	Kazaoka et al. (2018)
Gyotoku	35.36	-32.29	Shell	Shell fragments	9150 ± 40	10230–10480	10300	EF	RW	250	———	———	———	IAAA-153488	Kazaoka et al. (2018)
Gyotoku	41.07	-38.00	Plant		9420 ± 40	10170–10380	10250	TF	DA	———	———	———	———	IAAA-153489	Kazaoka et al. (2018)
Hamada	7.35	-3.79	Shell	Shell fragments	1860 ± 20	1730–1870	1800	DF	DA	———	———	———	———	IAAA-162711	Kazaoka et al. (2018)
Hamada	9.75	-6.19	Shell	Shell fragments	2670 ± 20	2750–2840	2770	DF	DA	———	———	———	———	IAAA-170007	Kazaoka et al. (2018)
Hamada	10.25	-6.69	Shell	Shell fragments	2750 ± 20	2780–2920	2830	DF	DA	———	———	———	———	IAAA-170008	Kazaoka et al. (2018)
Hamada	17.35	-13.79	Bulk sediments		9650 ± 30	10440–10640	10540	MR	DA	———	———	———	———	IAAA-170009	Kazaoka et al. (2018)
Hamada	18.29	-14.73	Bulk sediments		10580 ± 40	11640–12060	11880	MR	DA	———	———	———	———	IAAA-170010	Kazaoka et al. (2018)
GS-KKW-1	3.54	-3.09	Plant		2500 ± 30	2490–2740	2590	MF	DA	———	———	———	———	IAAA-143691	Tanabe et al. (2022)
GS-KKW-1	5.75	-5.30	Plant		3690 ± 30	3960–4100	4030	MF	RW	1040	2990	-3	96	IAAA-143692	Tanabe et al. (2022)
GS-KKW-1	6.70	-6.25	Plant		3290 ± 30	3450–3580	3520	MF	RW	330	3190	-5	70	IAAA-143693	Tanabe et al. (2022)
GS-KKW-1	7.55	-7.10	Plant		3090 ± 30	3230–3380	3300	DF	DA	———	———	———	———	IAAA-150819	Tanabe et al. (2022)
GS-KKW-1	7.77	-7.32	Plant		3150 ± 30	3330–3450	3380	DF	RW	80	3300	-6	84	IAAA-143694	Tanabe et al. (2022)
GS-KKW-1	8.30	-7.85	Wood		3680 ± 30	3910–4090	4020	DF	RW	670	3350	-7	23	IAAA-150820	Tanabe et al. (2022)
GS-KKW-1	8.93	-8.48	Echinoderm		3580 ± 30	3380–3560	3470	DF	RW	130	3340	-7	12	IAAA-150821	Tanabe et al. (2022)
GS-KKW-1	11.98	-11.53	Plant		3180 ± 30	3360–3460	3410	DF	DA	———	———	———	———	IAAA-143695	Tanabe et al. (2022)
GS-KKW-1	14.62	-14.17	Shell	*Leucotina dianae* (Adams)	3800 ± 30	3640–3840	3750	DF	DA	———	———	———	———	IAAA-143740	Tanabe et al. (2022)
GS-KKW-1	15.98	-15.53	Shell	*Fulvia mutica* (Reeve)	3990 ± 30	3880–4100	4000	DF	DA	———	———	———	———	IAAA-150822	Tanabe et al. (2022)
GS-KKW-1	17.00	-16.55	Shell	*Macoma* cf. *tokyoensis* Makiyama	4920 ± 30	5110–5320	5260	DF	RW	670	4590	-20	8	IAAA-150823	Tanabe et al. (2022)
GS-KKW-1	17.25	-16.80	Plant		4160 ± 30	4610–4770	4710	DF	DA	———	———	———	———	IAAA-150824	Tanabe et al. (2022)
GS-KKW-1	18.44	-17.99	Shell	*Dosinella angulosa* (Philippi)	7300 ± 40	7660–7860	7760	EF	DA	———	———	———	———	IAAA-143741	Tanabe et al. (2022)
GS-KKW-1	21.07	-20.62	Plant		7480 ± 30	8280–8380	8310	EF	DA	———	———	———	———	IAAA-143696	Tanabe et al. (2022)
GS-KKW-1	22.94	-22.49	Shell	*Dosinella angulosa* (Philippi)	8460 ± 30	8980–9190	9070	EF	DA	———	———	———	———	IAAA-143742	Tanabe et al. (2022)
GS-KKW-1	25.85	-25.40	Plant		8650 ± 30	9540–9670	9590	EF	RW	210	9380	0	65	IAAA-143697	Tanabe et al. (2022)
GS-KKW-1	29.12	-28.67	Plant		8720 ± 30	9550–9790	9660	EF	DA	———	———	———	———	IAAA-143698	Tanabe et al. (2022)
GS-KKW-1	32.65	-32.20	Plant		8870 ± 30	9890–10170	10030	TF	DA	———	———	———	———	IAAA-143699	Tanabe et al. (2022)
GS-KKW-1	34.30	-33.85	Plant		9160 ± 30	10240–10400	10300	TF	DA	———	———	———	———	IAAA-143700	Tanabe et al. (2022)
GS-KKW-1	35.45	-35.00	Plant		9240 ± 30	10280–10510	10410	TF	DA	———	———	———	———	IAAA-143701	Tanabe et al. (2022)
GS-KKW-1	36.50	-36.05	Shell	*Crassostrea* sp.	9820 ± 40	10610–10920	10740	TF	DA	———	———	———	———	IAAA-150825	Tanabe et al. (2022)
GS-KKW-1	37.37	-36.92	Shell	*Trapezium liratum* (Reeve)	9980 ± 40	10790–11110	10970	TF	DA	———	———	———	———	IAAA-143743	Tanabe et al. (2022)
GS-KKW-1	38.30	-37.85	Plant		9800 ± 40	11180–11260	11220	TF	DA	———	———	———	———	IAAA-150826	Tanabe et al. (2022)
GS-KKW-1	39.05	-38.60	Plant		>53900	———	———	SH	Base	———	———	———	———	IAAA-143702	Tanabe et al. (2022)
GS-KKW-1	41.30	-40.85	Plant		47730 ± 560	———	———	SH	Base	———	———	———	———	IAAA-143703	Tanabe et al. (2022)
GS-KKW-1	42.14	-41.69	Plant		35680 ± 200	———	———	SH	Base	———	———	———	———	IAAA-143704	Tanabe et al. (2022)
GS-KKW-1	42.45	-42.00	Plant		50250 ± 760	———	———	SH	Base	———	———	———	———	IAAA-143705	Tanabe et al. (2022)
GS-KSW-1	6.70	-4.12	Plant		1890 ± 30	1740–1890	1840	MF	DA	———	———	———	———	IAAA-151520	Tanabe et al. (2022)
GS-KSW-1	7.50	-4.92	Plant		4880 ± 30	5590–5660	5620	DF	RW	100	5520	-8	42	IAAA-151521	Tanabe et al. (2022)
GS-KSW-1	7.50	-4.92	Shell	*Veremolpa micra* (Pilsbry)	5300 ± 30	5580–5730	5650	DF	RW	100	5550	-8	42	IAAA-151522	Tanabe et al. (2022)
GS-KSW-1	9.20	-6.62	Plant		4880 ± 30	5590–5660	5620	DF	DA	———	———	———	———	IAAA-151523	Tanabe et al. (2022)
GS-KSW-1	11.45	-8.87	Plant		5730 ± 30	6450–6570	6520	DF	RW	170	6350	-12	10	IAAA-151524	Tanabe et al. (2022)
GS-KSW-1	11.45	-8.87	Shell	*Macoma* cf. *tokyoensis* Makiyama	5900 ± 30	6260–6390	6320	DF	DA	———	———	———	———	IAAA-151525	Tanabe et al. (2022)
GS-KSW-1	13.10	-10.52	Shell	*Paphia undulata* (Born)	6880 ± 30	7320–7460	7400	DF	DA	———	———	———	———	IAAA-151526	Tanabe et al. (2022)
GS-KSW-1	14.65	-12.07	Shell	*Dosinella angulosa* (Philippi)	7620 ± 30	7990–8160	8080	EF	DA	———	———	———	———	IAAA-151527	Tanabe et al. (2022)
GS-KSW-1	15.50	-12.92	Shell	*Dosinella angulosa* (Philippi)	7940 ± 30	8330–8480	8400	EF	DA	———	———	———	———	IAAA-151528	Tanabe et al. (2022)
GS-KSW-1	16.96	-14.38	Plant		8030 ± 30	8860–9010	8910	EF	RW	380	8530	-6	27	IAAA-151529	Tanabe et al. (2022)
GS-KSW-1	18.55	-15.97	Plant		7990 ± 30	8750–9000	8880	EF	RW	250	8630	-7	24	IAAA-151530	Tanabe et al. (2022)
GS-KSW-1	19.85	-17.27	Plant		8000 ± 30	8760–9010	8880	EF	RW	80	8800	-3	18	IAAA-151531	Tanabe et al. (2022)
GS-KSW-1	21.40	-18.82	Plant		8190 ± 30	9030–9260	9130	EF	RW	250	8880	-2	50	IAAA-151532	Tanabe et al. (2022)
GS-KSW-1	22.15	-19.57	Plant		8030 ± 30	8860–9010	8910	EF	DA	———	———	———	———	IAAA-151533	Tanabe et al. (2022)
GS-KSW-1	24.10	-21.52	Plant		8220 ± 30	9080–9290	9190	TF	DA	———	———	———	———	IAAA-151534	Tanabe et al. (2022)
GS-KSW-1	24.70	-22.12	Plant		8620 ± 30	9530–9630	9560	TF	RW	290	9270	3	54	IAAA-160276	Tanabe et al. (2022)
GS-KSW-1	25.50	-22.92	Plant		8340 ± 30	9290–9450	9370	TF	DA	———	———	———	———	IAAA-151535	Tanabe et al. (2022)
GS-KSW-1	26.70	-24.12	Plant		8680 ± 30	9550–9690	9620	TF	RW	130	9490	2	61	IAAA-151536	Tanabe et al. (2022)
GS-KSW-1	27.40	-24.82	Plant		8680 ± 30	9550–9690	9620	TF	RW	80	9540	1	26	IAAA-160277	Tanabe et al. (2022)
GS-KSW-1	28.50	-25.92	Plant		8700 ± 30	9550–9710	9640	TF	DA	———	———	———	———	IAAA-151537	Tanabe et al. (2022)
GS-KSW-1	30.06	-27.48	Plant		8750 ± 30	9600–9890	9730	TF	DA	———	———	———	———	IAAA-151538	Tanabe et al. (2022)
GS-KSW-1	30.90	-28.32	Plant		8920 ± 30	9920–10090	10040	TF	RW	130	9910	1	6	IAAA-160278	Tanabe et al. (2022)
GS-KSW-1	31.72	-29.14	Plant		8860 ± 30	9880–10160	10020	TF	DA	———	———	———	———	IAAA-151539	Tanabe et al. (2022)
GS-KSW-1	33.80	-31.22	Plant		9170 ± 40	10240–10430	10330	MR	DA	———	———	———	———	IAAA-151540	Tanabe et al. (2022)
GS-KNH-1	4.30	1.56	Plant		3620 ± 30	3840–3990	3930	MF	DA	———	———	———	———	IAAA-143707	Tanabe et al. (2022)
GS-KNH-1	6.40	-0.54	Plant		3740 ± 30	3980–4160	4100	MF	DA	———	———	———	———	IAAA-143708	Tanabe et al. (2022)
GS-KNH-1	9.81	-3.95	Plant		7080 ± 30	7850–7960	7900	EF	DA	———	———	———	———	IAAA-143709	Tanabe et al. (2022)
GS-KNH-1	11.37	-5.51	Plant		7340 ± 30	8030–8200	8140	EF	DA	———	———	———	———	IAAA-143710	Tanabe et al. (2022)
GS-KNH-1	12.26	-6.40	Plant		7470 ± 30	8270–8370	8300	EF	DA	———	———	———	———	IAAA-143711	Tanabe et al. (2022)

GS-KNH-1	14.33	-8.47	Plant		7920 ± 30	8630–8800	8740	EF	RW	350	8390	0	50	IAAA-143712	Tanabe et al. (2022)
GS-KNH-1	17.70	-11.84	Plant		7730 ± 30	8430–8560	8510	EF	DA	———	———	———	———	IAAA-143713	Tanabe et al. (2022)
GS-KNH-1	18.17	-12.31	Plant		7880 ± 30	8590–8780	8670	TF	DA	———	———	———	———	IAAA-143714	Tanabe et al. (2022)
GS-KNH-1	19.60	-13.74	Plant		8010 ± 30	8770–9010	8890	TF	RW	120	8770	0	10	IAAA-160279	Tanabe et al. (2022)
GS-KNH-1	21.10	-15.24	Plant		7970 ± 30	8700–8990	8860	TF	DA	———	———	———	———	IAAA-143715	Tanabe et al. (2022)
GS-KNH-1	22.50	-16.64	Plant		8050 ± 30	8950–9030	8990	TF	DA	———	———	———	———	IAAA-143716	Tanabe et al. (2022)
GS-KNH-1	25.33	-19.47	Plant		8130 ± 30	9000–9130	9060	TF	DA	———	———	———	———	IAAA-143717	Tanabe et al. (2022)
GS-KNH-2	2.27	7.16	Plant		3250 ± 30	3440–3560	3470	MF	DA	———	———	———	———	IAAA-151512	Tanabe et al. (2022)
GS-KNH-2	4.10	5.33	Plant		4040 ± 30	4420–4580	4500	MF	DA	———	———	———	———	IAAA-151513	Tanabe et al. (2022)
GS-KNH-2	6.71	2.72	Plant		6080 ± 30	6850–7010	6940	MF	DA	———	———	———	———	IAAA-151514	Tanabe et al. (2022)
GS-KNH-2	8.15	1.28	Plant		6430 ± 30	7290–7420	7360	MF	RW	200	7160	-1	9	IAAA-151515	Tanabe et al. (2022)
GS-KNH-2	9.65	-0.22	Plant		6430 ± 30	7290–7420	7360	MF	DA	———	———	———	———	IAAA-151516	Tanabe et al. (2022)
GS-KNH-2	13.30	-3.87	Plant		7540 ± 30	8330–8410	8370	DF	RW	430	7940	-1	58	IAAA-160280	Tanabe et al. (2022)
GS-KNH-2	14.40	-4.97	Plant		7550 ± 30	8340–8410	8380	DF	RW	280	8100	0	76	IAAA-151517	Tanabe et al. (2022)
GS-KNH-2	15.10	-5.67	Plant		7920 ± 30	8630–8800	8740	TF	RW	500	8240	0	55	IAAA-151518	Tanabe et al. (2022)
GS-KNH-2	16.23	-6.80	Plant		7570 ± 30	8350–8410	8390	TF	DA	———	———	———	———	IAAA-151519	Tanabe et al. (2022)
GS-KNH-2	16.75	-7.32	Plant		7740 ± 30	8440–8590	8520	TF	DA	———	———	———	———	IAAA-151541	Tanabe et al. (2022)
GS-KNH-2	17.90	-8.47	Plant		7770 ± 30	8510–8600	8560	TF	DA	———	———	———	———	IAAA-151542	Tanabe et al. (2022)
GS-KNH-2	18.80	-9.37	Plant		7870 ± 30	8590–8770	8650	TF	DA	———	———	———	———	IAAA-160281	Tanabe et al. (2022)
GS-KNH-2	19.72	-10.29	Plant		7970 ± 30	8700–8990	8860	TF	RW	130	8730	0	42	IAAA-151543	Tanabe et al. (2022)
GS-KNH-2	20.50	-11.07	Plant		8000 ± 30	8760–9010	8880	TF	RW	80	8800	3	7	IAAA-160282	Tanabe et al. (2022)
GS-KNH-2	21.45	-12.02	Plant		7980 ± 30	8720–9000	8870	MR	DA	———	———	———	———	IAAA-151544	Tanabe et al. (2022)
GS-KNH-3	2.80	8.53	Plant		4210 ± 30	4690–4760	4740	MF	DA	———	———	———	———	IAAA-151545	Tanabe et al. (2022)
GS-KNH-3	7.95	3.38	Plant		5740 ± 30	6460–6630	6540	MF	DA	———	———	———	———	IAAA-151546	Tanabe et al. (2022)
GS-KNH-3	8.50	2.83	Plant		6010 ± 30	6780–6940	6850	MF	DA	———	———	———	———	IAAA-151547	Tanabe et al. (2022)
GS-KNH-3	10.20	1.13	Plant		6740 ± 30	7570–7660	7600	MF	DA	———	———	———	———	IAAA-151548	Tanabe et al. (2022)
GS-KNH-3	10.80	0.53	Plant		7710 ± 30	8420–8550	8490	MF	DA	———	———	———	———	IAAA-151549	Tanabe et al. (2022)
GS-KNH-4	4.95	1.85	Plant		3480 ± 30	3650–3840	3760	MF	DA	———	———	———	———	IAAA-190154	Tanabe et al. (2022)
GS-KNH-4	6.20	0.60	Plant		3540 ± 30	3720–3900	3830	MF	DA	———	———	———	———	IAAA-190155	Tanabe et al. (2022)
GS-KNH-4	9.30	-2.50	Plant		7320 ± 30	8040–8180	8110	EF	RW	100	8010	2	55	IAAA-190156	Tanabe et al. (2022)
GS-KNH-4	10.10	-3.30	Plant		7200 ± 30	7950–8150	8000	EF	DA	———	———	———	———	IAAA-190157	Tanabe et al. (2022)
GS-KNH-4	12.00	-5.20	Plant		7590 ± 30	8360–8430	8400	EF	RW	20	8380	3	48	IAAA-190158	Tanabe et al. (2022)
GS-KNH-4	12.50	-5.70	Plant		7410 ± 30	8180–8320	8260	EF	DA	———	———	———	———	IAAA-190159	Tanabe et al. (2022)
GS-KNH-4	13.40	-6.60	Plant		8290 ± 30	9140–9420	9310	EF	RW	850	8460	1	58	IAAA-190160	Tanabe et al. (2022)
GS-KNH-4	14.60	-7.80	Plant		9710 ± 30	11100–11220	11170	EF	RW	2810	8360	0	18	IAAA-190161	Tanabe et al. (2022)
GS-KNH-4	15.30	-8.50	Plant		7520 ± 30	8220–8400	8360	EF	DA	———	———	———	———	IAAA-190162	Tanabe et al. (2022)
GS-YKH-1	3.30	0.80	Plant		270 ± 20	160–430	310	MF	DA	———	———	———	———	IAAA-190134	Tanabe et al. (2022)
GS-YKH-1	3.80	0.30	Plant		3070 ± 20	3220–3360	3290	MF	RW	300	2990	2	82	IAAA-190135	Tanabe et al. (2022)
GS-YKH-1	5.10	-1.00	Plant		2950 ± 20	3010–3170	3110	MF	DA	———	———	———	———	IAAA-190136	Tanabe et al. (2022)
GS-YKH-1	7.50	-3.40	Plant		4780 ± 30	5470–5590	5520	MF	DA	———	———	———	———	IAAA-190137	Tanabe et al. (2022)
GS-YKH-1	8.70	-4.60	Plant		4860 ± 30	5490–5650	5600	DF	DA	———	———	———	———	IAAA-190138	Tanabe et al. (2022)
GS-YKH-1	9.50	-5.40	Plant		5620 ± 30	6310–6470	6400	DF	DA	———	———	———	———	IAAA-190139	Tanabe et al. (2022)
GS-YKH-1	11.80	-7.70	Plant		6640 ± 30	7470–7580	7530	DF	DA	———	———	———	———	IAAA-190140	Tanabe et al. (2022)
GS-YKH-1	13.80	-9.70	Plant		7350 ± 30	8040–8290	8170	DF	RW	360	7810	-7	8	IAAA-190141	Tanabe et al. (2022)
GS-YKH-1	15.70	-11.60	Plant		7110 ± 30	7870–8000	7950	DF	DA	———	———	———	———	IAAA-190142	Tanabe et al. (2022)
GS-YKH-1	17.25	-13.15	Plant		7200 ± 30	7950–8150	8000	EF	DA	———	———	———	———	IAAA-190143	Tanabe et al. (2022)
GS-YKH-1	19.40	-15.30	Plant		8210 ± 30	9030–9280	9180	EF	RW	860	8320	-8	2	IAAA-190144	Tanabe et al. (2022)
GS-YKH-1	21.80	-17.70	Plant		8220 ± 30	9030–9290	9190	EF	RW	640	8550	-9	0	IAAA-190145	Tanabe et al. (2022)
GS-YKH-1	23.70	-19.60	Plant		7940 ± 30	8640–8980	8790	EF	DA	———	———	———	———	IAAA-190146	Tanabe et al. (2022)
GS-YKH-1	25.80	-21.70	Plant		8020 ± 30	8780–9010	8890	EF	DA	———	———	———	———	IAAA-190147	Tanabe et al. (2022)
GS-YKH-1	27.20	-23.10	Plant		8680 ± 30	9550–9690	9620	TF	DA	———	———	———	———	IAAA-190148	Tanabe et al. (2022)
GS-YKH-1	28.80	-24.70	Shell	*Corbicula japonica*	9290 ± 30	10010–10210	10150	TF	RW	270	9880	4	2	IAAA-190149	Tanabe et al. (2022)
GS-YKH-1	29.20	-25.10	Plant		8840 ± 30	9740–10150	9920	TF	DA	———	———	———	———	IAAA-190150	Tanabe et al. (2022)
GS-YKH-1	30.60	-26.50	Plant		8950 ± 30	9920–10210	10130	TF	DA	———	———	———	———	IAAA-190151	Tanabe et al. (2022)
GS-YKH-1	31.10	-27.00	Plant		8990 ± 30	9950–10230	10190	MR	DA	———	———	———	———	IAAA-190152	Tanabe et al. (2022)
GS-YKH-1	32.10	-28.00	Plant		9010 ± 30	10170–10230	10210	MR	DA	———	———	———	———	IAAA-190153	Tanabe et al. (2022)
GS-TOT-1	2.10	-0.22	Plant		2900 ± 20	2960–3140	3030	MT	DA	———	———	———	———	IAAA-190169	Tanabe et al. (2022)
GS-TOT-1	3.10	-1.22	Plant		3570 ± 20	3830–3960	3870	DF	RW	680	3190	0	71	IAAA-190170	Tanabe et al. (2022)
GS-TOT-1	5.50	-3.62	Plant		3640 ± 30	3870–4080	3950	DF	RW	500	3450	-4	8	IAAA-190171	Tanabe et al. (2022)
GS-TOT-1	7.40	-5.52	Plant		3360 ± 30	3490–3690	3600	DF	DA	———	———	———	———	IAAA-190172	Tanabe et al. (2022)
GS-TOT-1	9.75	-7.87	Plant		4180 ± 30	4590–4840	4720	DF	DA	———	———	———	———	IAAA-190173	Tanabe et al. (2022)
GS-TOT-2	1.20	1.86	Plant		1810 ± 20	1640–1820	1750	MF	DA	———	———	———	———	IAAA-190163	Tanabe et al. (2022)
GS-TOT-2	2.50	0.56	Plant		2290 ± 20	2210–2350	2330	MF	DA	———	———	———	———	IAAA-190164	Tanabe et al. (2022)
GS-TOT-2	3.80	-0.74	Plant		2840 ± 20	2870–3000	2940	MF	DA	———	———	———	———	IAAA-190165	Tanabe et al. (2022)
GS-TOT-2	5.60	-2.54	Plant		5970 ± 30	6730–6890	6800	DF	DA	———	———	———	———	IAAA-190166	Tanabe et al. (2022)
GS-TOT-2	7.30	-4.24	Plant		7820 ± 30	8540–8680	8600	DF	RW	640	7960	0	10	IAAA-190167	Tanabe et al. (2022)
GS-TOT-2	8.30	-5.24	Plant		8030 ± 30	8780–9010	8910	DF	RW	770	8140	0	8	IAAA-190168	Tanabe et al. (2022)

Table S3

Sedimentary facies	Average age offset (1σ range) (yr)	
	Shell	Plant
MF (modern fluvial)	———	310 ± 260
MT (modern tidal flat)	———	1950 ± 610
DF (delta front)	500 ± 680	600 ± 640
SP (spit)	1710 ± 1340	———
EF (estuary front)	460 ± 320	680 ± 800
TF (tidal flat)	———	280 ± 280
MR (meandering river)	———	410 ± 240

Table S4

Core	Sedimentary facies	All age	Reworked age	Reworked %	Average age offset (yr)
GS-KBH-1	MF	7	3	43	103
GS-KBH-1	DF	29	14	48	362
GS-KBH-1	EF	12	8	67	513
GS-KBH-1	TF	3	1	33	20
GS-KBH-1	MR	5	1	20	150
GS-KS-1	MF	1	0	0	0
GS-KS-1	DF	11	9	82	1665
GS-KS-1	EF	2	1	50	1050
GS-KS-1	MR	3	0	0	0
GS-MUS-1	MF	3	1	33	3280
GS-MUS-1	DF	13	4	31	1042
GS-MUS-1	EF	7	0	0	0
GS-MHI-1	MF	2	1	50	340
GS-MHI-1	DF	33	19	58	310
GS-MHI-1	EF	5	3	60	673
GS-MHI-1	TF	3	1	33	370
GS-MHI-1	MR	10	4	40	455
GS-SK-1	MF	3	1	33	370
GS-SK-1	DF	6	3	50	913
GS-SK-1	EF	7	2	29	1285
GS-SK-1	TF	8	2	25	200
GS-SK-1	MR	3	0	0	0
GS-SMB-1	MF	2	0	0	0
GS-SMB-1	DF	6	2	33	895
GS-SMB-1	EF	17	9	53	397
TN	MT	1	0	0	0
TN	DF	4	1	25	470
TN	EF	3	1	33	580
MZ	MF	1	0	0	0
MZ	SP	9	4	44	1102
MZ	EF	1	0	0	0
MZ	TF	1	0	0	0
MZ	MR	3	0	0	0
GS-AHH-1	MT	1	0	0	0
GS-AHH-1	DF	7	2	29	290
GS-AHH-1	EF	4	1	25	370
GS-AHH-1	TF	5	2	40	290
HA	DF	6	2	33	1790
HA	EF	1	0	0	0
HA	TF	4	0	0	0
HA	MR	3	0	0	0
GS-KNJ-1	MF	1	0	0	0
GS-KNJ-1	DF	4	0	0	0
GS-KNJ-1	SP	16	10	63	2217
GS-KNJ-1	TF	3	0	0	0
GS-KNJ-1	MR	6	0	0	0
GS-AMG-1	MT	4	2	50	2185
GS-AMG-1	DF	8	2	25	685

GS-AMG-1	EF	13	9	69	636
GS-AMG-1	TF	6	2	33	240
GS-AMG-1	MR	6	2	33	525
GS-KTS-1	MT	1	1	100	1470
GS-KTS-1	SP	9	3	33	806
GS-KTS-1	EF	7	1	14	110
GS-KTS-1	TF	3	2	67	1055
SZ	DF	3	0	0	0
GS-KM-1	DF	10	0	0	0
GS-KM-1	EF	1	1	100	20
GS-KM-1	TF	2	0	0	0
GS-KM-1	MR	8	1	13	110
DK	DF	9	1	11	320
DK	EF	3	1	33	260
DK	TF	1	0	0	0
DK	MR	7	3	43	543
GS-ISH-1	MT	2	0	0	0
GS-ISH-1	DF	6	1	17	1840
GS-ISH-1	EF	14	5	36	622
GS-ISH-1	MR	7	1	14	160
GS-KSM-1	DF	7	0	0	0
GS-KSM-1	EF	9	1	11	420
GS-KSM-1	TF	2	1	50	320
GS-KSM-1	MR	6	0	0	0
GS-KWS-1	DF	11	2	18	290
GS-KWS-1	EF	1	0	0	0
GS-KWS-1	MR	7	0	0	0
GS-KSO-1	MF	3	1	33	200
GS-KSO-1	DF	10	1	10	420
GS-KSO-1	EF	2	0	0	0
GS-KSO-1	TF	3	2	67	105
GS-KSO-1	MR	3	0	0	0
GS-SSS-1	MF	3	0	0	0
GS-SSS-1	DF	9	3	33	423
GS-SSS-1	EF	4	1	25	2790
GS-SSS-1	TF	2	0	0	0
GS-TKT-1	MF	2	0	0	0
GS-TKT-1	DF	31	27	87	266
GS-TKT-1	EF	1	0	0	0
GS-TKT-1	TF	6	2	33	280
GS-TKT-1	MR	3	0	0	0
GS-FB-2	MF	3	0	0	0
GS-FB-2	DF	3	0	0	0
GS-FB-2	EF	3	0	0	0
GS-FB-2	TF	1	0	0	0
GS-FB-2	MR	1	0	0	0
GS-FB-3	MF	3	0	0	0
GS-FB-4	DF	3	0	0	0
GS-FB-4	EF	2	0	0	0
GS-FB-4	TF	1	0	0	0
GS-NS-1	DF	2	0	0	0
GS-NS-1	EF	2	1	50	150

GS-NS-1	TF	1	0	0	0
GS-CB-2	DF	4	0	0	0
GS-CB-2	TF	2	0	0	0
GS-CB-3	TF	2	0	0	0
GS-CB-3	EF	5	2	40	225
GS-CB-4	DF	1	0	0	0
GS-CB-4	EF	3	1	33	250
GS-CB-4	MR	1	0	0	0
GS-CB-5	DF	2	0	0	0
GS-CB-6	EF	3	1	33	100
GS-CB-6	TF	1	0	0	0
GS-CB-6	MR	1	0	0	0
GS-CB-8	DF	4	0	0	0
GS-CB-8	EF	3	3	100	450
GS-CB-8	TF	1	0	0	0
GS-CB-8	MR	2	0	0	0
Hinode	DF	1	0	0	0
Hinode	EF	2	2	100	500
Hinode	TF	1	0	0	0
Hinode	MR	1	0	0	0
Gyotoku	DF	3	0	0	0
Gyotoku	EF	1	1	100	250
Gyotoku	TF	1	0	0	0
Hamada	DF	3	0	0	0
Hamada	MR	2	0	0	0
GS-KKW-1	MF	3	2	67	685
GS-KKW-1	DF	9	4	44	387
GS-KKW-1	EF	5	1	20	210
GS-KKW-1	TF	6	0	0	0
GS-KSW-1	MF	1	0	0	0
GS-KSW-1	DF	6	3	50	123
GS-KSW-1	EF	7	4	57	240
GS-KSW-1	TF	9	4	44	157
GS-KSW-1	MR	1	0	0	0
GS-KNH-1	MF	2	0	0	0
GS-KNH-1	EF	5	1	20	350
GS-KNH-1	TF	5	1	20	120
GS-KNH-2	MF	5	1	20	200
GS-KNH-2	DF	2	2	100	355
GS-KNH-2	TF	7	3	43	236
GS-KNH-2	MR	1	0	0	0
GS-KNH-3	MF	5	0	0	0
GS-KNH-4	MF	2	0	0	0
GS-KNH-4	EF	7	4	57	945
GS-YKH-1	MF	4	1	25	300
GS-YKH-1	DF	5	1	20	360
GS-YKH-1	EF	5	2	40	750
GS-YKH-1	TF	4	1	25	270
GS-YKH-1	MR	2	0	0	0
GS-TOT-1	MT	1	0	0	0
GS-TOT-1	DF	4	2	50	590
GS-TOT-2	MF	3	0	0	0

GS-TOT-2	DF	3	2	67	705

Table S5

Sedimentary facies	All age	Reworked age	Reworked %	Average age offset (1σ range) (yr)
MF (modern fluvial)	59	11	19	310 ± 260
MT (modern tidal flat)	10	3	30	1950 ± 610
DF (delta front)	278	107	38	540 ± 670
SP (spit)	34	17	50	1710 ± 1340
EF (estuary front)	167	67	40	550 ± 580
TF (tidal flat)	94	24	26	280 ± 280
MR (meandering river)	92	12	13	410 ± 240
Subtidal	445	174	39	550 ± 630
Intertidal	104	27	26	470 ± 620
Terrestrial	151	23	15	360 ± 250

Table S6

Core	Sedimentary facies	Depositional duration (yr)	Average age offset (yr)
GS-KBH-1	MF	560	103
GS-KBH-1	DF	3600	362
GS-KBH-1	EF	1660	514
GS-KS-1	DF	2480	1666
GS-MUS-1	DF	940	1043
GS-MHI-1	DF	2160	311
GS-MHI-1	MR	420	455
GS-SK-1	DF	420	913
GS-SK-1	EF	740	1285
GS-SK-1	TF	860	200
GS-SMB-1	DF	170	895
GS-SMB-1	EF	3070	398
MZ	SP	900	1103
GS-AHH-1	DF	1850	290
GS-AHH-1	TF	30	290
HA	DF	3450	1790
GS-KNJ-1	SP	5870	2217
GS-AMG-1	DF	20	685
GS-AMG-1	EF	170	637
GS-AMG-1	TF	260	240
GS-AMG-1	MR	1900	525
GS-KTS-1	SP	200	807
GS-KTS-1	TF	260	1055
DK	MR	570	543
GS-ISH-1	EF	3430	622
GS-KWS-1	DF	240	290
GS-KSO-1	TF	80	105
GS-SSS-1	DF	820	423
GS-TKT-1	DF	790	266
GS-TKT-1	TF	260	280
GS-KKW-1	MF	200	685
GS-KKW-1	DF	1290	388
GS-KSW-1	DF	830	123
GS-KSW-1	EF	350	240
GS-KSW-1	TF	640	158
GS-KNH-2	DF	160	355
GS-KNH-2	TF	560	237
GS-KNH-4	EF	350	945
GS-YKH-1	EF	230	750
GS-TOT-1	DF	260	590
GS-TOT-2	DF	180	705
